# A cascade synthesis of 2-aminothiochromones from 2-fluorophenyl ketone and thiocarbonyldiimidazole

**DOI:** 10.3762/bjoc.22.92

**Published:** 2026-08-12

**Authors:** Kai Zhang, Haofan Shao, Kangjun Liao, Ying Xu, Shimei Du, Yanfang Zhao, Gang Li, Wenzhen Xu, Haihong Huang, Peng Li

**Affiliations:** 1 Key Laboratory of Structure-Based Drug Design & Discovery of Ministry of Education, School of Pharmaceutical Engineering, Shenyang Pharmaceutical University, Shenyang 110016, P. R. China; 2 Institute of Materia Medica, Chinese Academy of Medical Sciences and Peking Union Medical College, Beijing 100050, P. R. China; 3 Qingdao BAHEAL Pharmaceutical Co., Ltd., Qingdao 266200, P. R. China; 4 State Key Laboratory of Respiratory Health and Multimorbidity, Beijing Key Laboratory for Key Technologies in Tuberculosis Prevention and Control, Beijing 100050, P. R. China; 5 State Key Laboratory of Digestive Health, Beijing, 100050, P. R. China

**Keywords:** cascade synthesis, TCDI, thiochromone

## Abstract

A highly efficient cascade synthesis of various substituted 2-aminothiochromones was developed. The commercially available thiocarbonyldiimidazole (TCDI) acted as a key precursor in the construction of a sulfur-containing scaffold from 2-fluorophenyl ketone. Subsequent conjugated addition–elimination resulted in the corresponding 2-aminothiochromones under mild conditions. This simple protocol tolerates a broad range of functional groups and provides concise access to various 2-aminothiochromones in good to excellent yields (up to 94%).

## Introduction

Sulfur-containing motifs are particularly important owing to their widespread applications and diverse biological activities [1]. As isosteric analogs of chromones [2], in which the oxygen atom is replaced by a sulfur atom, thiochromones are widely present in various natural products, functional materials, and biologically active molecules [3–4], such as MAO-B inhibitor [5], anticancer [6], antibacterial [7], and antimalarial compounds [8]. More interestingly, thiochromone analogues showed more potent activity than their oxygen-containing counterparts [9]. For example, some thiochromones demonstrate enhanced steroid sulfatase inhibitory activity compared to their chromone analogues, highlighting their potential as more potent bioactive agents [10]. Furthermore, oxidation of the sulfur atom in thiochromones yields sulfoxide or sulfone derivatives that also possess diverse biological activities [4]. Beyond pharmaceutical applications, thiochromones have also been widely used in materials science. For instance, they can serve as photolabile protecting groups [11] and a photoinduced fluorescence [12] and OLED exciton utilization efficiency improver [13]. Owing to their widespread applications, diverse biological activities, and utility as key building blocks in both pharmaceuticals and materials science, thiochromones have attracted considerable interest [14]. This has spurred the development of synthetic approaches and driven sustained investigations into their synthesis over recent decades.

**Scheme 1:**
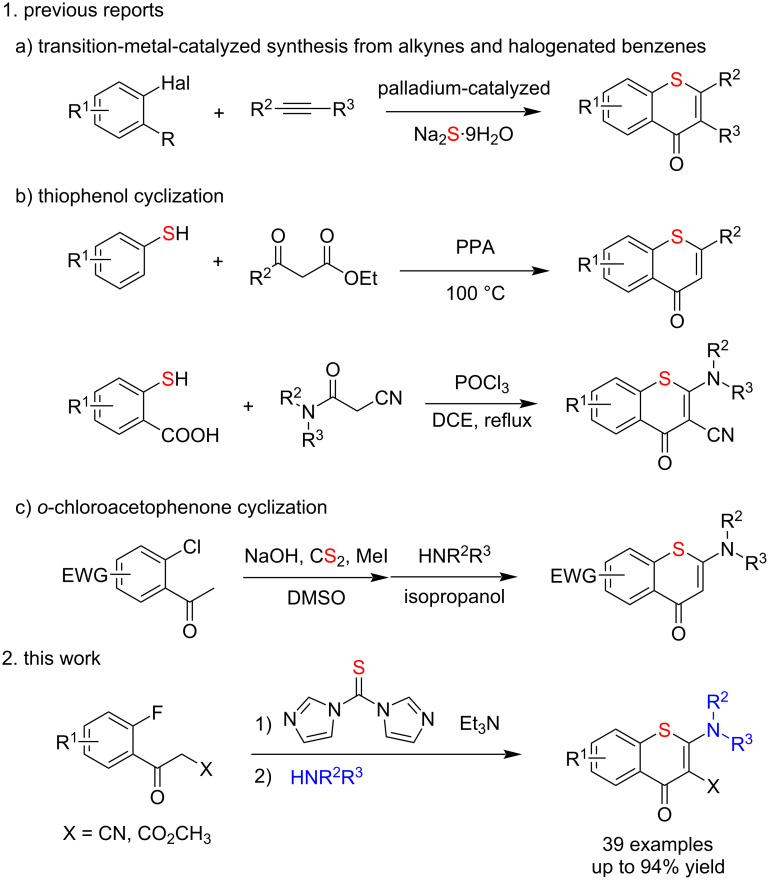
Synthesis of thiochromones.

Despite the structural similarity of the sulfur-containing thiochromone scaffold to the extensively developed chromones, synthetic methods for thiochromones remain very limited [2]. This disparity exists because, unlike the well-established synthesis of chromones, the construction of the thiochromone is hindered by several challenges. Compared to oxygen, the sulfur lone pair electrons exhibit a greater degree of delocalization into π-systems, which attenuates its inherent reactivity [1]. These electronic factors, combined with the limited availability of suitable substrates, present significant synthetic obstacles. Current synthetic approaches to thiochromones primarily involve two strategies: transition-metal-catalyzed reaction of aryl halides and cyclization of thiophenol derivatives. The transition-metal-catalyzed carbonylation reaction of aryl halides represents an attractive strategy for thiochromone synthesis due to its atom economy and straightforward construction of the essential carbonyl-containing framework [15–18]. Among these methods, the palladium-catalyzed multicomponent synthesis has been extensively studied. This reaction typically employs aryl halides and various alkynes as substrates, with sodium sulfide serving as the sulfur source (Scheme 1a) [19–21]. Another well-established method is the cyclization of thiophenol derivatives (Scheme 1b). A classic example is the condensation of substituted thiophenols with β-keto esters in the presence of polyphosphoric acid [22]. A gentle route was developed by Simon, which involved the reaction of thiosalicylic acids with 2-substituted *N,N*-dialkylacetamides in the presence of phosphorus oxychloride to afford 2-aminothiochromones [23]. Despite the wide application of the above approaches, the synthesis of 2-aminothiochromones often requires harsh conditions, such as high temperatures or strong acids [24–25].

Although existing methods have enriched the synthesis of thiochromones, their narrow substrate scope and limited functional group compatibility hinder the efficient development of compound libraries for biological screening [26]. Driven by the interesting pharmacological potential of thiochromones, our group has a long-standing interest in their synthesis and bioactivity. In 2018, we reported a strategy for the construction of the 2-aminothiochromone scaffold with the aim of identifying antitubercular agents [27]. This approach involved the reaction of *o*-chloroacetophenone bearing an electron-withdrawing group with carbon disulfide to furnish the core scaffold, followed by amine introduction via nucleophilic aromatic substitution of the thioether in 2-position (Scheme 1c). We subsequently developed a more efficient protocol to access 2-aminothiochromones via a sulfoxide intermediate [28]. Then, we established a novel strategy for constructing 2-arylthiochromones using a combined Lewis acid and palladium(II) catalyst system [29]. As a part of our ongoing efforts to synthesize versatile thiochromones, this study was to establish a more economical and efficient synthetic route.

Recently, Imming and co-workers developed a method for preparing *N,N*-dialkylthioureas, which serve as key intermediates in benzothiazinone synthesis, using thiocarbonyldiimidazole (TCDI) as a sulfur source [30]. Inspired by these works and driven by our continued interest in thiochromone synthesis, we wondered whether it would be possible to combine thiochromone construction with amino functionalization in a cascade synthesis mode, wherein TCDI not only serves as a source of sulfur, but also provides an imidazole that acts as a reactive handle for further functionalization, thus maximizing atomic economy. Herein, we disclose a cascade synthesis of 2-aminothiochromone with a highly flexible substitution pattern.

## Results and Discussion

Initially, we selected 3-(2-fluorophenyl)-3-oxopropanenitrile (**1a**), morpholine (**2a**), and TCDI as model substrates to explore the feasibility of our hypothesis, since fluorine is the preferred leaving group in nucleophilic aromatic substitution (S_N_Ar) reactions (Table 1). In the absence of a base, the substrate **1a** remained unchanged when refluxed in dichloromethane (DCM). The addition of triethylamine (Et₃N) led to only a trace amount of product. Switching the solvent to tetrahydrofuran (THF) significantly increased the yield to 56%, suggesting that both a base and a higher reaction temperature are necessary for the scaffold construction. Employing acetonitrile (MeCN), with a higher boiling point, shortened the reaction time while maintaining a comparable yield. Carrying out the reaction in dimethylformamide (DMF) at 100 °C proved optimal, delivering product **3a** in 78% yield. Keeping DMF as the optimal solvent, subsequent base screening revealed that the yield was maintained when Et_3_N was replaced by *N*,*N*-diisopropylethylamine (DIPEA). In contrast, the use of 1,8-diazabicyclo[5.4.0]undec-7-ene (DBU) or potassium *tert*-butoxide failed to obtain **3a**, and potassium carbonate diminished the yield by almost half. The current results indicated that Et₃N was the optimal base for this reaction. Finally, we found that reducing the amount of TCDI to 1.3 equivalents under the optimal Et_3_N/DMF conditions retained a high yield, thus establishing the standard conditions for the subsequent amine scope investigation.

**Table 1:** Optimization of reaction conditions.^a^

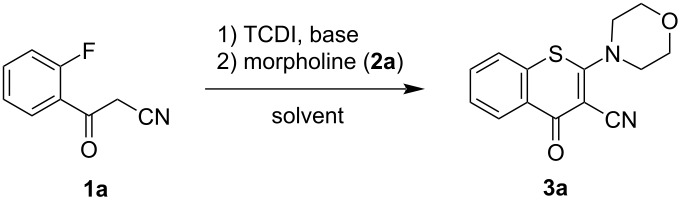					

Entry	Base	Solvent	Time (h)	*T* (°C)	Yield (%)^b^

1	–	DCM	5	40	0
2	Et_3_N	DCM	5	40	trace
3	Et_3_N	THF	5	70	56
4	Et_3_N	MeCN	2	80	56
5	Et_3_N	DMF	1	100	78
6	DIPEA	DMF	1	100	75
7	DBU	DMF	2	100	0
8	*t*-BuOK	DMF	1	100	0
9	K_2_CO_3_	DMF	1	100	37
10^c^	Et_3_N	DMF	1	100	73

^a^Standard conditions: 1) **1a** (1.0 mmol, 1.0 equiv), base (3.0 mmol, 3.0 equiv), TCDI (1.5 mmol, 1.5 equiv), solvent (3 mL), reflux; 2) **2a** (2.0 mmol, 2.0 equiv), rt, 1 h. ^b^Yields refer to isolated compounds after column chromatography. ^c^TCDI (1.3 mmol, 1.3 equiv).

**Scheme 2:**
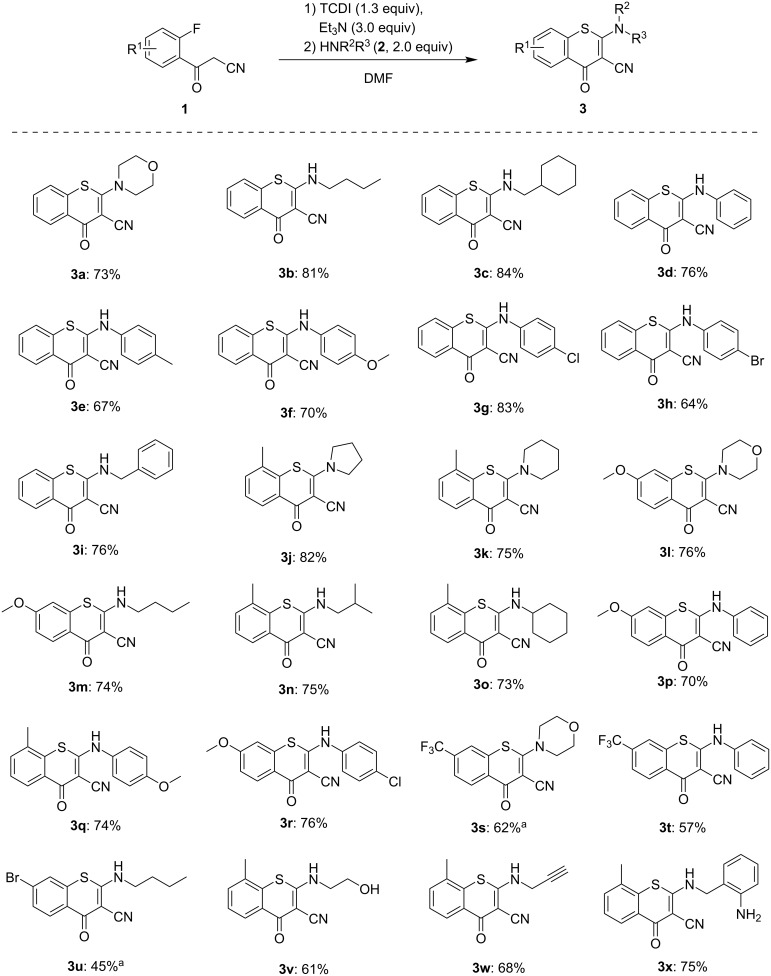
Scope of various amines and substrate **1**. Standard conditions: 1) **1** (1.0 mmol, 1.0 equiv), Et_3_N (3.0 mmol, 3.0 equiv), TCDI (1.3 mmol, 1.3 equiv), DMF (3 mL), 100 °C, 1 h; 2) **2** (2.0 mmol, 2.0 equiv), rt, 1 h. The products were obtained after aqueous washing and filtration. ^a^Yields refer to isolated products after column chromatography.

With the optimal conditions established, the scope of the amine was investigated to diversify the range of substituents for the exploration of different potential bioactivity profiles. Following the initial optimization using the secondary amine morpholine as the substrate, we evaluated the generality of the optimized conditions by testing a range of different amines. Moreover, the workup procedure was simplified, yielding the target product in good purity without the need of column chromatography. As shown in Scheme 2, the reaction demonstrated a broad amine substrate scope, providing the desired products in moderate to high yields. After having examined morpholine which readily afforded the desired product **3a**, next *n*-butylamine and cyclohexylmethylamine were selected to evaluate the compatibility of the conditions with primary amines. The corresponding products **3b** and **3c** were obtained in good yields (81% and 84%, respectively), indicating that the presence of a free N–H bond does not impede the reaction. Then, we explored aromatic amines and investigated the effect of substituents on the aromatic ring. Anilines bearing either electron-donating (4-Me, 4-MeO) or electron-withdrawing groups (4-Cl, 4-Br) effectively afforded the products **3d**–**h** in 64% to 83% yield. The results revealed that the electronic properties of the substituents on the aniline ring have little influence on the yields. In addition, benzylamine was also compatible with the optimized conditions, providing product **3i** in 76% yield. Taken together, it is inferred that the reaction efficiency does not strongly correlate with the nucleophilicity of the amine substrates. Subsequently, the scope of substrate **1** was extended to derivatives bearing various substituents on the benzene ring. Electron-donating groups were well tolerated, affording products **3j**–**r** in good to high yields (70–82%). In contrast, substrates bearing additional electron-withdrawing groups on the benzene ring (**3s**–**u**) generally gave lower yields (45–62%). Moreover, protic and unsaturated moieties, including hydroxy (**3v**, 61%) and alkynyl (**3w**, 68%) groups, were well tolerated under the optimized conditions. Furthermore, the reaction exhibited a preference for the functionalization of aliphatic amines over aromatic ones, as exemplified by the excellent chemoselectivity of product **3x**. The above results demonstrate the broad applicability of the method.

**Scheme 3:**
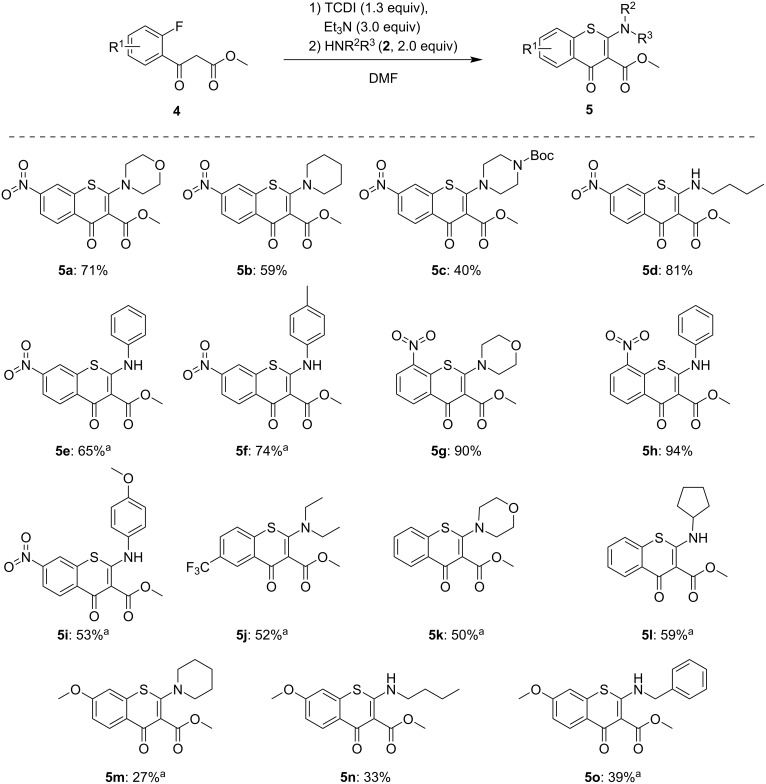
Synthesis of thiochromones from dicarbonyl analogs **4***.* Standard conditions: 1) **4** (1.0 mmol, 1.0 equiv), Et_3_N (3.0 mmol, 3.0 equiv), TCDI (1.3 mmol, 1.3 equiv), DMF (3 mL), 100 °C, 1 h; 2) **2** (2.0 mmol, 2.0 equiv), rt, 1 h; the products were obtained after aqueous washing and filtration. ^a^Yields refer to isolated product after column chromatography.

The reaction is proposed to be initiated by the generation of a carbanion from the activated methylene group. To validate the effect of initial carbanion generation, dicarbonyl analogs **4**, in which the cyano group was replaced by a methyl ester, were selected as substrates. The results indicate that the methylene group of these dicarbonyl compounds is also capable of forming a carbanion under the established conditions. As shown in Scheme 3, the substituted methyl 3-(2-fluorophenyl)-3-oxopropanoates **4** underwent the same reaction process as the cyanide-based compounds **1** to construct the thiochromone scaffolds. Indeed, thiochromones **5a–j** bearing electron-withdrawing groups on the benzene ring were obtained in good to excellent yields (40–94%). In contrast, substrates with unsubstituted or benzene rings bearing electron-donating substituents gave substantially lower yields (**5k–o**, 27–59%). This trend is consistent with an intramolecular nucleophilic aromatic substitution process favored by electron-withdrawing substituents. This limitation, however, could be addressed by exploiting the cyanide group presented in series **3** compounds for further derivatization.

**Scheme 4:**
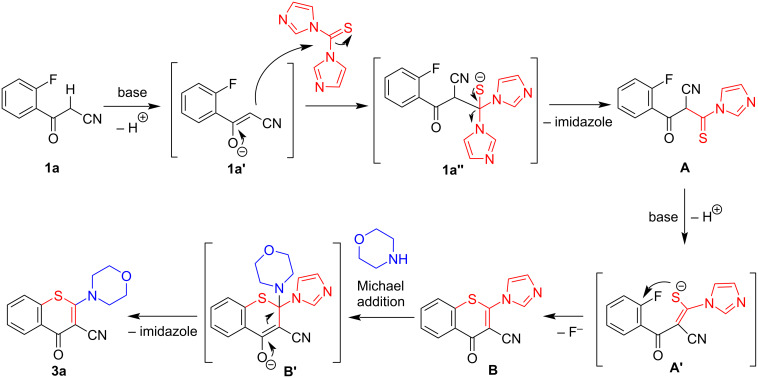
Plausible mechanism.

Based on the experimental results, we propose a plausible mechanism for forming the thiochromone scaffold (Scheme 4). The reaction is initiated by a base-assisted deprotonation of substrate **1a** to generate carbanion **1a’**. Next, an intermolecular nucleophilic attack of carbanion **1a’** on the thiocarbonyl group of TCDI (**1a’’**) with displacing one molecule of imidazole forms intermediate **A**, which was detected by mass spectrometry. However, intermediate **A** is unstable and rapidly cyclizes during work-up or purification. This process proceeds via a second deprotonation to generate a thiolate anion (**A’**), which subsequently engages in an intramolecular nucleophilic aromatic substitution with the fluorine on the benzene ring, to build the cyclized thiochromone core. Finally, the target 2-aminothiochromone **3a** is obtained through a nucleophilic addition–elimination reaction, where the amine adds to intermediate **B**, leading to the expulsion of the second imidazole molecule (**B’**). Notably, the key intermediate **B** was isolated from the reaction mixture, while intermediate **A** was characterized by mass spectrometry during the reaction. Collectively, these results indicate that the initial generation of a carbanion is the key step in this reaction.

**Scheme 5:**
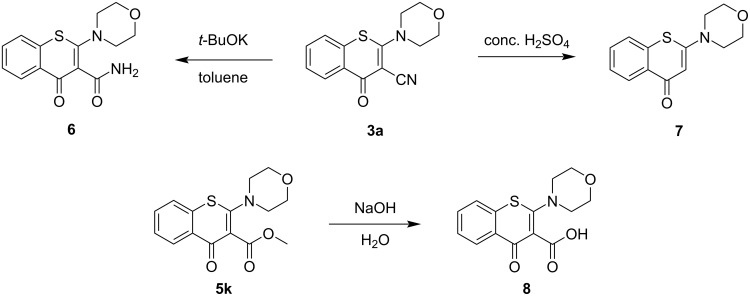
Extended applications.

To evaluate the application prospects of the cascade reaction, the cyano group or methyl ester was functionalized under different conditions (Scheme 5). For instance, hydrolysis of the cyano group in compound **3a** under mild conditions afforded the corresponding amide **6**. Under more violent conditions, such as concentrated sulfuric acid, the cyano group underwent concomitant hydrolysis and decarboxylation to yield compound **7**. Alternatively, basic hydrolysis of the methyl ester in compound **5k** provided the carboxylic acid **8**, which allows for further structural diversification. Together, these transformations highlight the high post-functionalization potential of the present method.

## Conclusion

In summary, we have developed an efficient, cascade synthesis of 2-aminothiochromones from commercially available starting material TCDI. These reactions proceed under mild conditions, tolerate various functional groups, and generally afford the desired products in good to excellent yields. Notably, TCDI has been proved to be uniquely suited for this process as a multifunctional reagent. It serves not only as the sulfur source for thiochromone construction but also as a reactive handle for the subsequent introduction of diverse amine nucleophiles, yielding a variety of 2-aminothiochromones with high efficiency. Furthermore, the presence of the cyano and methyl ester group offers a versatile structural elaboration, thereby providing a flexible platform for the construction of compound libraries with structural diversity. The biological activity and potential applications of these thiochromone derivatives are currently under investigation.

## Experimental

### General information

Chemicals and solvents were purchased from commercial sources and were used as received. TLC was performed on silica gel plates (GF254) with visualization of components by UV light (254 nm). Column chromatography was carried out on silica gel (200–300 mesh). All melting points were measured using a melting point apparatus (MP470, Yanaco), and the temperatures were uncorrected. The structural characterizations of the prepared compounds were performed by ^1^H NMR and ^13^C NMR spectroscopy and high-resolution mass spectrometry (HRMS). ^1^H NMR spectra were obtained on an ECZ-400 spectrometer (JEOL, Tokyo, Japan) at 400 MHz and a Bruker AVANCE 500 (Bruker, Rheinstetten, Germany) at 500 MHz. ^13^C NMR spectra were obtained on a Bruker 400 (Bruker, Rheinstetten, Germany) at 100 MHz and a Bruker AVANCE 500 (Bruker, Rheinstetten, Germany) at 125 MHz. Chemical shift values were referenced to the residual solvent peak and reported in ppm (δ scale) and all coupling constant (*J*) values were given in Hz. CDCl_3_ or DMSO‑*d*_6_ was used as the standard NMR solvent. The following multiplicity abbreviations were used: (s) singlet, (d) doublet, (t) triplet, (q) quartet, (m) multiplet and (brs) broad singlet. HRESIMS data were measured on a Thermo Exactive Orbitrap plus spectrometer. If necessary, the target compounds were purified by chromatography with a purity of >95% as determined by UPLC analysis conducted on a Waters ACQUITY UPLC H-Class and ACQUITY QDa system, using a reversed-phase C18 column (ACQUITY UPLC BEH C18 1.7 μm, 2.1 mm × 100 mm) with a gradient of 5–95% CH_3_CN in water (0.1% HCOOH) in 10 min at a flow rate of 0.3 mL min^−1^.

### General procedure for the synthesis of thiochromones

To a solution of compound **1** or **4** (1.00 mmol, 1.0 equiv) in *N*,*N*-dimethylformamide (3 mL) was added 1,1'-thiocarbonyldiimidazole (1.30 mmol, 1.3 equiv) followed by triethylamine (3.00 mmol, 3.0 equiv). The mixture was heated at 100 °C for 1 h. After cooling to ambient temperature, compound **2** (2.00 mmol, 2.0 equiv) was added and the reaction mixture was stirred for 1 h at room temperature. Upon completion, the mixture was quenched with water (40 mL). The resulting precipitate was isolated by filtration, washed with water (20 mL), and dried under vacuum to yield the title compound in pure form.

## Supporting Information

File 1 Experimental, characterization data and copies of spectra.

## Data Availability

All data that supports the findings of this study is available in the published article and/or the supporting information of this article.
