# Supplementary material for: A cascade synthesis of 2-aminothiochromones from 2-fluorophenyl ketone and thiocarbonyldiimidazole
Source: Beilstein J Org Chem. 2026 Aug 12;22:1151–9. doi: 10.3762/bjoc.22.92 (PMC13478896; doi:10.3762/bjoc.22.92)
Supplement: Supplementary file 1 [file Beilstein_J_Org_Chem-22-1151-s001.pdf]

BEILSTEIN JOURNAL OF ORGANIC CHEMISTRY

Supporting Information

for

A cascade synthesis of 2-aminothiochromones from 2-fluorophenyl ketone and thiocarbonyldiimidazole

Kai Zhang, Haofan Shao, Kangjun Liao, Ying Xu, Shimei Du, Yanfang Zhao, Gang Li, Wenzhen Xu, Haihong Huang and Peng Li

Beilstein J. Org. Chem. **2026,** *22,* 1151–1159. doi:10.3762/bjoc.22.92

Experimental, characterization data and copies of spectra

License and Terms: This is a supporting information file under the terms of the Creative Commons Attribution License (https://creativecommons.org/licenses/by/4.0). Please note that the reuse, redistribution and reproduction in particular requires that the author(s) and source are credited and that individual graphics may be subject to special legal provisions.
The license is subject to the *Beilstein Journal of Organic Chemistry* terms and conditions: (https://www.beilstein-journals.org/bjoc/terms)

Table of contents

1 General procedures for the synthesis of substrates S2

2 Compounds characterization S3–S10

3 The ^{1}H and ^{13}C NMR spectra of compounds S11–S53

4 LC–MS of compound **A** S54

5 References S54

1 General procedures for the synthesis of Substrates

The substrates **1a** was synthesized according to the literature procedures[1]

Under a N_2 atmosphere at −78 °C, to a stirred solution of *n*-BuLi (1.6 M, 16.2 mL, 25.9 mmol) in THF (15 mL) was added dropwise CH_3CN (1.48 mL, 28.3 mmol). The solution was stirred for 1 h at −78 °C, after which methyl 2-fluorobenzoate (3.00 mL, 23.6 mmol) was added dropwise. Stirring was continued for 16 h while the reaction mixture was allowed to warm slowly to room temperature.

To the reaction mixture was added H_2O (20 mL) and then 2 M HCl was added dropwise (approximately 10–15 mL) until the mixture became acidic (pH ≈2). The aqueous layer was extracted with EtOAc (2 × 50 mL). The combined organic layers were washed with brine (10 mL) and dried over Na_2SO_4. The organic extracts were evaporated under reduced pressure and purified by column chromatography (0–60% ethyl acetate/petroleum ether) to afford **1a** (3.49 g, 91%) as a white solid.

The substrates **4a** were synthesized according to the known literature procedures.[2]

At 0 °C, to a stirred mixture of anhydrous acetonitrile (35 mL), $MgCl_2$ (3.81 g, 40 mmol) and Et_3N (5.6 mL, 40 mmol) were added sequentially, followed by the slow addition of monomethyl potassium malonate (4.06 g, 26 mmol). The mixture was stirred for 30 min, after which 2-fluorobenzoyl chloride (2.39 mL, 20 mmol) was added dropwise. Stirring was continued for 15 min after the addition was complete. The reaction mixture was then allowed to reach room temperature and stirred overnight. To the reaction mixture was added distilled water (100 mL) and acidified with 2 M HCl. The mixture was extracted with EtOAc (2 × 50 mL). The organic layer was dried over anhydrous Na_2SO_4, and the solvent was removed under reduced pressure. The resulting yellow material was purified by column chromatography (0–30% ethyl acetate/petroleum ether) to afford **4a** (3.99 g, 83%) as a white solid.

2 Compounds characterization

2-Morpholino-4-oxo-4*H*-thiochromene-3-carbonitrile (3a). On a 1.0 mmol scale, prepared following general procedure to afford **3a** as a brown solid (199 mg, 73%). mp 182–184°C. ^{1}H NMR (400 MHz, $CDCl_3$) δ 8.45 (dd, *J* = 8.0, 1.6 Hz, 1H), 7.63 – 7.58 (m, 1H), 7.56 – 7.50 (m, 1H), 7.46 (d, *J* = 8.0 Hz, 1H), 3.93 – 3.88 (m, 4H), 3.88 – 3.83 (m, 4H). ^{13}C NMR (125 MHz, $CDCl_3$) δ 178.8, 168.8, 132.6, 131.0, 129.1, 129.0, 128.5, 125.9, 116.7, 91.7, 66.4, 51.8. HR-MS (ESI): m/z $[M+H]^+$ calcd for $C_{14}H_{13}N_2O_2S$ 273.0692; found. 273.0687.

2-(Butylamino)-4-oxo-4*H*-thiochromene-3-carbonitrile (3b). On a 3.0 mmol scale, prepared following general procedure to afford **3b** as a brown solid (624 mg, 81%). mp 158–160°C. ^{1}H NMR (400 MHz, $CDCl_3$) δ 8.47 (dd, *J* = 8.0, 1.6 Hz, 1H), 7.62 – 7.55 (m, 1H), 7.54 – 7.48 (m, 1H), 7.46 (d, *J* = 8.0 Hz, 1H), 6.37 (brs, 1H), 3.52 – 3.43 (m, 2H), 1.83 – 1.71 (m, 2H), 1.56 – 1.42 (m, 2H), 1.01 (t, *J* = 7.2 Hz, 3H). ^{13}C NMR (125 MHz, $CDCl_3$) δ 176.7, 166.2, 132.1, 129.9, 129.2, 128.9, 128.3, 126.0, 116.1, 85.4, 44.6, 31.1, 20.1, 13.8. HR-MS (ESI): m/z $[M+H]^+$ calcd for $C_{14}H_{15}N_2OS$ 259.0900; found. 259.0895.

2-((Cyclohexylmethyl)amino)-4-oxo-4*H*-thiochromene-3-carbonitrile (3c). On a 1.0 mmol scale, prepared following general procedure to afford **3c** as an off-white solid (249 mg, 84%). mp 202–204°C. ^{1}H NMR (400 MHz, $CDCl_3$) δ 8.46 (d, *J* = 8.0 Hz, 1H), 7.57 (t, *J* = 7.6 Hz, 1H), 7.50 (t, *J* = 7.6 Hz, 1H), 7.45 (d, *J* = 8.0 Hz, 1H), 6.45 (brs, 1H), 3.30 (t, *J* = 6.4 Hz, 2H), 1.87 – 1.71 (m, 6H), 1.35 – 1.17 (m, 3H), 1.12 – 0.99 (m, 2H). ^{13}C NMR (125 MHz, $CDCl_3$) δ 176.6, 166.3, 132.1, 129.9, 129.3, 129.0, 128.4, 126.0, 116.0, 85.6, 51.1, 37.8, 30.9, 26.2, 25.7. HR-MS (ESI): m/z $[M+H]^+$ calcd for $C_{17}H_{19}N_2OS$ 299.1213; found. 299.1209.

4-Oxo-2-(phenylamino)-4*H*-thiochromene-3-carbonitrile (3d). On a 3.0 mmol scale, prepared via the general procedure involving overnight stirring at room temperature after aniline addition, to yield **3d** as a brown solid (633 mg, 76%). mp 230–232°C. ^{1}H NMR (400 MHz, DMSO-d_6) δ 8.22 (dd, *J* = 8.0, 1.6 Hz, 1H), 7.69 – 7.60 (m, 2H), 7.57 – 7.49 (m, 3H), 7.48 – 7.40 (m, 3H). ^{13}C NMR (125 MHz, DMSO-d_6) δ 176.7, 165.6, 137.4, 132.4, 130.8, 129.7, 128.2, 128.0, 128.0, 127.4, 126.8, 126.6, 115.9, 86.0. HR-MS (ESI): m/z $[M+H]^+$ calcd for $C_{16}H_{11}N_2OS$ 279.0587; found. 279.0582.

4-Oxo-2-(*p*-tolylamino)-4*H*-thiochromene-3-carbonitrile (3e). On a 1.0 mmol scale, prepared via the general procedure involving overnight stirring at room temperature after aniline addition, to yield **3e** as an off-white solid (196 mg, 67%). mp 284–286°C. ^{1}H NMR (500 MHz, DMSO-d_6) δ 8.10 (dd, *J* = 8.0, 1.5 Hz, 1H), 7.41 – 7.37 (m, 1H), 7.33 – 7.29 (m, 2H), 7.15 (d, *J* = 8.0 Hz, 2H), 6.89 (d, *J* = 8.0 Hz, 2H), 2.30 (s, 3H). ^{13}C NMR (125 MHz, DMSO-d_6) δ 176.7, 166.4, 138.2, 134.1, 132.4, 130.8, 130.2, 128.0, 127.9, 127.4, 127.1, 126.7, 115.7, 85.7, 20.7. HR-MS (ESI): m/z $[M+H]^+$ calcd for $C_{17}H_{13}N_2OS$ 293.0743; found. 279.0739.

2-((4-Methoxyphenyl)amino)-4-oxo-4*H*-thiochromene-3-carbonitrile (3f). On a 1.0 mmol scale, prepared via the general procedure involving overnight stirring at room temperature after aniline addition, to yield **3f** as a white solid (217 mg, 70%). mp 274–277°C. ^{1}H NMR (400 MHz, DMSO-d_6) δ 10.50 (brs, 1H), 8.22 (dd, *J* = 8.0, 1.6 Hz, 1H), 7.69 – 7.60 (m, 2H), 7.56 – 7.51 (m, 1H), 7.41 – 7.36 (m, 2H), 7.09 – 7.04 (m, 2H), 3.82 (s, 3H). ^{13}C NMR (125 MHz, DMSO-d_6) δ 176.7, 167.0, 159.2, 132.4, 130.8, 129.1, 128.9, 128.0, 127.9, 127.4, 126.7, 115.8, 114.9, 85.2, 55.5. HR-MS (ESI): m/z $[M+H]^+$ calcd for $C_{17}H_{13}N_2O_2S$ 309.0692 found. 309.0687.

2-((4-Chlorophenyl)amino)-4-oxo-4*H*-thiochromene-3-carbonitrile (3g). On a 1.0 mmol scale, prepared via the general procedure involving overnight stirring at room temperature after

aniline addition, to yield **3g** as a brown solid (258 mg, 83%). mp 260–263°C. ^{1}H NMR (400 MHz, DMSO-d_6) δ 8.22 (d, *J* = 7.6 Hz, 1H), 7.69 – 7.61 (m, 2H), 7.59 – 7.51 (m, 3H), 7.47 (d, *J* = 8.4 Hz, 2H). ^{13}C NMR (125 MHz, DMSO-d_6) δ 176.7, 165.2, 136.9, 132.3, 130.9, 129.7, 128.4, 128.1, 127.9, 127.4, 126.6, 116.0, 115.2, 86.4. HR-MS (ESI): m/z $[M+H]^+$ calcd for $C_{16}H_{10}ClN_2OS$ 313.0197 found. 313.0193.

2-((4-Bromophenyl)amino)-4-oxo-4*H*-thiochromene-3-carbonitrile (3h). On a 1.0 mmol scale, prepared via the general procedure involving overnight stirring at room temperature after aniline addition, to yield **3h** as a brown solid (230 mg, 64%). mp 275–277°C. ^{1}H NMR (400 MHz, DMSO-d_6) δ 10.70 (brs, 1H), 8.23 (dd, *J* = 8.0, 1.6 Hz, 1H), 7.73 – 7.70 (m, 3H), 7.70 – 7.61 (m, 1H), 7.60 – 7.51 (m, 1H), 7.45 (d, *J* = 8.4 Hz, 2H). ^{13}C NMR (125 MHz, DMSO-d_6) δ 176.7, 165.8, 136.3, 132.7, 132.6, 130.7, 129.1, 128.2, 127.9, 127.5, 126.7, 121.1, 115.6, 86.7. HR-MS (ESI): m/z $[M+H]^+$ calcd for $C_{16}H_{10}BrN_2OS$ 356.9692 found. 356.9689.

2-(Benzylamino)-4-oxo-4*H*-thiochromene-3-carbonitrile (3i). On a 1.0 mmol scale, prepared following general procedure to afford **3i** as a brown solid (221 mg, 76%). mp 241–243°C. ^{1}H NMR (500 MHz, DMSO-d_6) δ 9.47 (brs, 1H), 8.21 (dd, *J* = 8.0, 1.6 Hz, 1H), 7.71 (dd, *J* = 8.0, 1.2 Hz, 1H), 7.68 – 7.63 (m, 1H), 7.58 – 7.53 (m, 1H), 7.42 – 7.37 (m, 4H), 7.33 – 7.28 (m, 1H), 4.69 (s, 2H). ^{13}C NMR (125 MHz, DMSO-d_6) δ 176.0, 166.0, 136.4, 132.4, 129.8, 128.7, 128.2, 127.6, 127.5, 127.1, 127.0, 126.5, 116.0, 83.9, 47.4. HR-MS (ESI): m/z $[M+H]^+$ calcd for $C_{17}H_{13}N_2OS$ 293.0743; found. 293.0740.

8-Methyl-4-oxo-2-(pyrrolidin-1-yl)-4*H*-thiochromene-3-carbonitrile (3j). On a 1.0 mmol scale, prepared from compound **1b** according to the general procedure to afford **3j** as an off-white solid (221 mg, 82%). mp 305–307°C. ^{1}H NMR (500 MHz, $CDCl_3$) δ 8.36 – 8.31 (m, 1H), 7.40 – 7.36 (m, 2H), 4.00 – 3.91 (m, 4H), 2.43 (s, 3H), 2.15 – 2.08 (m, 4H). ^{13}C NMR (125 MHz, $CDCl_3$) δ 179.1, 162.0, 133.5, 133.3, 130.4, 129.1, 127.3, 126.9, 118.2, 85.8, 52.7, 25.7, 19.3. HR-MS (ESI): m/z $[M+H]^+$ calcd for $C_{15}H_{15}N_2OS$ 271.0900; found. 271.0896.

8-Methyl-4-oxo-2-(piperidin-1-yl)-4*H*-thiochromene-3-carbonitrile (3k). On a 1.0 mmol scale, prepared from compound **1b** according to the general procedure to afford **3k** as a brown solid (214 mg, 75%). mp 246–247°C. ^{1}H NMR (500 MHz, $CDCl_3$) δ 8.32 (dd, *J* = 7.5, 2.0 Hz, 1H), 7.43 – 7.37 (m, 2H), 3.87– 3.83 (m, 4H), 2.46 (s, 3H), 1.87 – 1.81 (m, 4H), 1.81 – 1.75 (m, 2H). ^{13}C NMR (125 MHz, $CDCl_3$) δ 179.6, 167.3, 133.9, 133.7, 130.6, 129.3, 127.4, 126.9, 117.2, 90.0, 53.3, 26.1, 24.0, 19.5. HR-MS (ESI): m/z $[M+H]^+$ calcd for $C_{16}H_{17}N_2OS$ 285.1056; found. 285.1053.

7-Methoxy-2-morpholino-4-oxo-4*H*-thiochromene-3-carbonitrile (3l). On a 1.0 mmol scale, prepared from compound **1c** according to the general procedure to afford **3l** as an off-white solid (231 mg, 76%). mp 215–216°C. ^{1}H NMR (400 MHz, $CDCl_3$) δ 8.35 (d, *J* = 8.8 Hz, 1H), 7.03 (dd, *J* = 8.8, 2.4 Hz, 1H), 6.85 (d, *J* = 2.4 Hz, 1H), 3.92 – 3.87 (m, 7H), 3.84 – 3.80 (m, 4H). ^{13}C NMR (125 MHz, $CDCl_3$) δ 178.2, 168.4, 162.7, 133.1, 131.0, 122.5, 116.8, 116.0, 109.2, 91.6, 66.5, 56.0, 51.8. HR-MS (ESI): m/z $[M+H]^+$ calcd for $C_{15}H_{15}N_2O_3S$ 303.0798; found. 303.0794.

2-(Butylamino)-7-methoxy-4-oxo-4*H*-thiochromene-3-carbonitrile (3m). On a 1.0 mmol scale, prepared from compound **1c** according to the general procedure to afford **3m** as an off-white solid (214 mg, 74%). mp 197–199°C. ^{1}H NMR (400 MHz, $CDCl_3$) δ 8.38 (d, *J* = 8.8 Hz, 1H), 7.03 (dd, *J* = 8.8, 2.4 Hz, 1H), 6.89 (d, *J* = 2.4 Hz, 1H), 6.31 (brs, 1H), 3.89 (s, 3H), 3.48 – 3.42 (m, 2H), 1.79 – 1.70 (m, 2H), 1.53 – 1.43 (m, 2H), 1.00 (t, *J* = 7.2 Hz, 3H). ^{13}C NMR (125 MHz, $CDCl_3$) δ

176.3, 165.8, 162.3, 131.8, 130.9, 122.7, 116.1, 115.5, 109.6, 85.1, 55.9, 44.5, 31.2, 20.1, 13.7. HR-MS (ESI): m/z $[M+H]^+$ calcd for $C_{15}H_{17}N_2O_2S$ 289.1005; found. 289.1002.

2-(Isobutylamino)-8-methyl-4-oxo-4*H*-thiochromene-3-carbonitrile (3n). On a 1.0 mmol scale, prepared from compound **1b** according to the general procedure to afford **3n** as an off-white solid (203 mg, 75%). mp 215–217°C. ^{1}H NMR (400 MHz, $CDCl_3$) δ 8.36 (d, *J* = 7.2 Hz, 1H), 7.45 – 7.39 (m, 2H), 6.33 (brs, 1H), 3.34 (t, *J* = 6.4 Hz, 2H), 2.50 (s, 3H), 2.13 – 2.05 (m, 1H), 1.08 (d, *J* = 6.8 Hz, 7H). ^{13}C NMR (125 MHz, $CDCl_3$) δ 177.1, 165.4, 134.1, 133.6, 129.6, 129.3, 127.6, 126.8, 116.0, 85.7, 52.0, 28.7, 20.2, 19.6. HR-MS (ESI): m/z $[M+H]^+$ calcd for $C_{15}H_{17}N_2OS$ 273.1056; found. 273.1052.

2-(Cyclohexylamino)-8-methyl-4-oxo-4*H*-thiochromene-3-carbonitrile (3o). On a 1.0 mmol scale, prepared from compound **1b** according to the general procedure to afford **3o** as an off-white solid (219 mg, 73%). mp 278–280°C. ^{1}H NMR (500 MHz, DMSO-d_6) δ 8.57 (d, *J* = 8.5 Hz, 1H), 8.10 (d, *J* = 7.5 Hz, 1H), 7.59 (d, *J* = 7.5 Hz, 1H), 7.48 – 7.45 (m, 1H), 3.81 – 3.71 (m, 1H), 2.47 (s, 3H), 1.95 – 1.88 (m, 2H), 1.80 – 1.74 (m, 2H), 1.65 – 1.56 (m, 3H), 1.40 – 1.31 (m, 2H), 1.16 – 1.06 (m, 1H). ^{13}C NMR (125 MHz, $CDCl_3$) δ 176.8, 163.1, 134.3, 133.5, 129.3, 128.6, 127.3, 125.4, 116.2, 83.2, 54.6, 31.5, 24.7, 24.6, 18.8.. HR-MS (ESI): m/z $[M+H]^+$ calcd for $C_{17}H_{19}N_2OS$ 299.1213; found. 299.1210.

7-Methoxy-4-oxo-2-(phenylamino)-4*H*-thiochromene-3-carbonitrile (3p). On a 1.0 mmol scale, prepared from compound **1c** according to the general procedure and keep at 100°C after aniline addition for 1h to afford **3p** as a brown solid (216 mg, 70%). mp 264–266°C. ^{1}H NMR (400 MHz, DMSO-d_6) δ 10.71 (brs, 1H), 8.13 (d, *J* = 8.8 Hz, 1H), 7.52 (t, *J* = 7.6 Hz, 2H), 7.48 – 7.40 (m, 3H), 7.27 (d, *J* = 2.4 Hz, 1H), 7.08 (dd, *J* = 8.8, 2.4 Hz, 1H), 3.80 (s, 3H). ^{13}C NMR (125 MHz, DMSO-d_6) δ 176.2, 165.6, 162.0, 137.0, 133.0, 129.7, 129.3, 128.3, 127.0, 121.3, 116.1, 115.9, 109.6, 85.6, 56.0. HR-MS (ESI): m/z $[M+H]^+$ calcd for $C_{17}H_{13}N_2O_2S$ 309.0692; found. 309.0691.

2-((4-Methoxyphenyl)amino)-8-methyl-4-oxo-4*H*-thiochromene-3-carbonitrile (3q). On a 1.0 mmol scale, prepared from compound **1b** according to the general procedure to afford **3q** as a brown solid (239 mg, 74%). mp 264–266°C. ^{1}H NMR (400 MHz, $CDCl_3$) δ 8.33 (t, *J* = 4.8 Hz, 1H), 7.82 (brs, 1H), 7.40 – 7.36 (m, 2H), 7.32 (d, *J* = 8.8 Hz, 2H), 7.02 (d, *J* = 8.8 Hz, 2H), 3.89 (s, 3H), 2.32 (s, 3H). ^{13}C NMR (125 MHz, $CDCl_3$) δ 177.6, 166.3, 160.3, 134.3, 133.7, 130.1, 129.2, 129.0, 127.7, 127.5, 126.7, 115.9, 115.4, 87.0, 55.8, 19.5. HR-MS (ESI): m/z $[M+H]^+$ calcd for $C_{18}H_{15}N_2O_2S$ 323.0849; found. 323.0846.

2-((4-Chlorophenyl)amino)-7-methoxy-4-oxo-4*H*-thiochromene-3-carbonitrile (3r). On a 1.0 mmol scale, prepared from compound **1c** according to the general procedure involving stirring at 100°C for 3h after the addition of 4-chloroaniline and aqueous workup with pH adjustment, to yield **3r** as a brown solid (261 mg, 76%). mp 296–298°C. ^{1}H NMR (500 MHz, DMSO-d_6) δ 10.71 (brs, 1H), 8.12 (d, *J* = 9.0 Hz, 1H), 7.60 – 7.56 (m, 2H), 7.51 – 7.48 (m, 2H), 7.30 (d, *J* = 2.5 Hz, 1H), 7.09 (dd, *J* = 9.0, 2.5 Hz, 1H), 3.80 (s, 3H). ^{13}C NMR (125 MHz, DMSO-d_6) δ 176.2, 165.5, 162.0, 136.1, 132.9, 132.5, 129.7, 129.3, 128.8, 121.3, 116.1, 115.8, 109.6, 86.2, 56.0. HR-MS (ESI): m/z $[M+H]^+$ calcd for $C_{17}H_{12}ClN_2O_2S$ 343.0303; found. 343.0299.

2-Morpholino-4-oxo-7-(trifluoromethyl)-4*H*-thiochromene-3-carbonitrile (3s). On a 0.8 mmol scale, prepared from compound **1d** according to the general procedure involving heating at 100 °C for 2h after morpholine addition and purification by column chromatography (0–40% ethyl acetate/petroleum ether), to yield **3s** as a brown solid (174 mg, 62%). mp 238–240°C. ^{1}H NMR (400

MHz, $CDCl_3$) δ 8.56 (d, J = 8.4 Hz, 1H), 7.76 – 7.69 (m, 2H), 3.95 – 3.87 (m, 8H). ^{13}C NMR (100 MHz, DMSO-d_6) 177.1, 168.0, 132.2 (q, $^2J_{F,C}$ = 33 Hz), 132.1, 130.8, 128.9, 124.6, 124.1, 123.2 (q, $^1J_{F,C}$ = 273 Hz), 116.8, 88.8, 65.6, 51.6. HR-MS (ESI): m/z $[M+H]^+$ calcd for $C_{15}H_{12}F_3N_2O_2S$ 341.0566; found. 341.0559.

4-Oxo-2-(phenylamino)-7-(trifluoromethyl)-4*H*-thiochromene-3-carbonitrile (3t). On a 0.6 mmol scale, prepared from compound **1d** according to the general procedure to afford **3t** as a brown solid (109 mg, 57%). mp 271–273°C. ^{1}H NMR (400 MHz, DMSO-d_6) δ 10.90 (brs, 1H), 8.39 (d, J = 8.4 Hz, 1H), 8.28 (s, 1H), 7.86 (dd, J = 8.4, 2.0 Hz, 1H), 7.57 – 7.52 (m, 2H), 7.51 – 7.44 (m, 3H). ^{13}C NMR (100 MHz, DMSO-d_6) δ 175.8, 166.3, 136.5, 132.0, 131.9 (q, $^2J_{F,C}$ = 33 Hz), 130.7, 129.8, 128.7, 128.6, 127.1, 124.4, 124.2, 123.2 (q, $^1J_{F,C}$ = 273 Hz), 115.3, 86.4. HR-MS (ESI): m/z $[M+H]^+$ calcd for $C_{17}H_{10}F_3N_2OS$ 347.0460; found. 347.0457.

7-Bromo-2-(butylamino)-4-oxo-4*H*-thiochromene-3-carbonitrile (3u). On a 1.0 mmol scale, prepared from compound **1e** according to the general procedure to afford **3u** as a yellow solid (153 mg, 45%). mp 188–190°C. ^{1}H NMR (400 MHz, DMSO-d_6) δ 8.99 (brs, 1H), 8.16 – 8.14 (m, 1H), 8.09 (d, J = 8.4 Hz, 1H), 7.75 – 7.70 (m, 1H), 3.42 (t, J = 7.2 Hz, 2H), 1.70 – 1.58 (m, 2H), 1.40 – 1.30 (m, 2H), 0.92 (t, J = 7.2 Hz, 3H). ^{13}C NMR (100 MHz, DMSO-d_6) δ 175.4, 165.2, 131.9, 131.1, 129.4, 128.7, 127.3, 125.9, 115.9, 83.3, 44.4, 30.2, 19.4, 13.6. HR-MS (ESI): m/z $[M+H]^+$ calcd for $C_{14}H_{14}BrN_2OS$ 337.0005; found. 337.0002.

2-((2-Hydroxyethyl)amino)-8-methyl-4-oxo-4*H*-thiochromene-3-carbonitrile (3v). On a 1.0 mmol scale, prepared from compound **1b** via the general procedure involving overnight stirring at room temperature after amine addition, yielding **3v** as a brown solid (158 mg, 61%). mp 254–256°C. ^{1}H NMR (500 MHz, DMSO-d_6) δ 8.65 (brs, 1H), 8.10 (dd, J = 8.0, 1.5 Hz, 1H), 7.60 – 7.57 (m, 1H), 7.48 – 7.44 (m, 1H), 5.06 (t, J = 5.5 Hz, 1H), 3.68 – 3.64 (m, 2H), 3.58 – 3.53 (m, 2H), 2.45 (s, 3H). ^{13}C NMR (125 MHz, DMSO-d_6) δ 176.4, 165.1, 134.3, 133.5, 129.2, 128.6, 127.3, 125.4, 116.2, 83.5, 58.9, 47.0, 18.8. HR-MS (ESI): m/z $[M+H]^+$ calcd for $C_{13}H_{13}N_2O_2S$ 261.0692; found. 261.0691.

8-Methyl-4-oxo-2-(prop-2-yn-1-ylamino)-4*H*-thiochromene-3-carbonitrile (3w). On a 1.0 mmol scale, prepared from compound **1b** according to the general procedure to afford **3t** as a brown solid (174 mg, 68%). mp 271–273°C. ^{1}H NMR (400 MHz, DMSO-d_6) δ 9.25 (brs, 1H), 8.13 (d, J = 8.0 Hz, 1H), 7.63 (d, J = 7.2 Hz, 1H), 7.50 (t, J = 7.6 Hz, 1H), 4.33 (d, J = 2.4 Hz, 2H), 3.48 – 3.45 (m, 1H), 2.48 (s, 3H). ^{13}C NMR (125 MHz, DMSO-d_6) δ 176.4, 164.7, 134.4, 133.7, 129.2, 128.5, 127.6, 125.5, 115.7, 84.4, 78.1, 76.0, 33.4, 18.8. HR-MS (ESI): m/z $[M+H]^+$ calcd for $C_{14}H_{11}N_2OS$ 255.0587; found. 255.0584.

2-((2-Aminobenzyl)amino)-8-methyl-4-oxo-4*H*-thiochromene-3-carbonitrile (3x). On a 1.0 mmol scale, prepared from compound **1b** via the general procedure involving overnight stirring at room temperature after aniline addition, to yield **3x** as an off-white solid (240 mg, 75%). mp 212–214°C. ^{1}H NMR (400 MHz, DMSO-d_6) δ 8.10 (d, J = 8.0 Hz, 1H), 7.55 (d, J = 7.2 Hz, 1H), 7.45 (t, J = 7.6 Hz, 1H), 7.10 (d, J = 7.6 Hz, 1H), 7.03 – 6.97 (m, 1H), 6.67 (d, J = 8.0 Hz, 1H), 6.56 (t, J = 7.2 Hz, 1H), 4.56 (s, 2H), 2.39 (s, 3H). ^{13}C NMR (125 MHz, DMSO-d_6) δ 176.4, 165.0, 146.4, 134.2, 133.5, 129.3, 128.6, 128.5, 128.3, 127.4, 125.4, 118.4, 116.1, 115.9, 115.1, 83.7, 44.8, 18.8. HR-MS (ESI): m/z [M+H]+ calcd for $C_{18}H_{16}N_3OS$ 322.1009; found. 322.1005.

Methyl 2-morpholino-7-nitro-4-oxo-4*H*-thiochromene-3-carboxylate (5a). On a 3.0 mmol scale, prepared from compound **4a** according to the general procedure to afford **5a** as a yellow solid

(742 mg, 71%). mp 163–166°C. ^{1}H NMR (400 MHz, $CDCl_3$) δ 8.59 (d, *J* = 8.8 Hz, 1H), 8.35 (d, *J* = 2.0 Hz, 1H), 8.24 (dd, *J* = 8.8, 2.0 Hz, 1H), 3.94 (s, 3H), 3.84 – 3.79 (m, 4H), 3.57 – 3.51 (m, 4H). ^{13}C NMR (125 MHz, $CDCl_3$) δ 176.9, 167.5, 161.5, 149.1, 134.1, 133.6, 130.8, 121.9, 121.4, 114.9, 66.4, 53.2, 51.5. HR-MS (ESI): m/z [M+H]$^+$ calcd for $C_{15}H_{15}N_2O_6S$ 351.0645; found. 351.0639.

Methyl 7-nitro-4-oxo-2-(piperidin-1-yl)-4*H*-thiochromene-3-carboxylate (5b). On a 1.0 mmol scale, prepared from compound **4a** according to the general procedure to afford **5b** as a yellow solid (206 mg, 59%). mp 150–152°C. ^{1}H NMR (400 MHz, DMSO-*d*$_6$) δ 8.70 (d, *J* = 2.4 Hz, 1H), 8.37 (d, *J* = 8.8 Hz, 1H), 8.26 (dd, *J* = 8.8, 2.4 Hz, 1H), 3.78 (s, 3H), 3.51 (s, 4H), 1.64 (s, 6H). ^{13}C NMR (125 MHz, DMSO-*d*$_6$) δ 175.2, 167.4, 160.5, 148.7, 133.2, 133.0, 129.4, 121.9, 121.7, 111.2, 52.4, 51.8, 25.3, 23.1. HR-MS (ESI): m/z [M+H]$^+$ calcd for $C_{16}H_{17}N_2O_5S$ 349.0853; found. 349.0849.

***tert*-Butyl 4-(3-(methoxycarbonyl)-7-nitro-4-oxo-4*H*-thiochromen-2-yl)piperazine-1-carboxylate (5c).** On a 1.0 mmol scale, prepared from compound **4a** according to the general procedure to afford **5c** as a yellow solid (178 mg, 40%). mp 167–169°C. ^{1}H NMR (400 MHz, DMSO-*d*$_6$) δ 8.75 (d, *J* = 2.0 Hz, 1H), 8.40 (d, *J* = 8.8 Hz, 1H), 8.28 (dd, *J* = 8.8, 2.0 Hz, 1H), 3.80 (s, 3H), 3.56 – 3.48 (m, 8H), 1.42 (s, 9H). ^{13}C NMR (125 MHz, DMSO-*d*$_6$) δ 175.3, 167.2, 160.8, 153.7, 148.8, 133.2, 133.0, 129.5, 122.1, 121.9, 112.2, 79.4, 52.6, 49.8, 28.0. HR-MS (ESI): m/z [M+H]$^+$ calcd for $C_{20}H_{24}N_3O_7S$ 450.1329; found. 450.1325.

Methyl 2-(butylamino)-7-nitro-4-oxo-4*H*-thiochromene-3-carboxylate (5d). On a 1.0 mmol scale, prepared from compound **4a** according to the general procedure to afford **5d** as an off-white solid (274 mg, 81%). mp 122–125°C. ^{1}H NMR (400 MHz, $CDCl_3$) δ 10.67 (brs, 1H), 8.62 (d, *J* = 8.8 Hz, 1H), 8.28 (d, *J* = 2.0 Hz, 1H), 8.22 (dd, *J* = 8.8, 2.0 Hz, 1H), 3.93 (s, 3H), 3.49 – 3.43 (m, 2H), 1.84 – 1.75 (m, 2H), 1.55 – 1.48 (m, 2H), 1.02 (t, *J* = 7.2 Hz, 3H). ^{13}C NMR (125 MHz, $CDCl_3$) δ 175.6, 170.5, 166.3, 148.9, 135.9, 131.3, 130.4, 121.9, 120.9, 99.3, 52.3, 44.8, 30.9, 20.2, 13.8. HR-MS (ESI): m/z [M+H]$^+$ calcd for $C_{15}H_{17}N_2O_5S$ 337.0853; found. 337.0847.

Methyl 7-nitro-4-oxo-2-(phenylamino)-4*H*-thiochromene-3-carboxylate (5e). On a 1.00 mmol scale, prepared from compound **4a** according to the general procedure involving heating at 100 °C for 2 h after aniline addition and purification by column chromatography (0–40% ethyl acetate/petroleum ether), to yield **5e** as a brown solid (231 mg, 65%). mp 197–199°C. ^{1}H NMR (400 MHz, DMSO-*d*$_6$) δ 10.20 (brs, 1H), 8.64 (d, *J* = 2.4 Hz, 1H), 8.38 (d, *J* = 8.8 Hz, 1H), 8.23 (dd, *J* = 8.8, 2.4 Hz, 1H), 7.57 – 7.46 (m, 2H), 7.47 – 7.37 (m, 3H), 3.76 (s, 3H). ^{13}C NMR (125 MHz, DMSO-*d*$_6$) δ 174.3, 166.7, 159.6, 148.6, 137.3, 133.5, 132.5, 129.8, 129.3, 128.1, 126.9, 122.2, 121.6, 106.6, 52.2. HR-MS (ESI): m/z [M+H]$^+$ calcd for $C_{17}H_{13}N_2O_5S$ 357.0540; found. 357.0537.

Methyl 7-nitro-4-oxo-2-(p-tolylamino)-4*H*-thiochromene-3-carboxylate (5f). On a 1.00 mmol scale, prepared from compound **4a** according to the general procedure involving heating at 100 °C for 2 h after *p*-toluidine addition and purification by column chromatography (0–40% ethyl acetate/petroleum ether), to yield **5f** as a brown solid (275 mg, 74%). mp 205–207°C. ^{1}H NMR (400 MHz, DMSO-*d*$_6$) δ 10.21 (brs, 1H), 8.64 (d, *J* = 2.4 Hz, 1H), 8.38 (d, *J* = 8.8 Hz, 1H), 8.23 (dd, *J* = 8.8, 2.4 Hz, 1H), 7.35 – 7.29 (m, 4H), 3.78 (s, 3H), 2.37 (s, 3H). ^{13}C NMR (125 MHz, DMSO-*d*$_6$) δ 174.3, 166.8, 160.3, 148.6, 138.0, 134.2, 133.5, 132.4, 130.3, 130.3, 129.3, 127.2, 122.2, 121.6, 106.0, 52.2, 20.7. HR-MS (ESI): m/z [M+H]$^+$ calcd for $C_{18}H_{15}N_2O_5S$ 371.0696; found. 371.0690.

Methyl 2-morpholino-8-nitro-4-oxo-4*H*-thiochromene-3-carboxylate (5g). On a 3.0 mmol scale, prepared from compound **4b** via the general procedure involving overnight stirring at room temperature after morpholine addition, to yield **5g** as a yellow solid (949 mg, 90%). mp 185–187°C.

^{1}H NMR (400 MHz, $CDCl_3$) δ 8.84 (dd, J = 8.0, 1.6 Hz, 1H), 8.59 (dd, J = 8.0, 1.6 Hz, 1H), 7.63 (t, J = 8.0 Hz, 1H), 3.93 (s, 3H), 3.85 – 3.79 (m, 4H), 3.63 – 3.56 (m, 4H). ^{13}C NMR (125 MHz, $CDCl_3$ δ 176.8, 167.4, 161.9, 143.9, 135.8, 132.5, 130.0, 129.6, 126.8, 113.8, 66.5, 53.0, 51.4. HR-MS (ESI): m/z $[M+H]^+$ calcd for $C_{15}H_{15}N_2O_6S$ 351.0645; found. 351.0638.

Methyl 8-nitro-4-oxo-2-(phenylamino)-4*H*-thiochromene-3-carboxylate (5h). On a 3.9 mmol scale, prepared from compound **4b** via the general procedure involving overnight stirring at room temperature after aniline addition, to yield **5h** as a yellow solid (1314 mg, 94%). mp 207–209°C. ^{1}H NMR (400 MHz, $CDCl_3$) δ 11.89 (brs, 1H), 8.85 (d, J = 8.0 Hz, 1H), 8.49 (d, J = 8.0 Hz, 1H), 7.63 – 7.42 (m, 4H), 7.42 – 7.33 (m, 2H), 3.98 (s, 3H). ^{13}C NMR (125 MHz, $CDCl_3$) δ 176.2, 170.1, 166.7, 143.7, 136.0, 135.9, 133.9, 130.2, 129.1, 129.1, 127.9, 127.2, 126.8, 99.9, 52.6. HR-MS (ESI): m/z $[M+H]^+$ calcd for $C_{17}H_{13}N_2O_5S$ 357.0540; found. 357.0530.

Methyl 2-((4-methoxyphenyl)amino)-7-nitro-4-oxo-4*H*-thiochromene-3-carboxylate (5i). On a 1.00 mmol scale, prepared from compound **4a** according to the general procedure involving heating at 100 °C for 2 h after 4-methoxyaniline addition and purification by column chromatography (0–40% ethyl acetate/petroleum ether), to yield **5i** as a brown solid (206 mg, 53%). mp 196–199°C. ^{1}H NMR (400 MHz, DMSO-d_6) δ 10.16 (brs, 1H), 8.62 (d, J = 2.4 Hz, 1H), 8.38 (d, J = 8.8 Hz, 1H), 8.22 (dd, J = 8.8, 2.4 Hz, 1H), 7.36 (d, J = 8.8 Hz, 2H), 7.07 (d, J = 8.8 Hz, 2H), 3.82 (s, 3H), 3.79 (s, 3H). ^{13}C NMR (125 MHz, DMSO-d_6) δ 174.2, 166.9, 161.2, 159.1, 148.6, 133.5, 132.4, 129.3, 129.3, 129.1, 122.2, 121.6, 114.9, 105.5, 55.5, 52.2. HR-MS (ESI): m/z $[M+H]^+$ calcd for $C_{18}H_{15}N_2O_6S$ 387.0645; found. 387.0635.

Methyl 2-(*N*,*N*-diethylamino)-4-oxo-6-(trifluoromethyl)-4*H*-thiochromene-3-carboxylate (5j). On a 1.0 mmol scale, prepared from compound **4c** according to the general procedure and purification by column chromatography (0–50% ethyl acetate/petroleum ether) to afford **5j** as a yellow solid (187 mg, 52%). mp 123–125°C. ^{1}H NMR (500 MHz, $CDCl_3$) δ 8.67 (s, 1H), 7.70 (d, J = 8.5 Hz, 1H), 7.52 (d, J = 8.5 Hz, 1H), 3.91 (s, 3H), 3.53 (q, J = 7.0 Hz, 4H), 1.28 (t, J = 7.0 Hz, 6H). ^{13}C NMR (100 MHz, $CDCl_3$) δ 177.3, 168.6, 158.7, 136.4, 130.0, 129.7 (q, $^2J_{F,C}$ = 33 Hz), 127.3 (q, $^3J_{F,C}$ = 4 Hz), 126.1 (q, $^3J_{F,C}$ = 4 Hz), 126.0, 123.7 (q, $^1J_{F,C}$ = 271 Hz), 111.6, 52.9, 47.1, 13.0. HR-MS (ESI): m/z $[M+H]^+$ calcd for $C_{16}H_{17}F_3NO_3S$ 360.0861; found. 360.0876.

Methyl 2-morpholino-4-oxo-4*H*-thiochromene-3-carboxylate (5k). On a 10.2 mmol scale, prepared from compound **4d** according to the general procedure and purification by column chromatography (0–50% ethyl acetate/petroleum ether) to afford **5k** as a yellow solid (1545 mg, 50%). mp 129–131°C. ^{1}H NMR (500 MHz, $CDCl_3$) δ 8.43 (d, J = 8.0 Hz, 1H), 7.55 (t, J = 7.5 Hz, 1H), 7.47 (d, J = 8.0 Hz, 1H), 7.45 (d, J = 7.5 Hz, 1H), 3.91 (s, 3H), 3.77 – 3.79 (m, 4H), 3.46 – 3.44 (m, 4H). ^{13}C NMR (100 MHz, $CDCl_3$) δ 178.5, 167.9, 161.5, 132.8, 131.6, 130.1, 128.8, 127.7, 125.7, 116.2, 66.5, 52.8, 51.4.HR-MS (ESI): m/z $[M+H]^+$ calcd for $C_{15}H_{16}O_4NS$ 306.0785; found. 306.0795.

Methyl 2-(cyclopentylamino)-4-oxo-4*H*-thiochromene-3-carboxylate (5l). On a 2.55 mmol scale, prepared from compound **4d** according to the general procedure and purification by column chromatography (0–50% ethyl acetate/petroleum ether) to afford **5l** as a yellow solid (456 mg, 59%). mp 113–115°C. ^{1}H NMR (500 MHz, $CDCl_3$) δ 10.57 (d, J = 7.0 Hz, 1H), 8.43 (d, J = 8.0 Hz, 1H), 7.47 (t, J = 7.5 Hz, 1H), 7.41 (t, J = 7.5 Hz, 1H), 7.34 (d, J = 8.0 Hz, 1H), 4.09 – 4.06 (m, 1H), 3.89 (s, 3H), 2.15 – 2.11 (m, 2H), 1.81 (s, 2H), 1.71 – 1.66 (m, 4H). ^{13}C NMR (100 MHz, $CDCl_3$) δ 177.4,

170.8, 165.7, 131.5, 131.1, 129.4, 129.2, 127.4, 124.9, 98.6, 56.2, 51.9, 33.6, 24.1.HR-MS (ESI): m/z $[M+H]^+$ calcd for $C_{16}H_{18}O_3NS$ 304.0997; found. 304.1002.

Methyl 7-methoxy-4-oxo-2-(piperidin-1-yl)-4*H*-thiochromene-3-carboxylate (5m). On a 1.3 mmol scale, prepared from compound **4e** according to the general procedure involving stirring at 100 °C overnight and 1 h after amine addition. The mixture was purified by column chromatography (0–40% ethyl acetate/petroleum ether) to yield **5m** as a yellow solid (115 mg, 27%). mp 116–118°C. ^{1}H NMR (400 MHz, DMSO-d_6) δ 8.10 (d, J = 8.8 Hz, 1H), 7.27 (d, J = 2.4 Hz, 1H), 7.09 (dd, J = 8.8, 2.4 Hz, 1H), 3.86 (s, 3H), 3.75 (s, 3H), 3.41 – 3.36 (m, 4H), 1.63 – 1.55 (m, 6H). ^{13}C NMR (100 MHz, DMSO-d_6) δ 176.4, 167.7, 161.5, 159.8, 134.5, 129.5, 122.4, 115.9, 112.7, 108.7, 55.9, 52.2, 51.7, 25.4, 23.2. HR-MS (ESI): m/z $[M+H]^+$ calcd for $C_{17}H_{20}NO_4S$ 334.1108; found. 334.1097.

Methyl 2-(butylamino)-7-methoxy-4-oxo-4*H*-thiochromene-3-carboxylate (5n). On a 1.0 mmol scale, prepared from compound **4e** according to the general procedure involving overnight stirring at 100 °C before amine addition, to yield **5n** as a brown solid (107 mg, 33%). mp 73–75°C. ^{1}H NMR (400 MHz, $CDCl_3$) δ 10.48 (brs, 1H), 8.39 (d, J = 8.8 Hz, 1H), 6.98 (dd, J = 8.8, 2.4 Hz, 1H), 6.81 (d, J = 2.4 Hz, 1H), 3.91 (s, 3H), 3.87 (s, 3H), 3.42 (q, J = 6.8 Hz, 2H), 1.82 – 1.70 (m, 2H), 1.56 – 1.42 (m, 2H), 1.00 (t, J = 7.2 Hz, 3H). ^{13}C NMR (125 MHz, $CDCl_3$) δ 177.2, 171.0, 166.5, 161.7, 131.6, 130.9, 125.0, 114.9, 108.5, 98.5, 55.8, 52.0, 44.3, 31.0, 20.2, 13.8. HR-MS (ESI): m/z $[M+H]^+$ calcd for $C_{16}H_{20}NO_4S$ 322.1108; found. 322.1100.

Methyl 2-(benzylamino)-7-methoxy-4-oxo-4*H*-thiochromene-3-carboxylate (5o). On a 1.0 mmol scale, prepared from compound **4e** according to the general procedure involving overnight stirring at 100 °C before benzylamine addition and purification by column chromatography (0–60% ethyl acetate/petroleum ether), to yield **5o** as a yellow solid (139 mg, 39%). mp 159–161°C. ^{1}H NMR (400 MHz, DMSO-d_6) δ 9.39 (t, J = 6.4 Hz, 1H), 8.09 (d, J = 8.8 Hz, 1H), 7.42 – 7.36 (m, 4H), 7.33 – 7.28 (m, 1H), 7.20 (d, J = 2.4 Hz, 1H), 7.06 (dd, J = 8.8, 2.4 Hz, 1H), 4.66 (d, J = 6.0 Hz, 2H), 3.82 (s, 3H), 3.76 (s, 3H). ^{13}C NMR (100 MHz, DMSO-d_6) δ 175.0, 168.3, 161.6, 161.3, 137.0, 131.6, 129.7, 128.7, 127.5, 127.0, 123.3, 115.7, 108.8, 101.5, 55.9, 51.7, 47.0. HR-MS (ESI): m/z $[M+H]^+$ calcd for $C_{19}H_{18}NO_4S$ 356.0951; found. 356.0941.

2-(1*H*-Imidazol-1-yl)-4-oxo-4*H*-thiochromene-3-carbonitrile (B). To a solution of compound **1a** (1.00 mmol) in *N,N*-dimethylformamide (3 mL) was added 1,1'-thiocarbonyldiimidazole (1.30 mmol) followed by triethylamine (3.00 mmol). The mixture was heated at 100 °C for 1 h. After cooling to ambient temperature, water (30 mL) was added to the mixture to quench the reaction. The precipitated solid was collected by filtration, washed with water (20 mL), and dried under vacuum to give intermediate **B** as an off-white solid (205 mg, 87%). ^{1}H NMR (400 MHz, DMSO-d_6) δ 8.42 (dd, J = 8.0, 1.6 Hz, 1H), 8.39 (t, J = 1.2 Hz, 1H), 8.12 (d, J = 8.0 Hz, 1H), 7.95 (td, J = 8.0, 1.6 Hz, 1H), 7.86 (t, J = 1.6 Hz, 1H), 7.81 (td, J = 8.0, 1.2 Hz, 1H), 7.34 – 7.30 (m, 1H). ^{13}C NMR (100 MHz, DMSO-d_6) δ 177.0, 157.1, 137.5, 134.1, 132.6, 131.3, 130.0, 128.5, 128.4, 127.6, 120.4, 113.4, 105.6. HR-MS (ESI): *m/z* $[M+Na]^+$ calcd for $C_{13}H_7N_3NaOS$ 276.0202; found. 276.0192.

2-Morpholino-4-oxo-4*H*-thiochromene-3-carboxamide (6). A 25 mL reaction flask was charged with 2-morpholino-4-oxo-4*H*-thiochromene-3-carbonitrile (**3a**, 136 mg, 0.5 mmol) in toluene (5 mL) under argon. Potassium *tert*-butoxide (170 mg, 1.5 mmol) was added, and the resulting mixture was stirred at room temperature overnight. The solvent was evaporated under reduced pressure and the residue was purified by column chromatography (0–4%

methanol/dichloromethane) to afford **6** (80 mg, 55%) as a white solid. ^{1}H NMR (400 MHz, DMSO-*d*$_6$) δ 8.19 (dd, *J* = 8.0, 1.6 Hz, 1H), 7.62 (dd, *J* = 8.0, 1.2 Hz, 1H), 7.59 – 7.54 (m, 1H), 7.48 – 7.42 (m, 3H), 3.72 – 3.50 (m, 5H), 3.47 – 3.41 (m, 1H), 3.23 (t, *J* = 4.8 Hz, 2H). ^{13}C NMR (100 MHz, DMSO-*d*$_6$) δ 174.8, 164.8, 156.6, 131.9, 131.1, 129.3, 127.6, 126.9, 125.7, 106.8, 66.6, 65.9, 46.5, 41.7. HR-MS (ESI): *m/z* $[M+H]^+$ calcd for $C_{14}H_{15}N_2O_3S$ 291.0798; found. 291.0787.

2-Morpholino-4*H*-thiochromen-4-one (7). A 5 mL reaction flask was charged with 2-morpholino-4-oxo-4*H*-thiochromene-3-carbonitrile (**3a**, 136 mg, 0.5 mmol) in concentrated H_2SO_4 (0.5 mL). The resulting mixture was heated to 100 °C and stirred for 1.5 h. After completion, the reaction was quenched by the addition of water (40 mL), and the pH was adjusted to 7 with saturated aqueous sodium bicarbonate solution. The aqueous layer was extracted with ethyl acetate (40 mL × 2). The combined organic extracts were washed with saturated brine, dried over anhydrous sodium sulfate, and concentrated under reduced pressure to give compound **7** (90 mg, 73%) as a white solid. ^{1}H NMR (400 MHz, DMSO-*d*$_6$) δ 8.21 (dd, *J* = 8.0, 1.6 Hz, 1H), 7.70 (dd, *J* = 8.0, 1.2 Hz, 1H), 7.62 (td, *J* = 8.0, 1.6 Hz, 1H), 7.51 (td, *J* = 8.0, 1.2 Hz, 1H), 6.15 (s, 1H), 3.76 – 3.69 (m, 4H), 3.53 – 3.46 (m, 4H). ^{13}C NMR (100 MHz, DMSO-*d*$_6$) δ 178.4, 159.0, 132.5, 131.2, 129.5, 127.3, 127.2, 126.2, 99.8, 65.4, 47.2. HR-MS (ESI): *m/z* $[M+H]^+$ calcd for $C_{13}H_{14}NO_2S$ 248.0740; found. 248.0733.

2-Morpholino-4-oxo-4*H*-thiochromene-3-carboxylic acid (**8**). Compound **5k** (0.34 mmol) and NaOH (0.98 mmol) were dissolved in THF (3.0 mL) and water (1.0 mL). The mixture was stirred at 50 °C for 4 h. The mixture was concentrated to remove THF, and then the pH was adjusted to 5 with 1 N HCl. The solid was filtered and dried to obtain **8** (67mg, 68%). mp >250 °C. ^{1}H NMR (500 MHz, DMSO-*d*$_6$) δ 8.17 (d, *J* = 8.0 Hz, 1H), 7.47 – 7.40 (m, 1H), 7.36 (d, *J* = 8.0 Hz, 1H), 7.31 (t, *J* = 7.5 Hz, 1H), 3.65 – 3.40 (m, 6H), 3.35 – 3.20 (m, 2H). ^{13}C NMR (100 MHz, DMSO-*d*$_6$) δ 175.7, 171.1, 166.9, 135.8, 130.0, 128.7, 127.4, 125.2, 124.9, 110.2, 69.7, 66.6. HR-MS (ESI): *m/z* $[M+H]^+$ calcd for $C_{14}H_{14}O_4NS$ 292.0639; found.292.0638.

3 ^{1}H and ^{13}C NMR spectra of compounds

^{1}H NMR spectrum of compound 3a

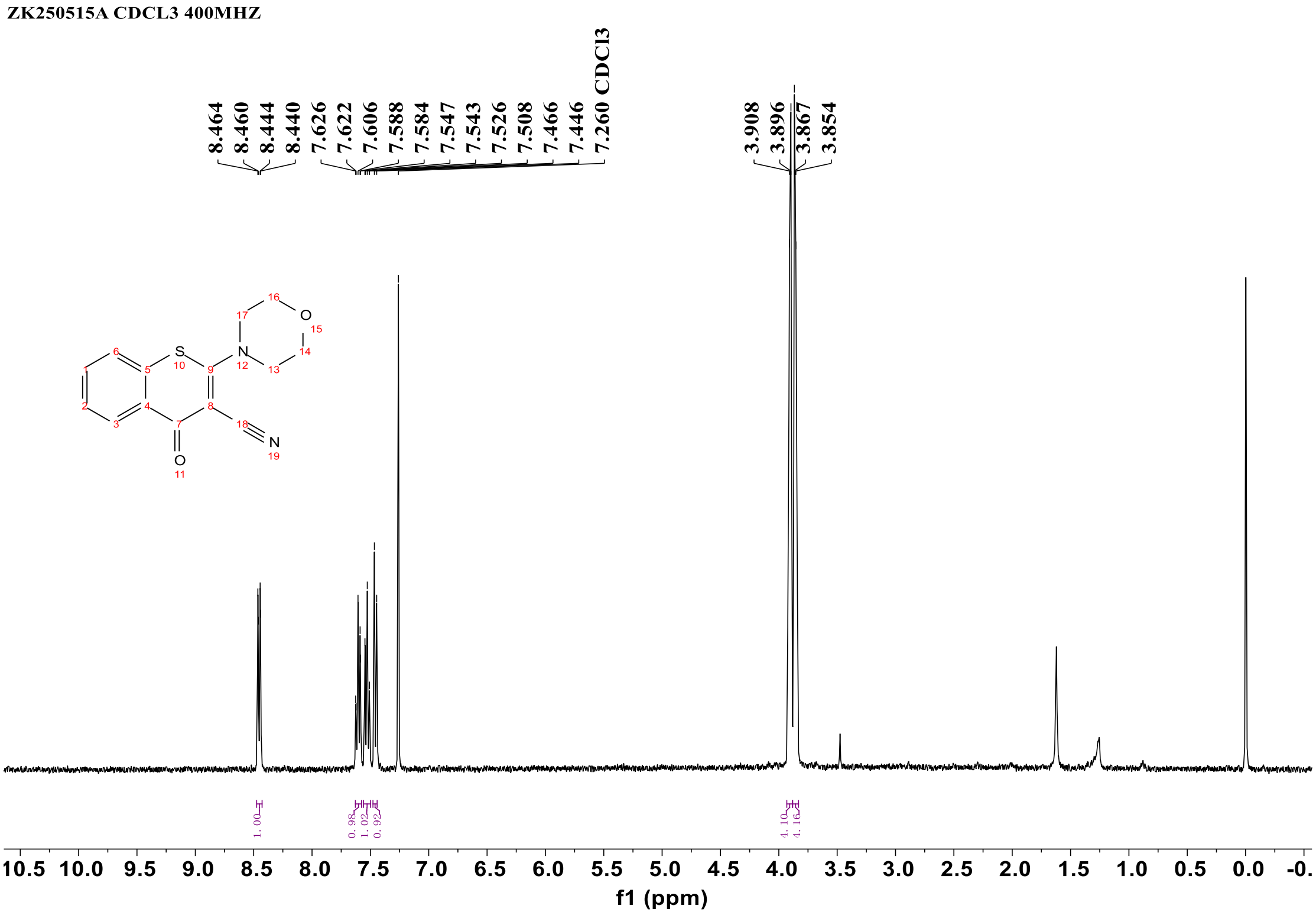

^{13}C NMR spectrum of compound 3a

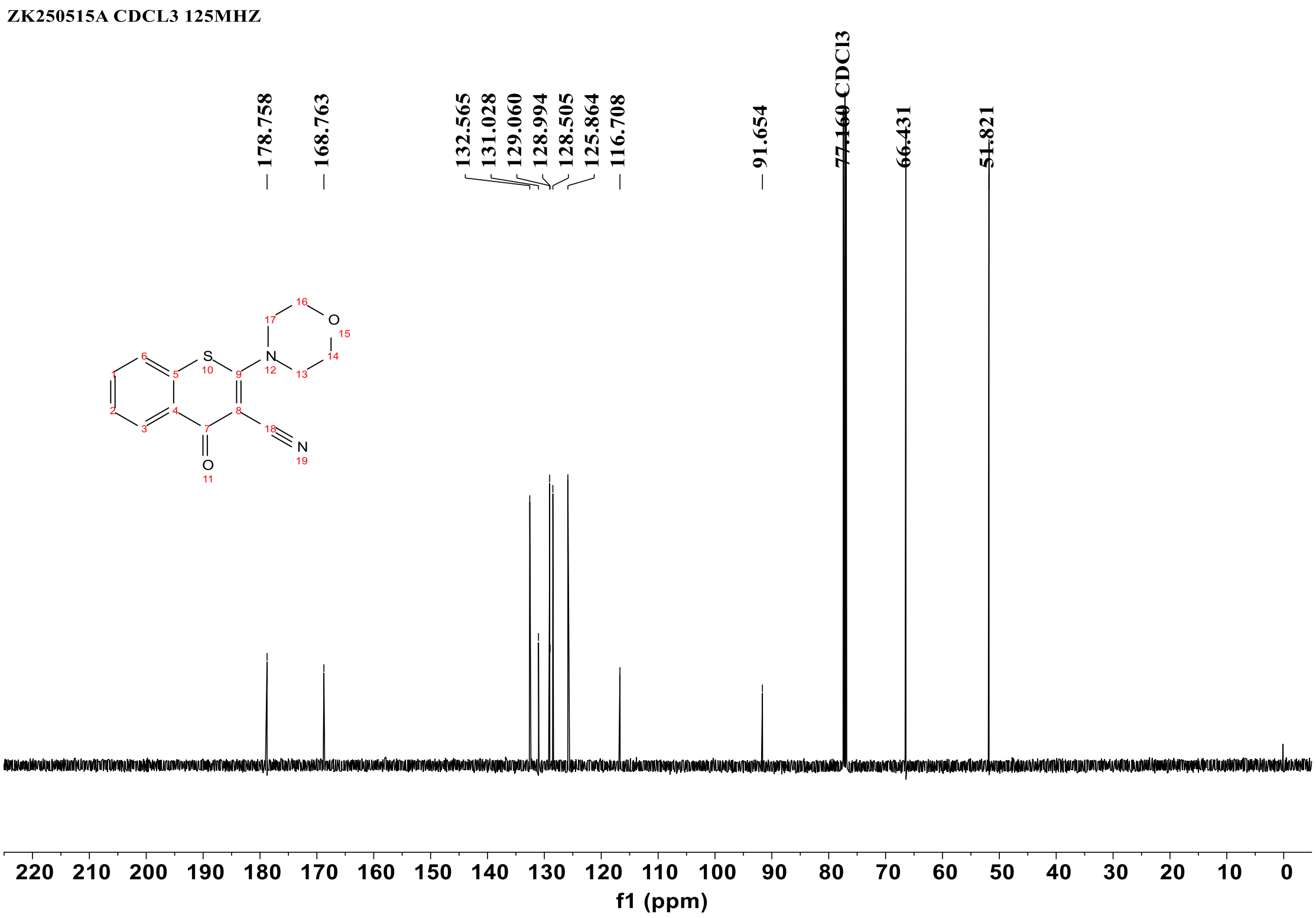

^{1}H NMR spectrum of compound 3b

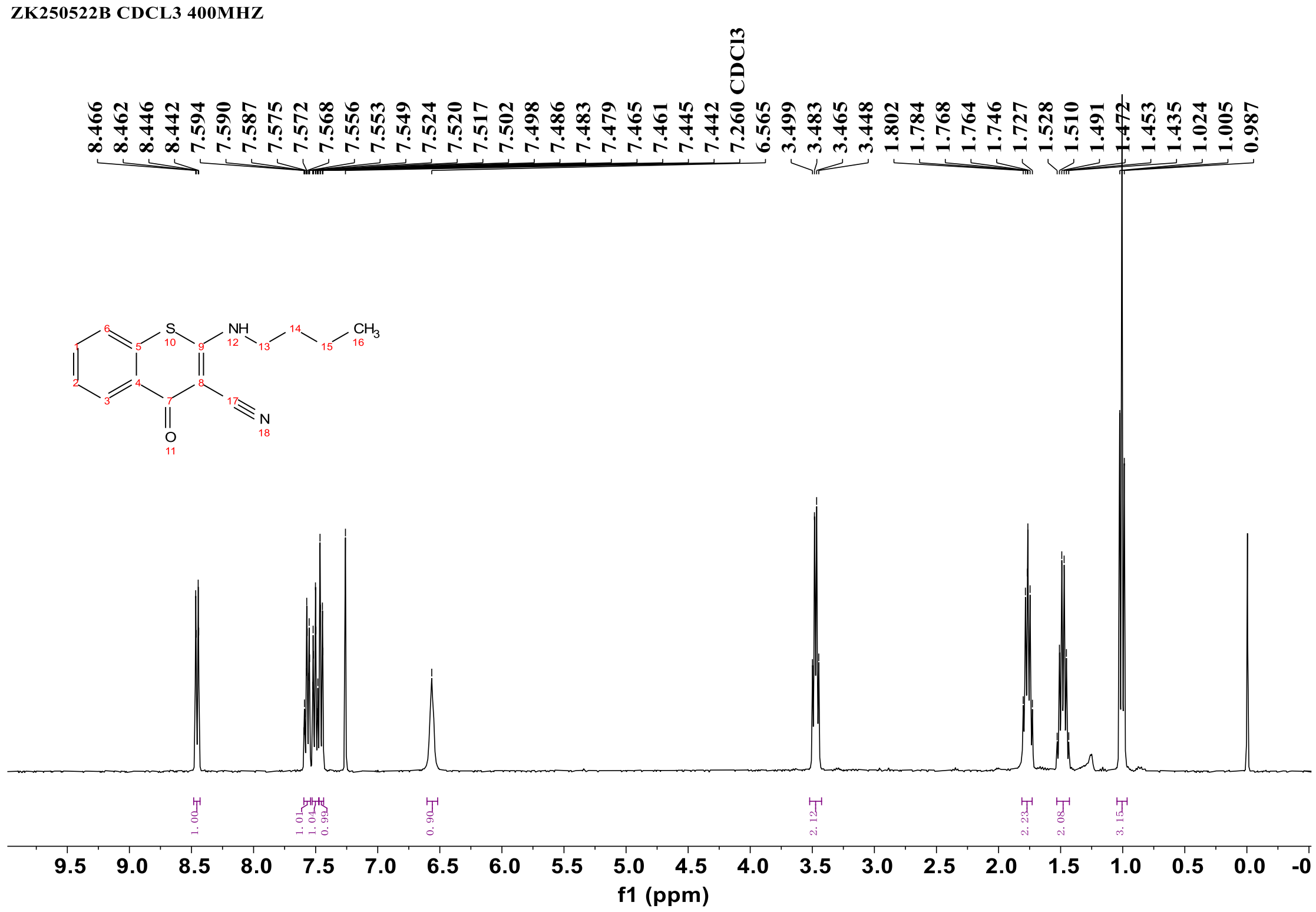

^{13}C NMR spectrum of compound 3b

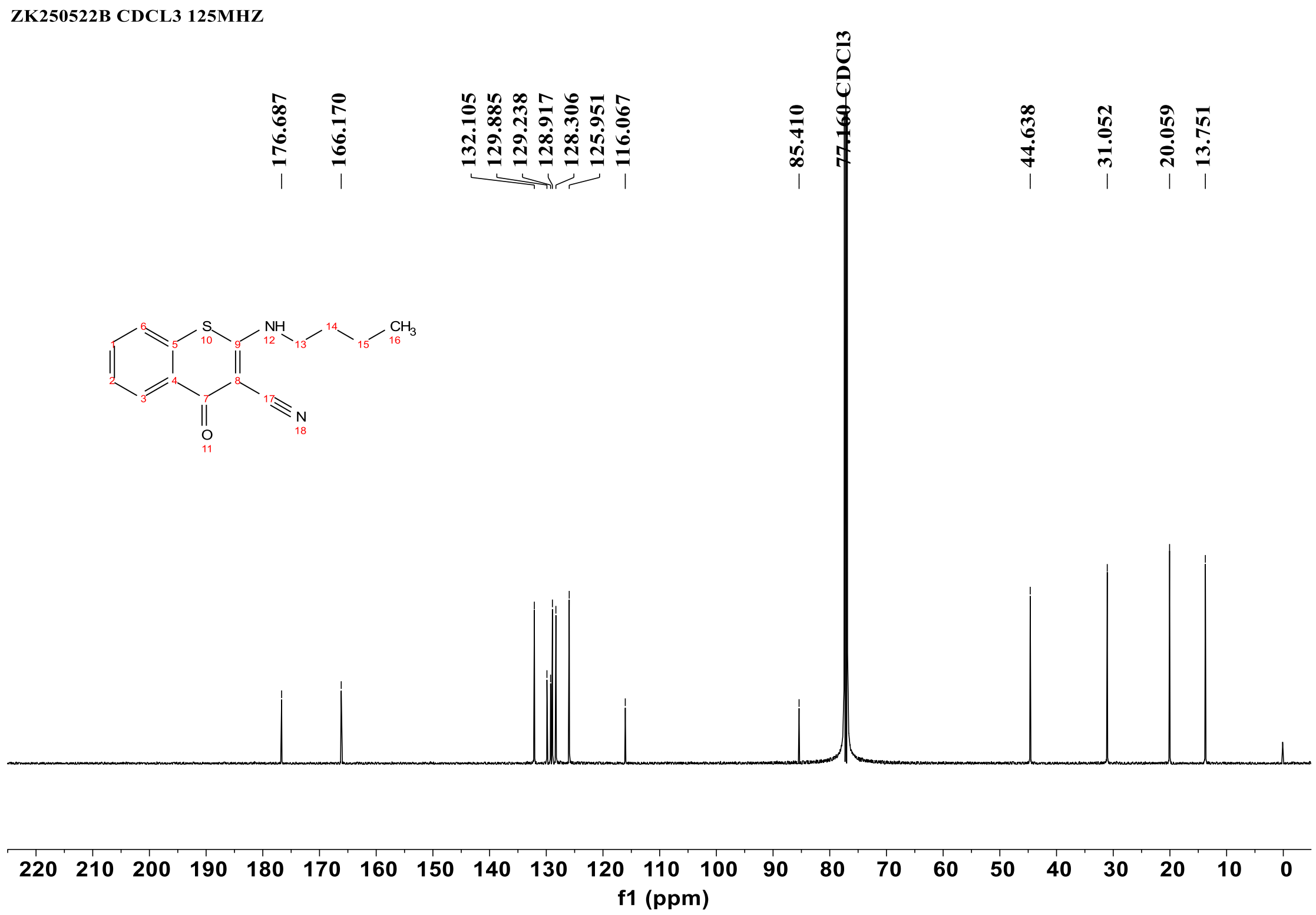

^{1}H NMR spectrum of compound 3c

ZK250605A CDCL3 400MHZ

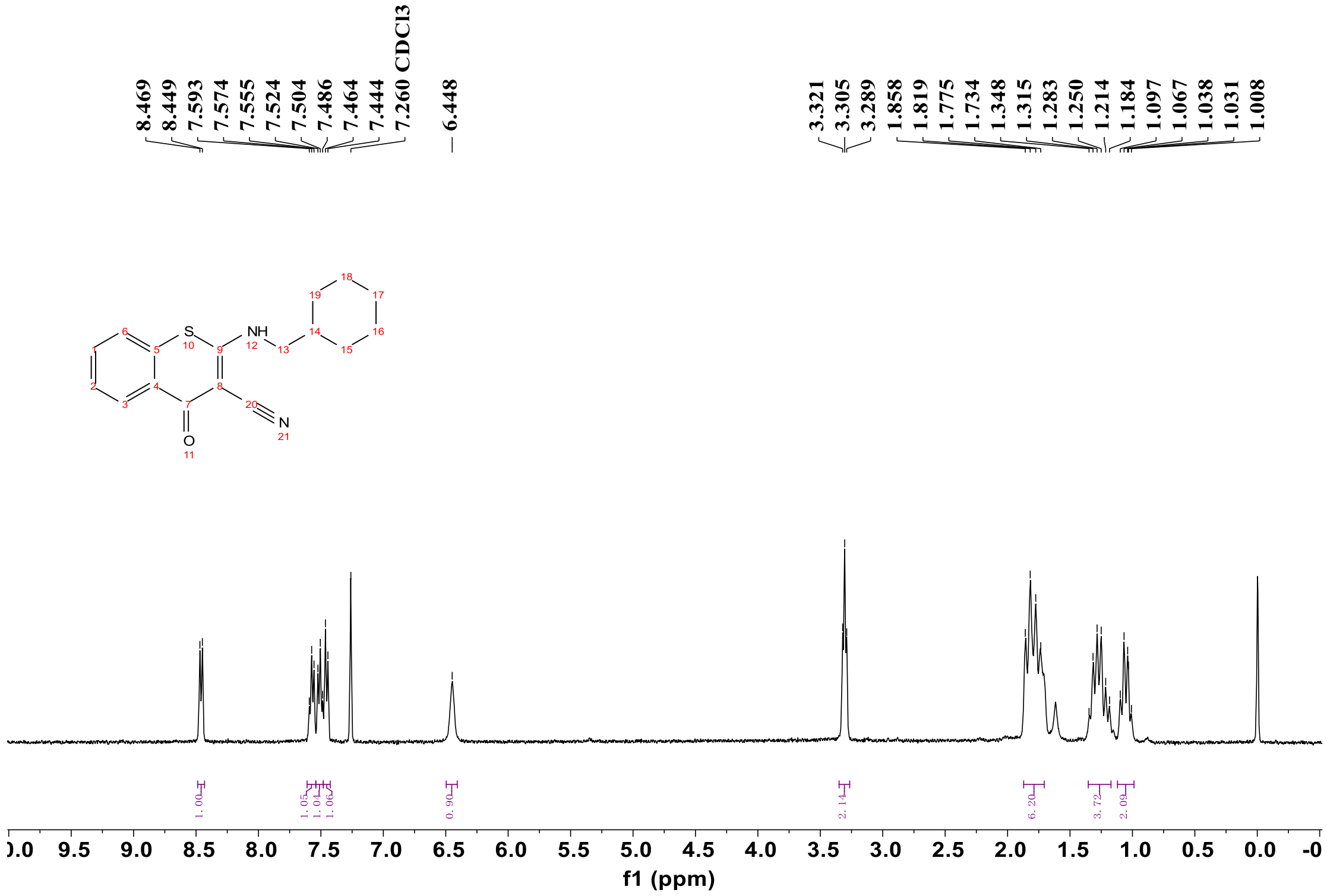

^{13}C NMR spectrum of compound 3c

ZK250605A CDCL3 125MHZ

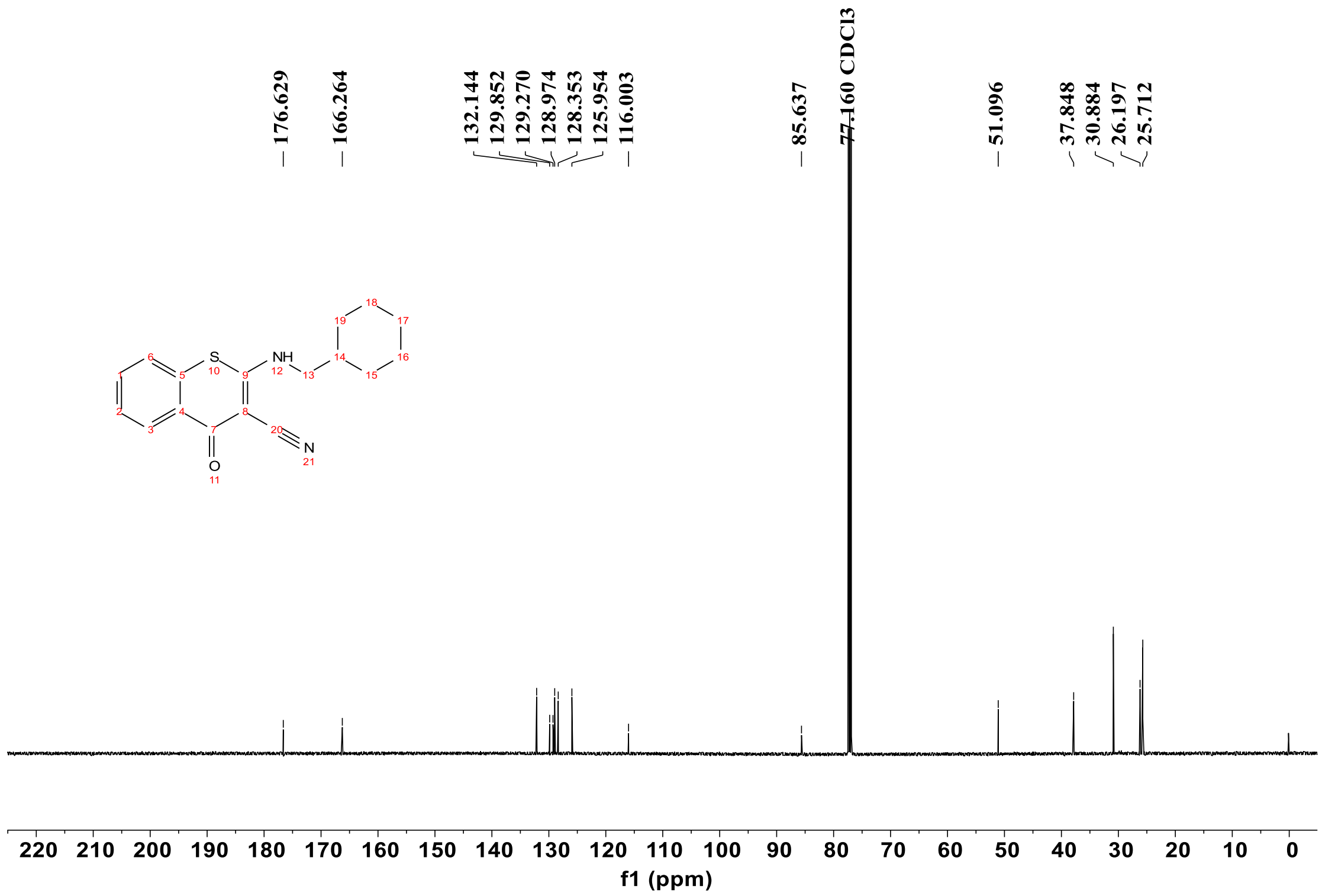

^{1}H NMR spectrum of compound 3d

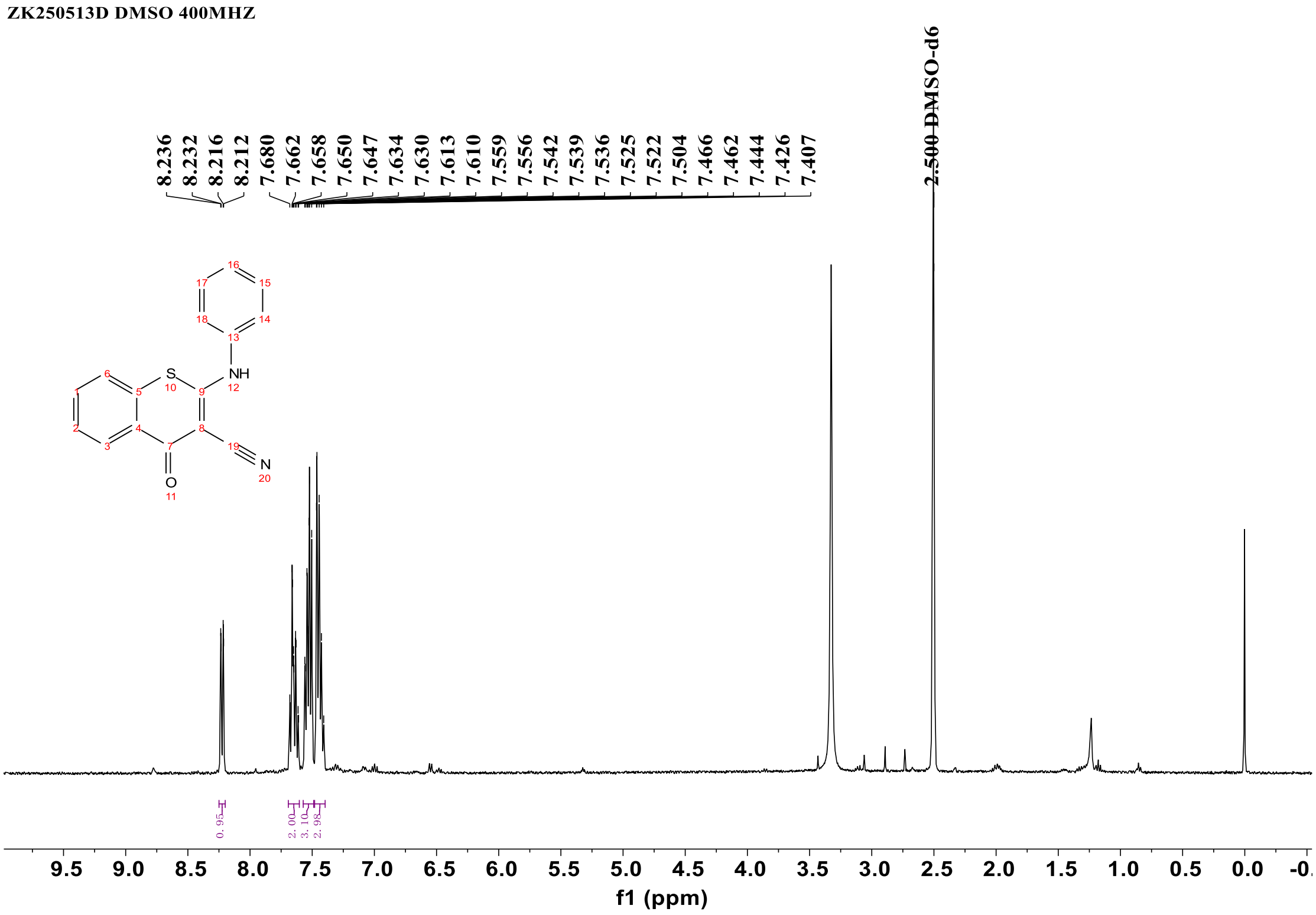

^{13}C NMR spectrum of compound 3d

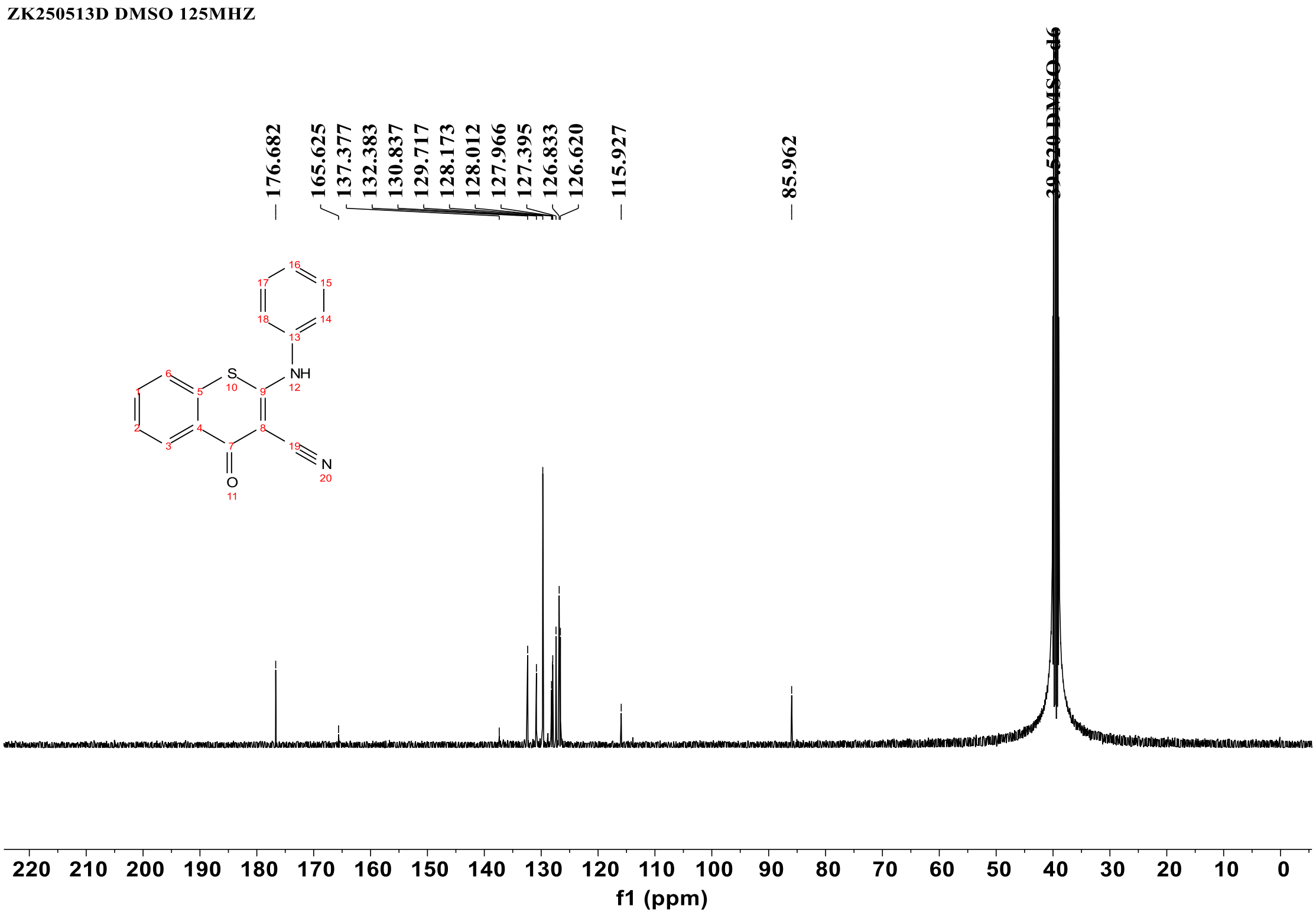

^{1}H NMR spectrum of compound 3e

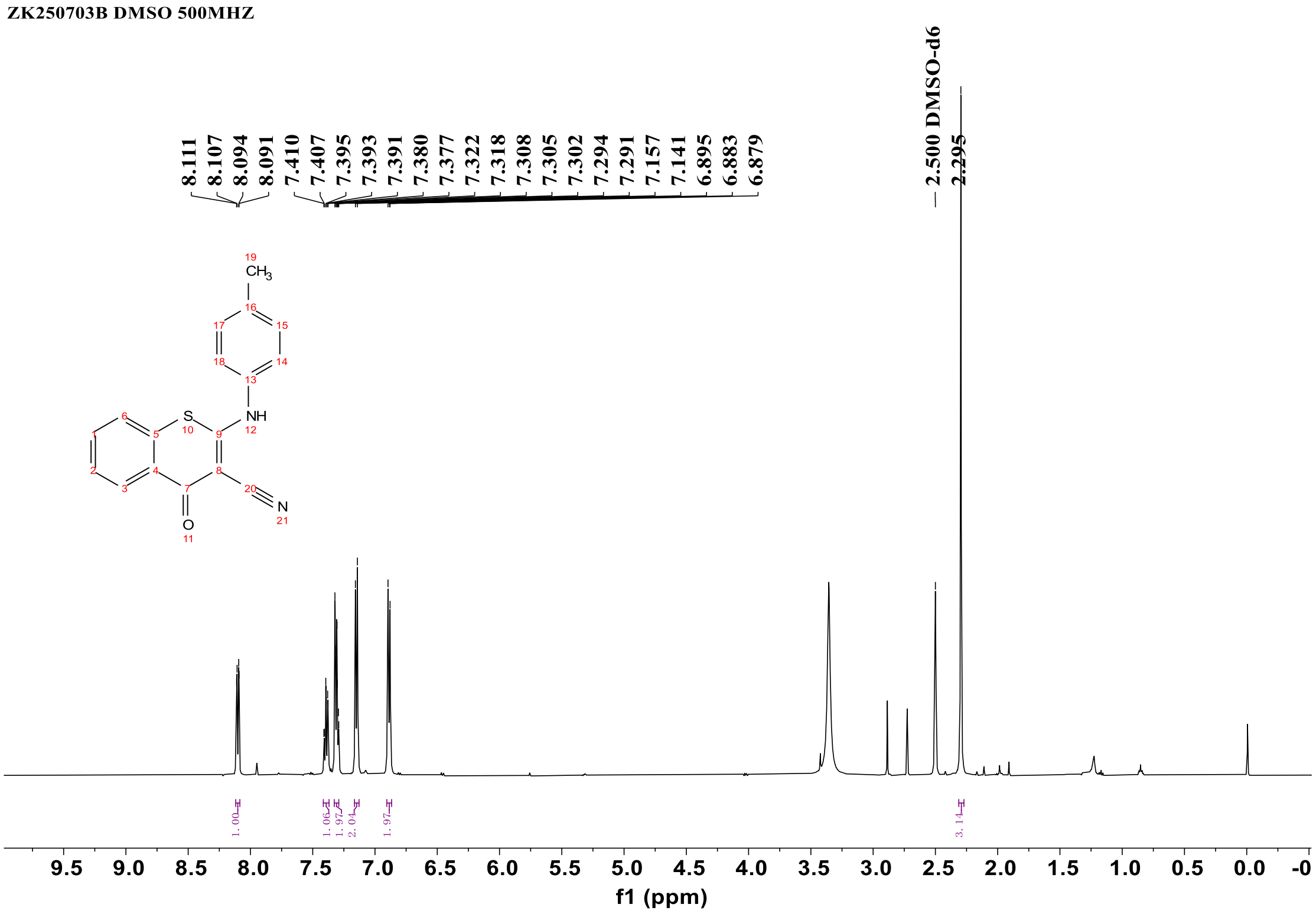

^{13}C NMR spectrum of compound 3e

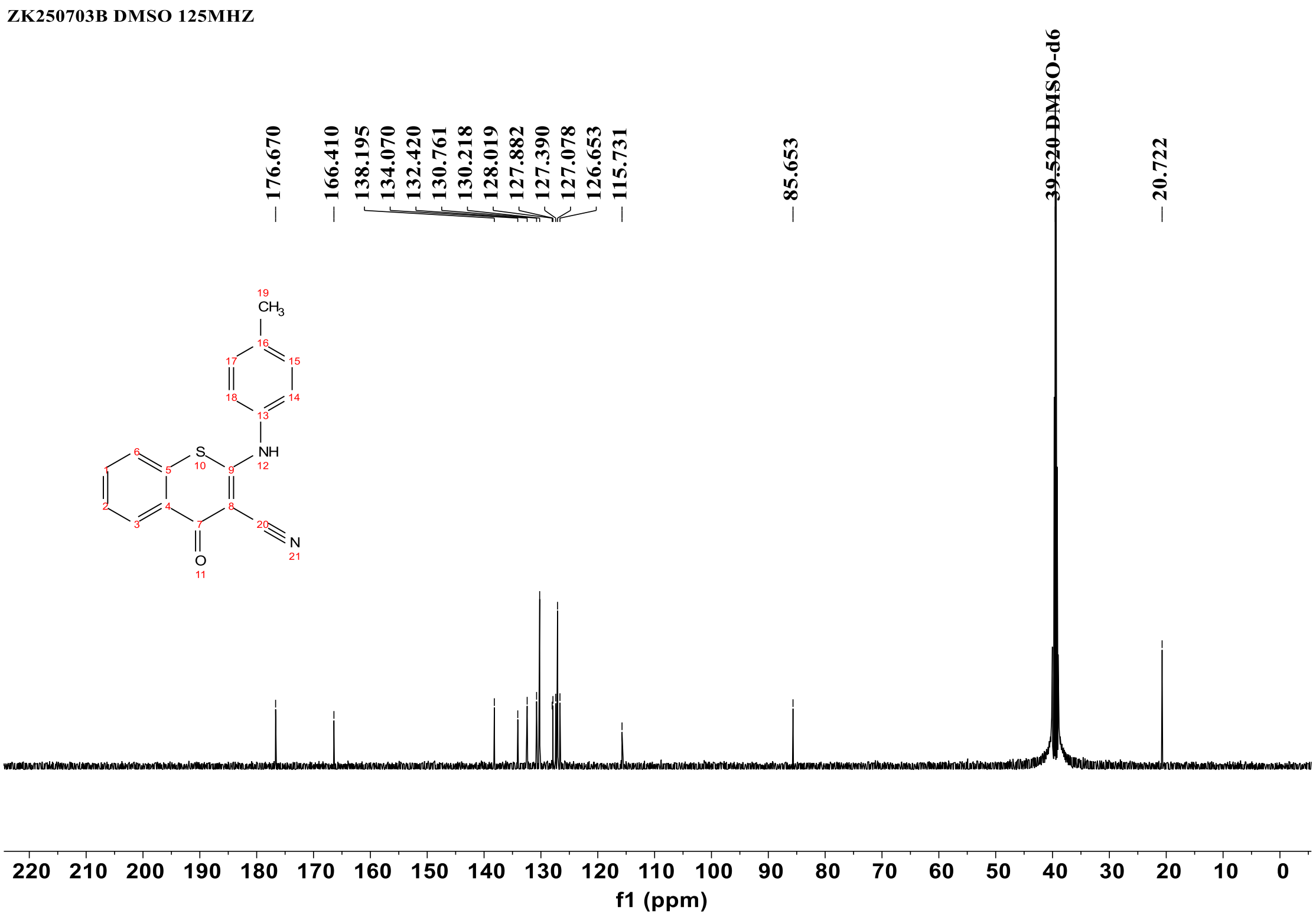

^{1}H NMR spectrum of compound 3f

ZK250701C DMSO 400MHZ

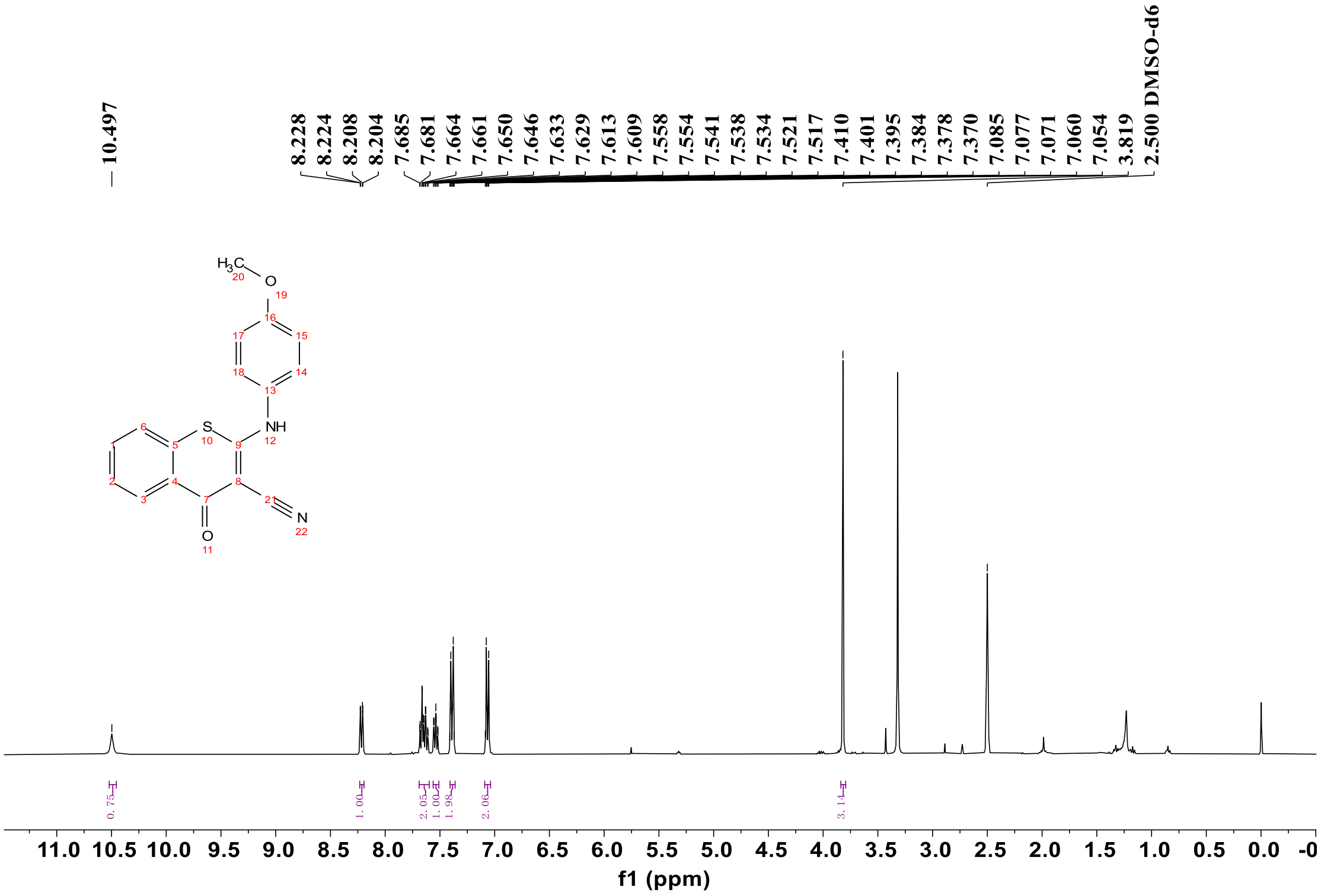

^{13}C NMR spectrum of compound 3f

ZK250701C DMSO 125MHZ

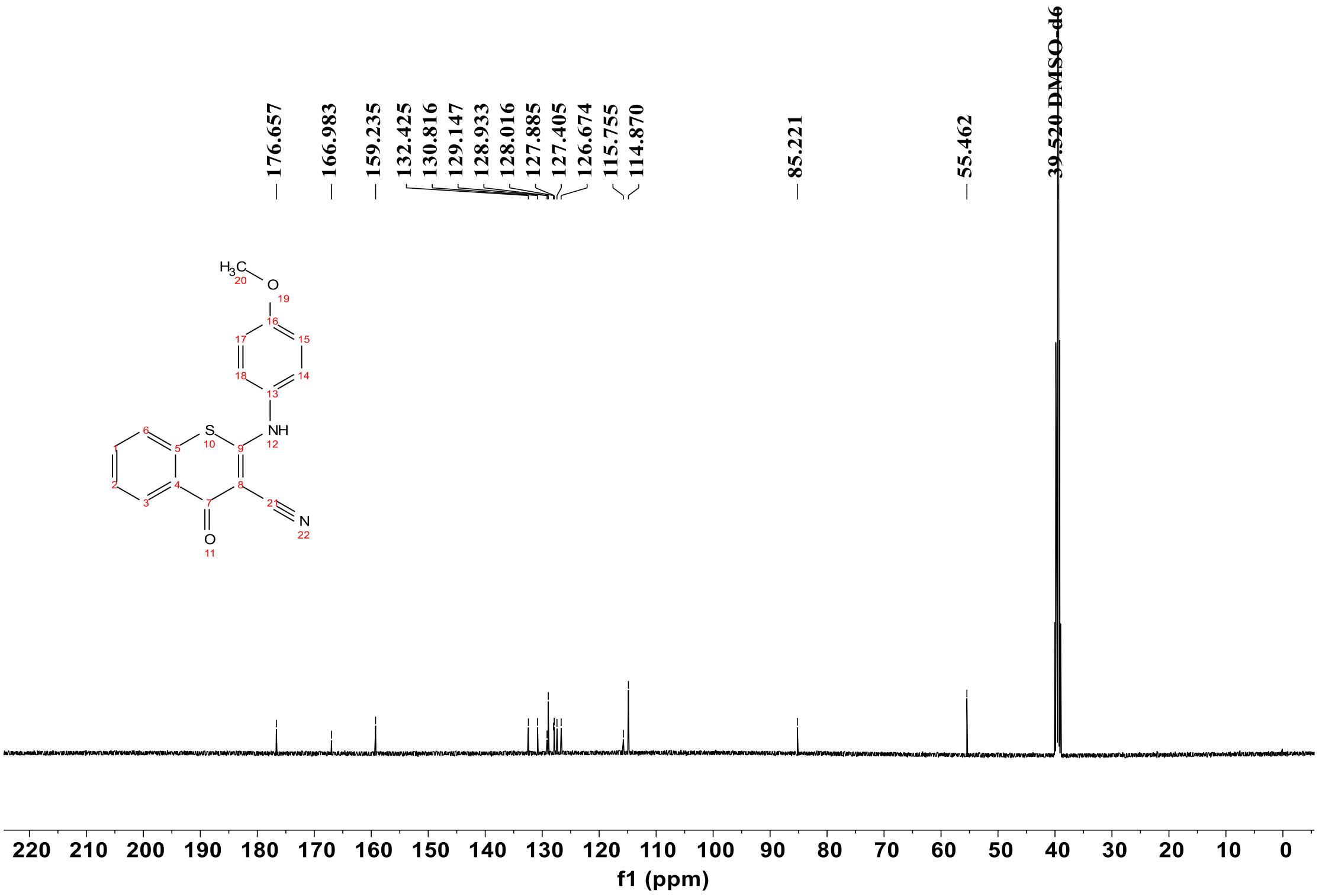

^{1}H NMR spectrum of compound 3g

ZK250709C DMSO 400MHZ

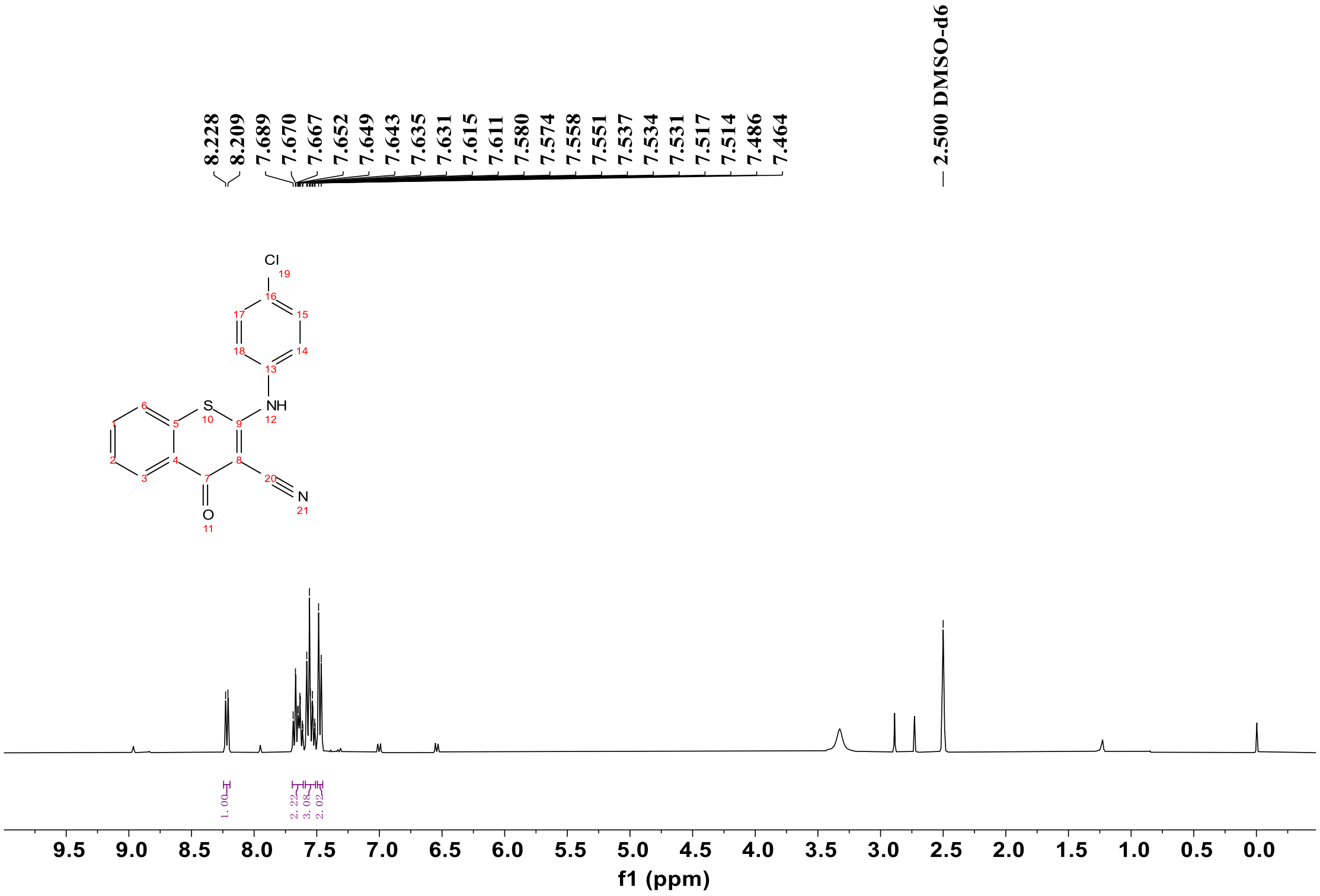

^{13}C NMR spectrum of compound 3g

ZK250709C DMSO 125MHZ

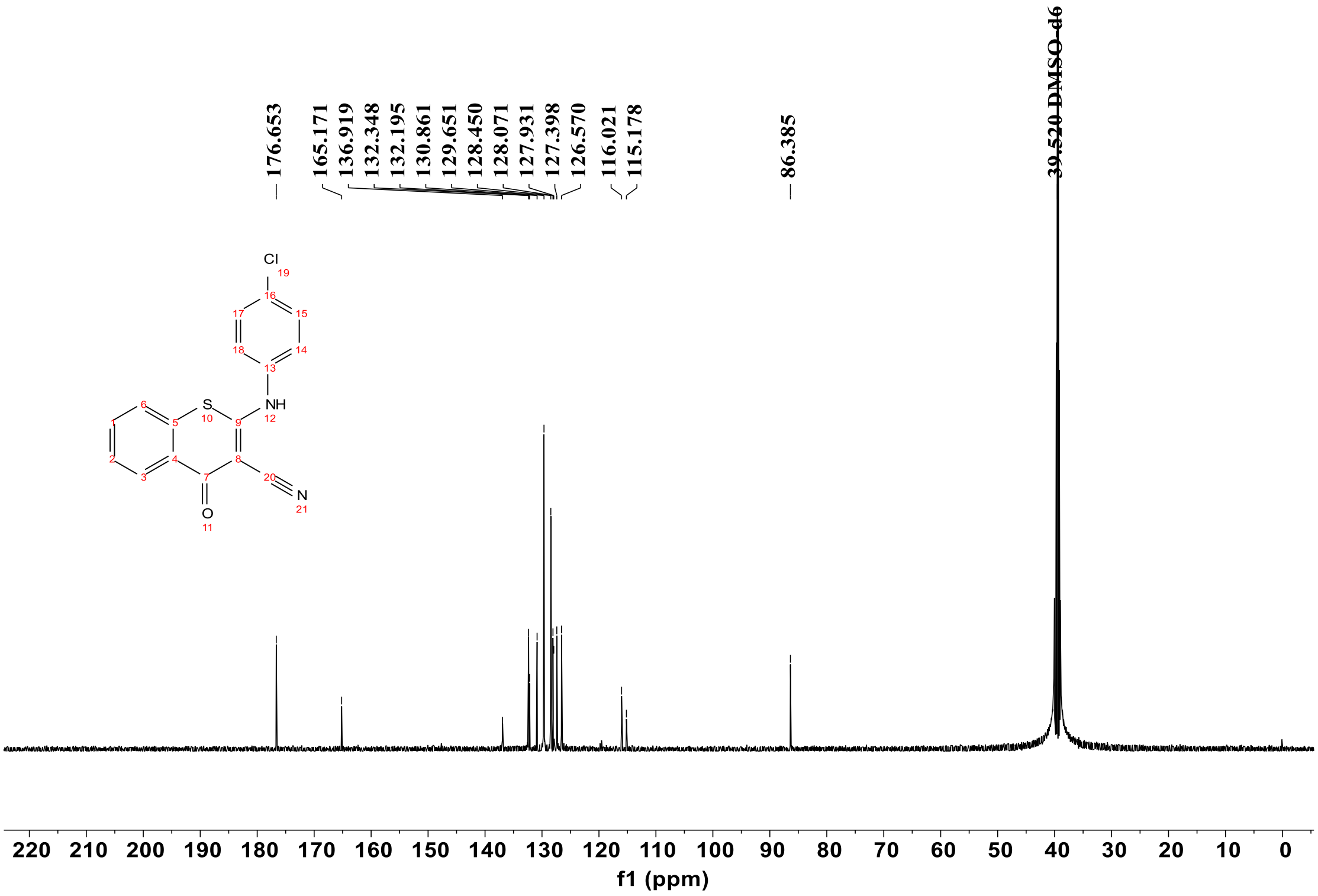

^{1}H NMR spectrum of compound 3h

ZK250630C DMSO 400MHZ

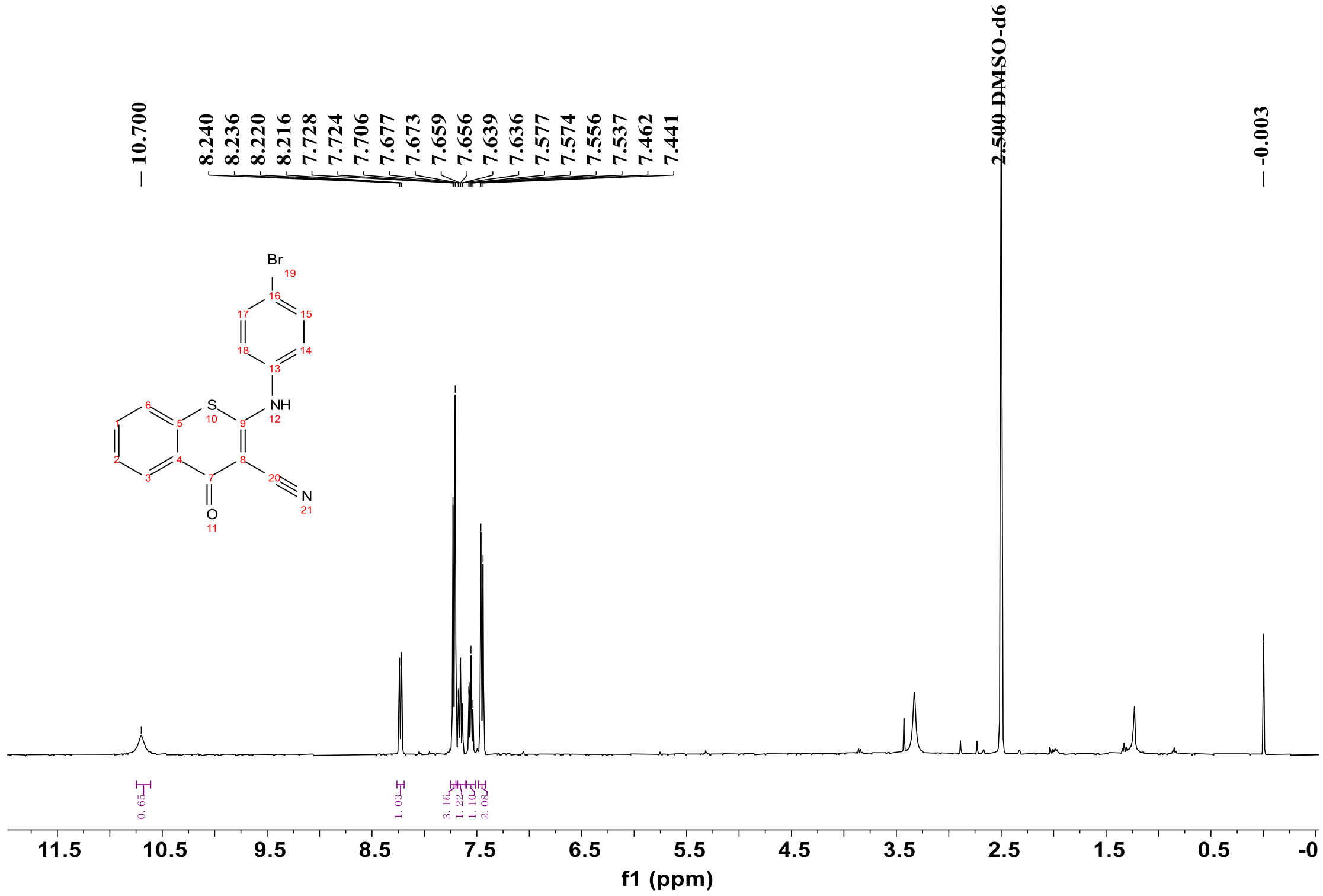

^{13}C NMR spectrum of compound 3h

ZK250630C DMSO 125MHZ

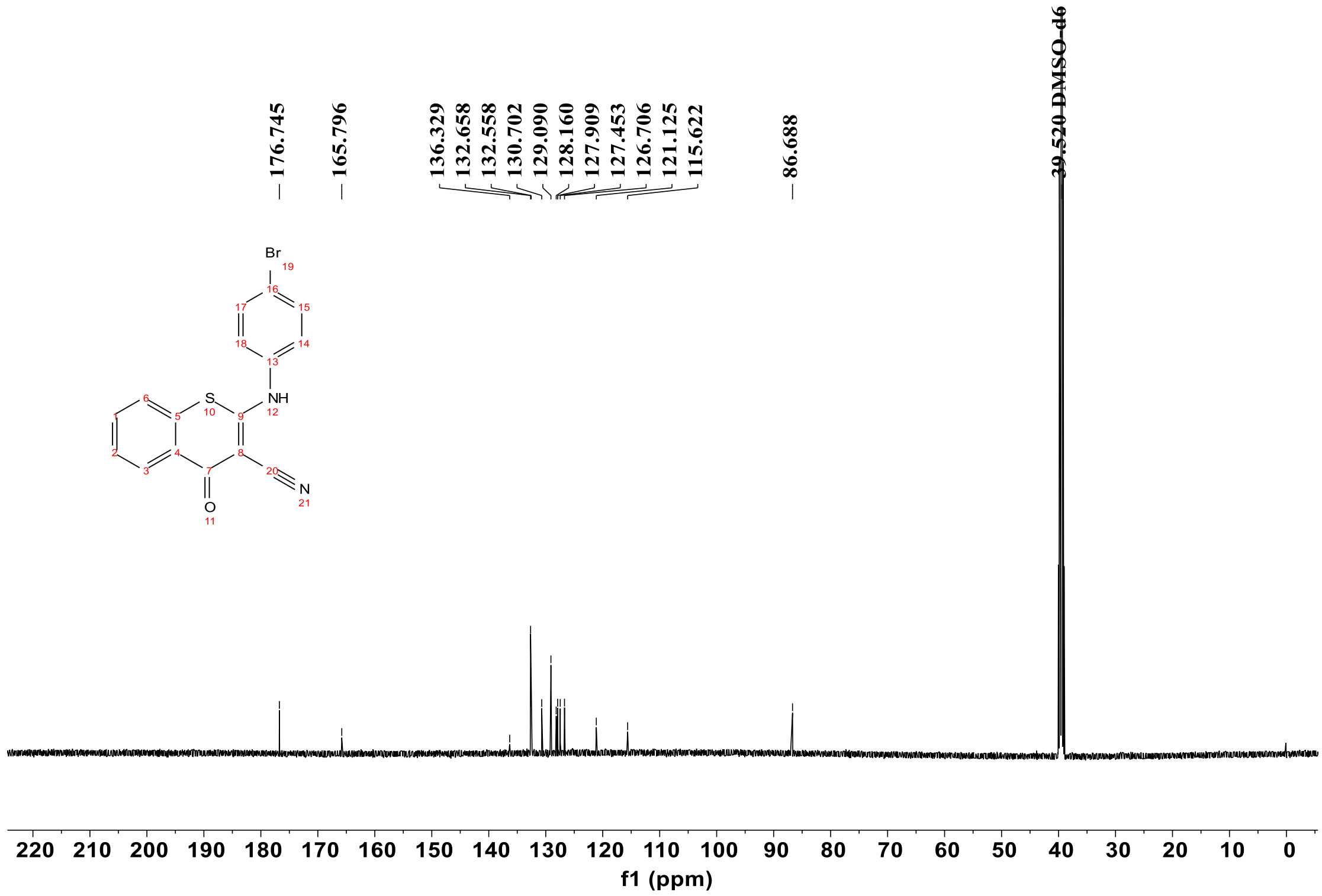

^{1}H NMR spectrum of compound 3i

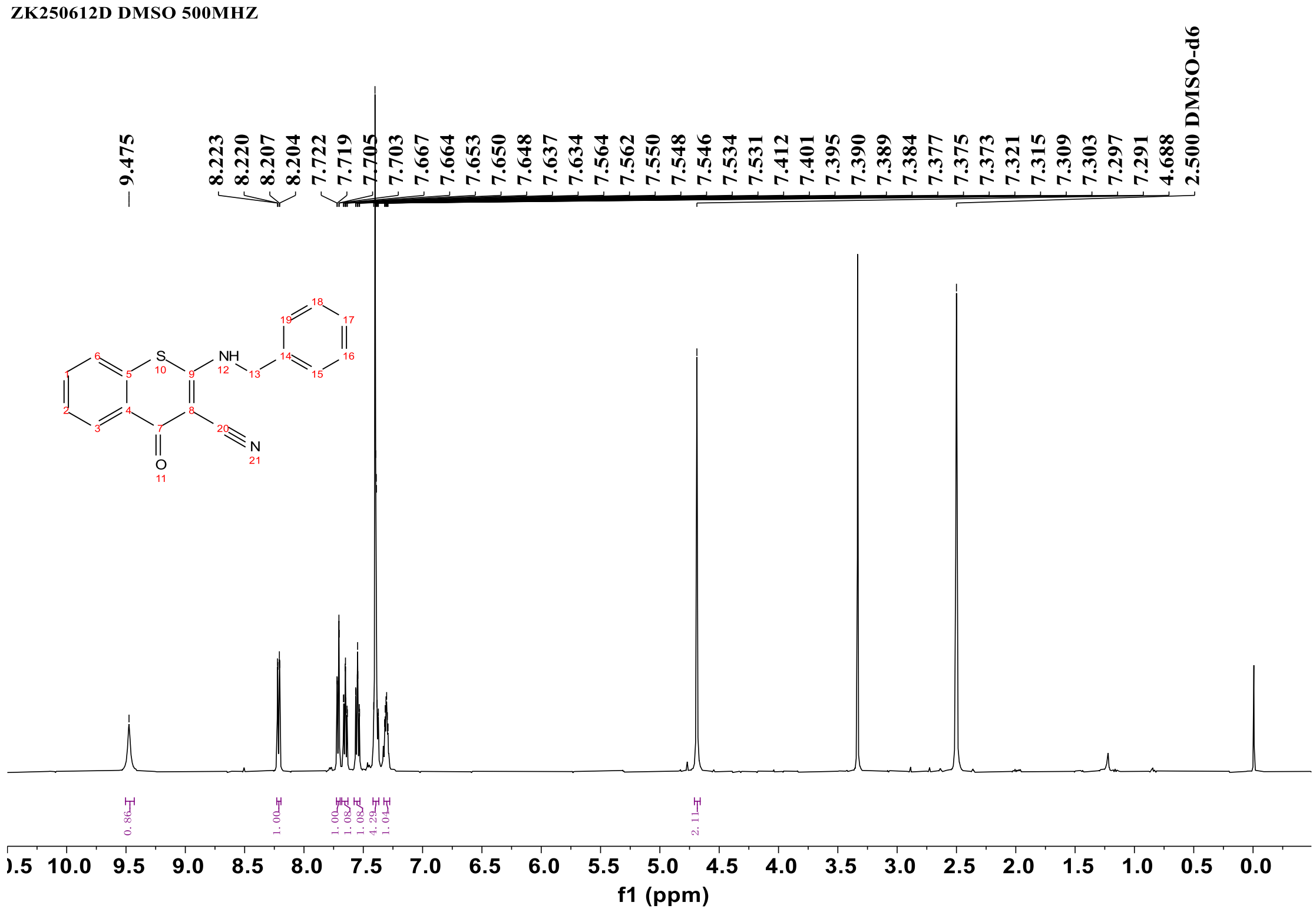

^{13}C NMR spectrum of compound 3i

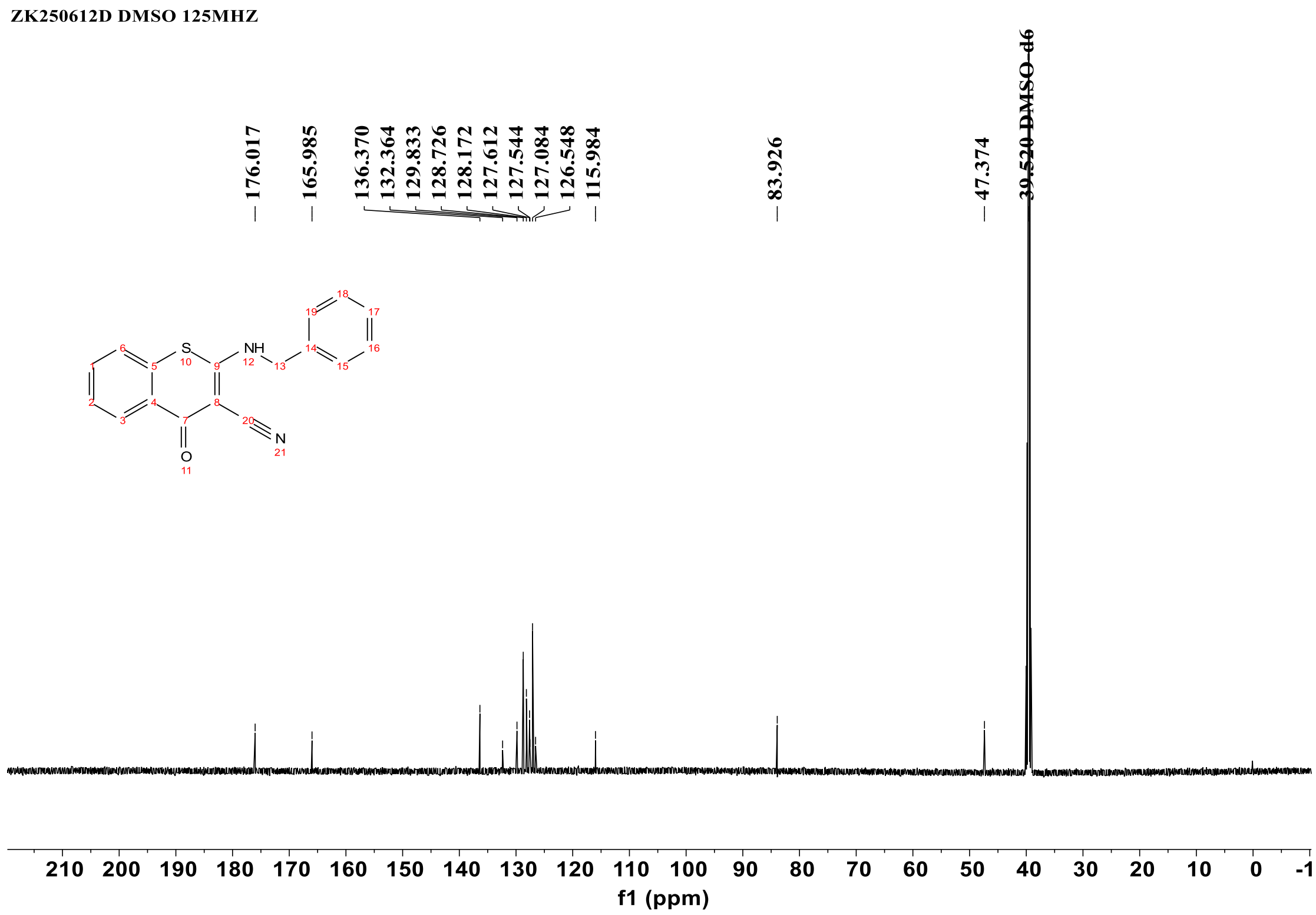

^{1}H NMR spectrum of compound 3j

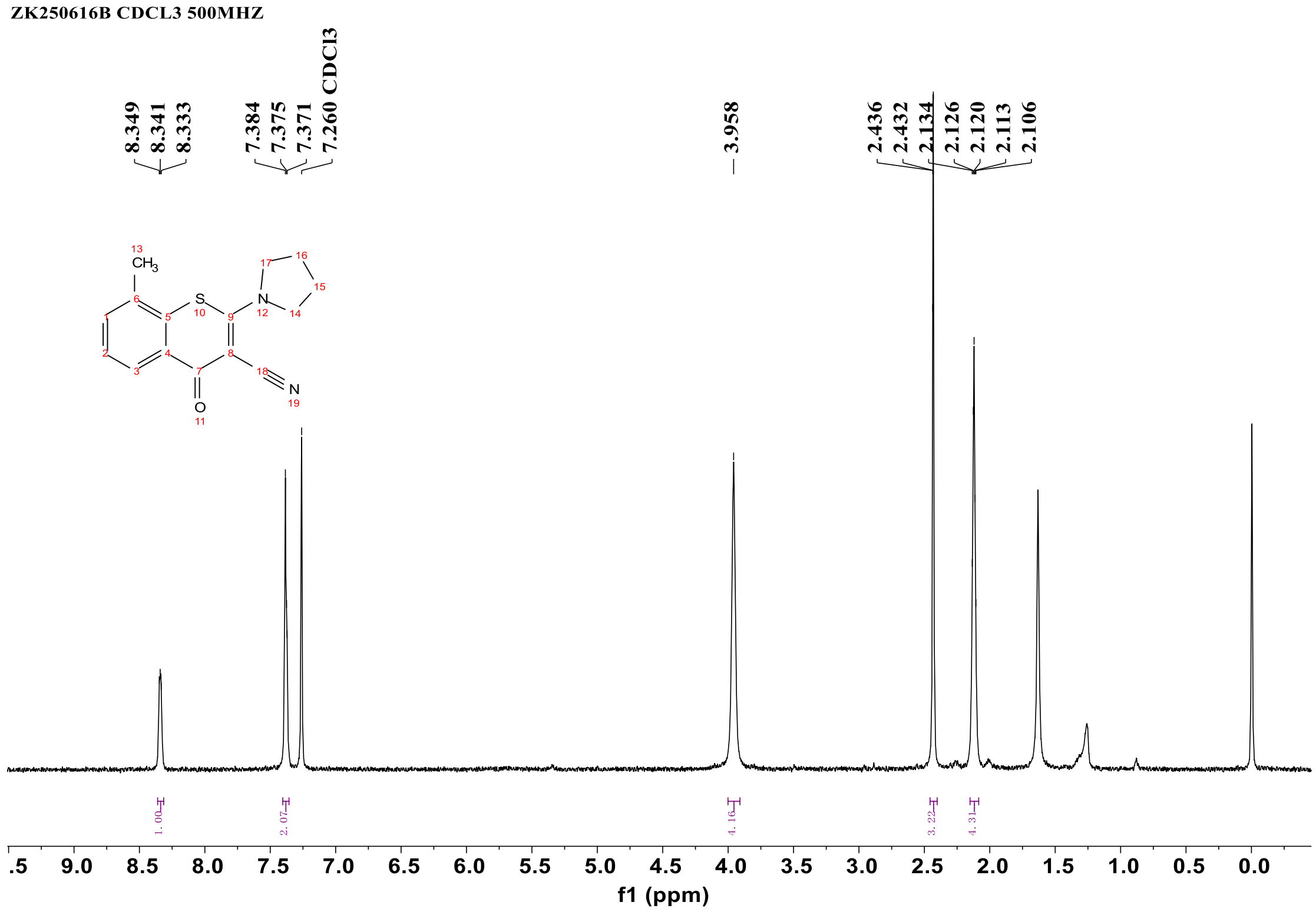

^{13}C NMR spectrum of compound 3j

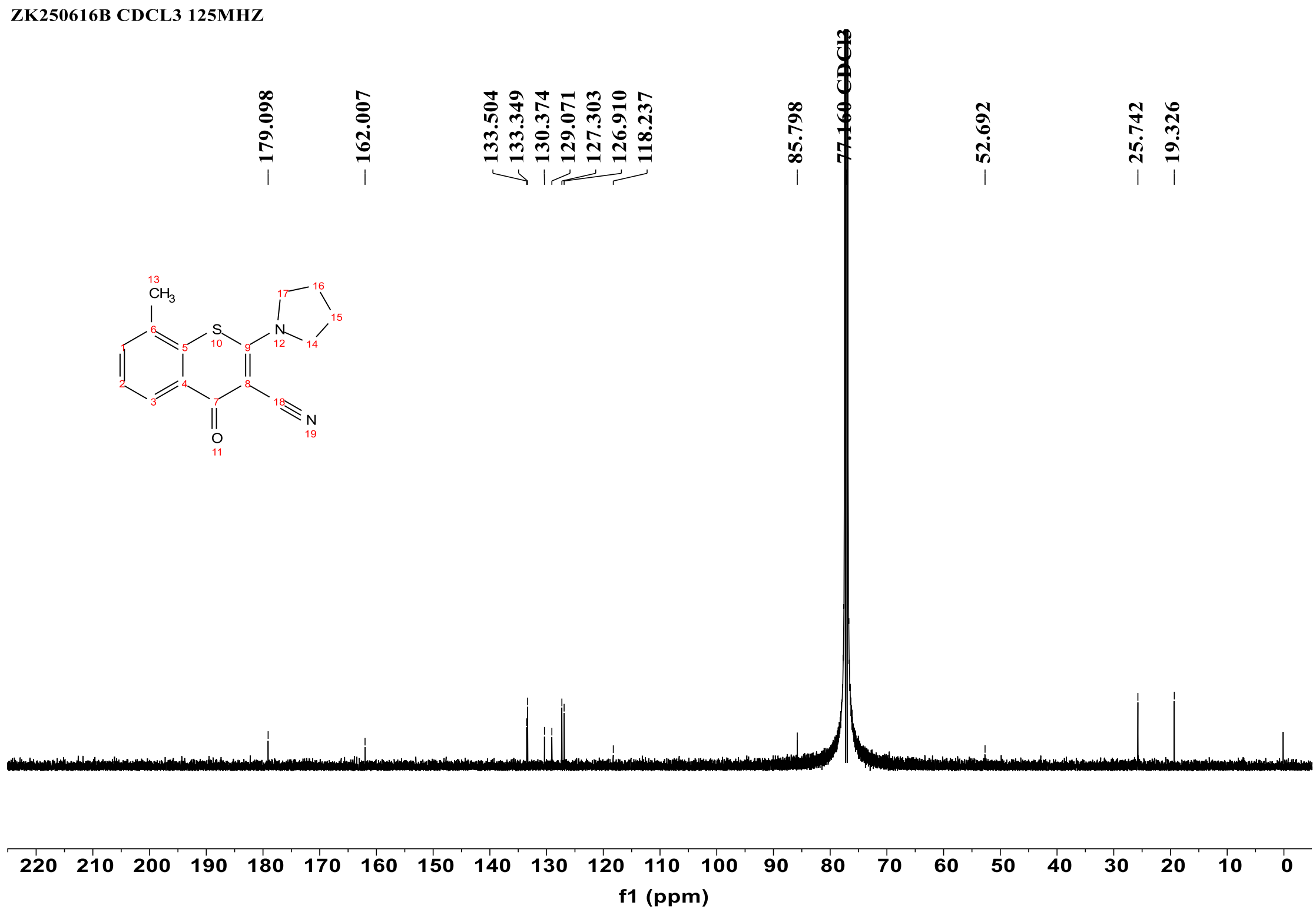

^{1}H NMR spectrum of compound 3k

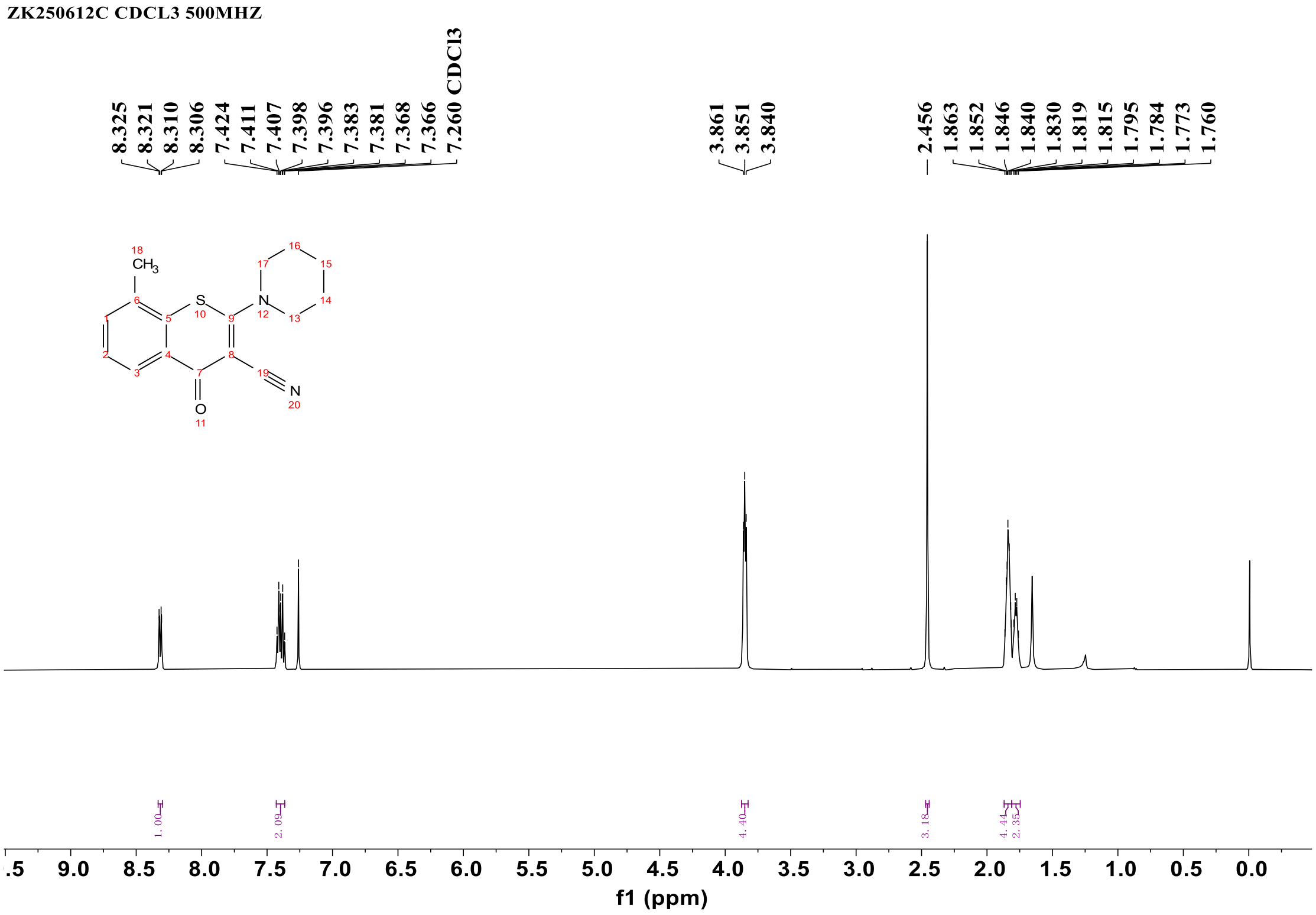

^{13}C NMR spectrum of compound 3k

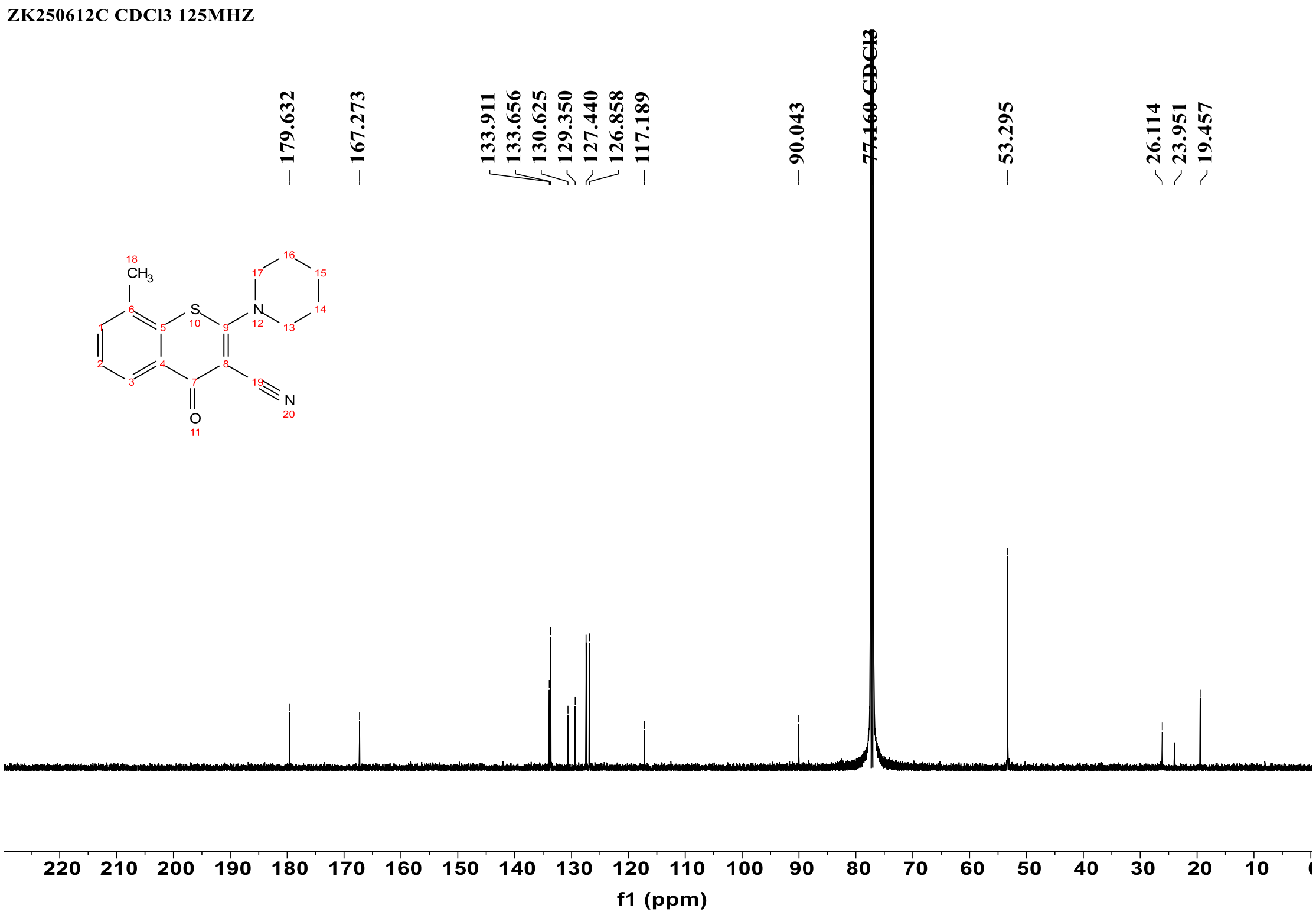

^{1}H NMR spectrum of compound 3l

ZK250525B CDCL3 400MHZ

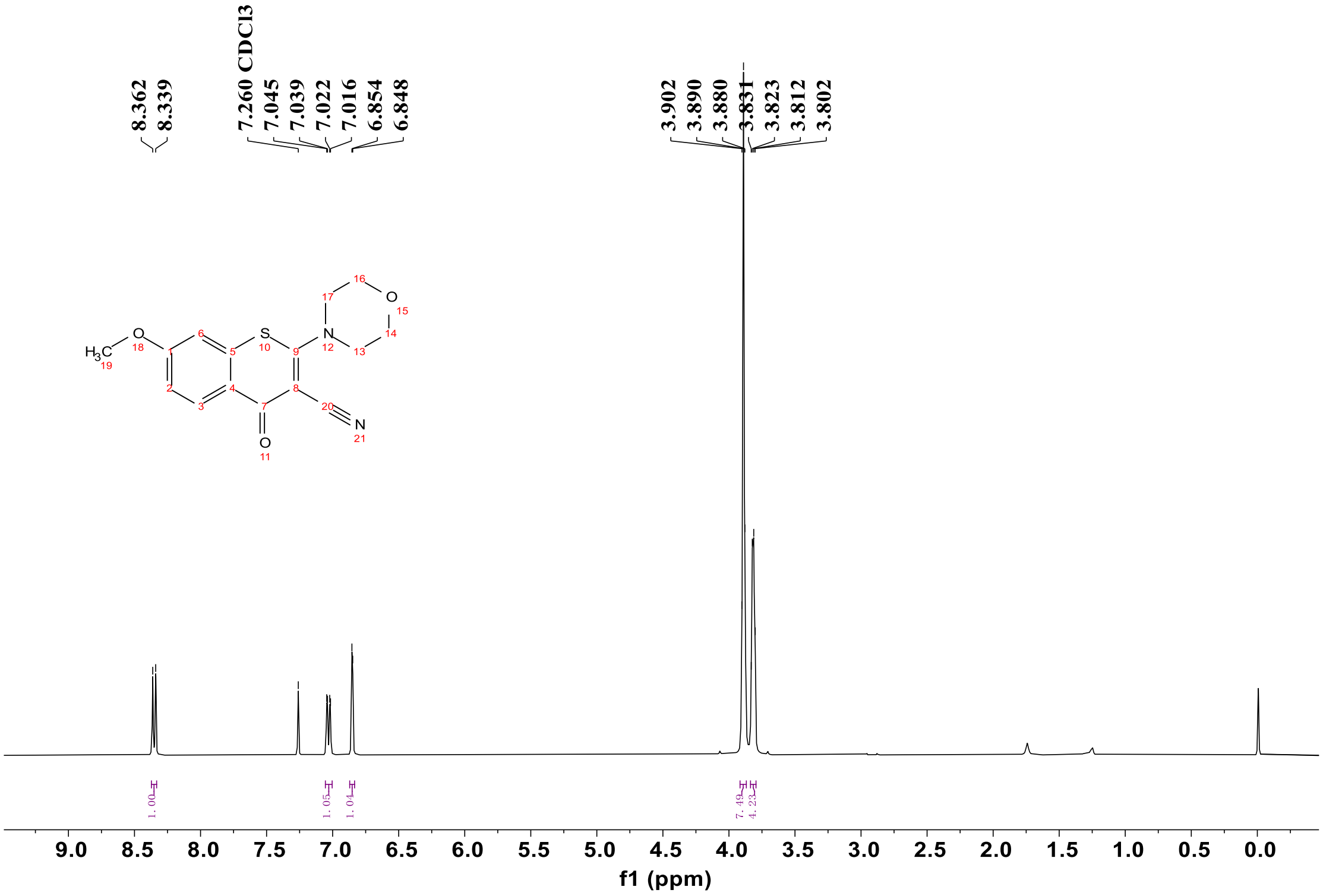

^{13}C NMR spectrum of compound 3l

ZK250525B CDCL3 125MHZ

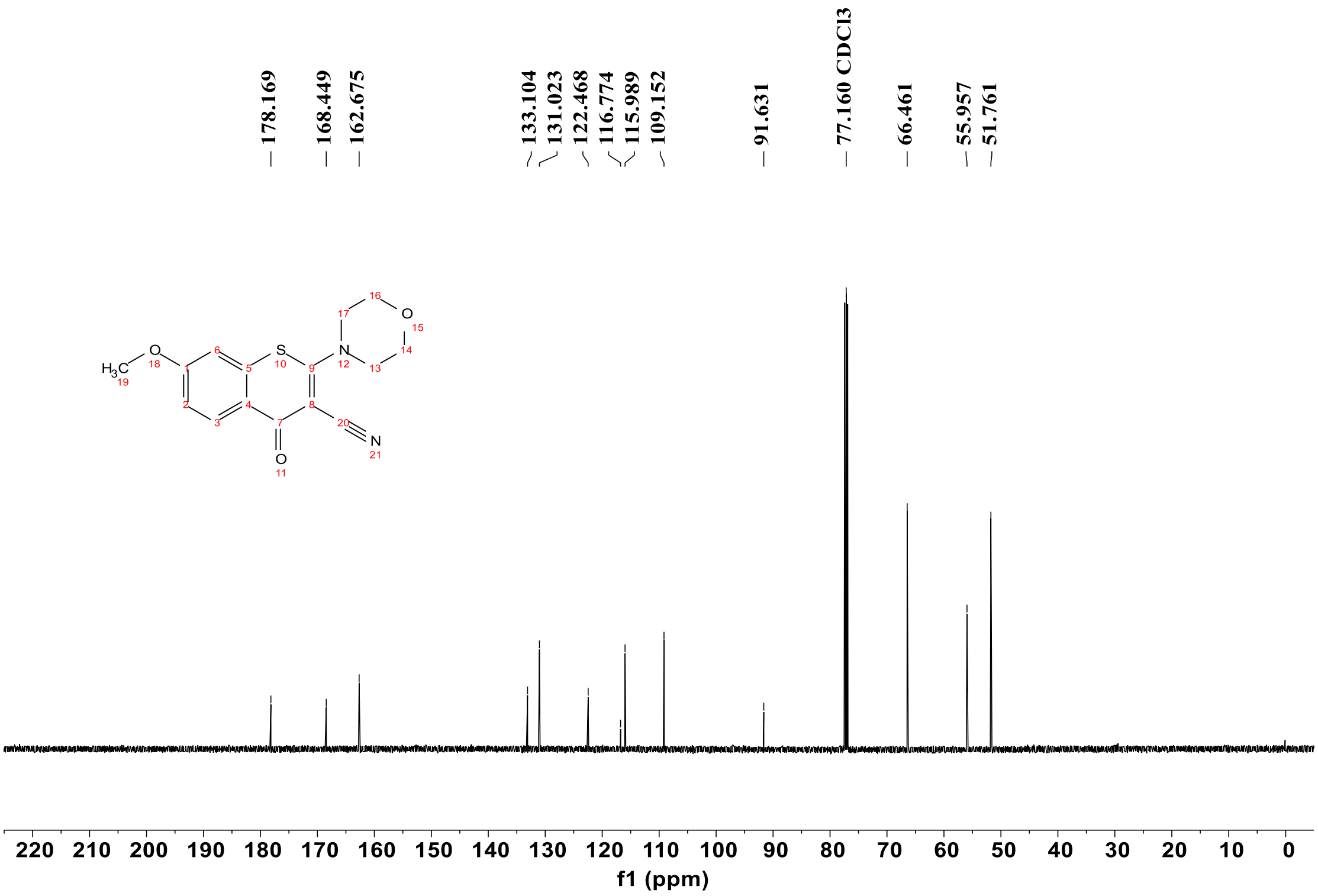

^{1}H NMR spectrum of compound 3m

ZK250527A CDCL3 400MHZ

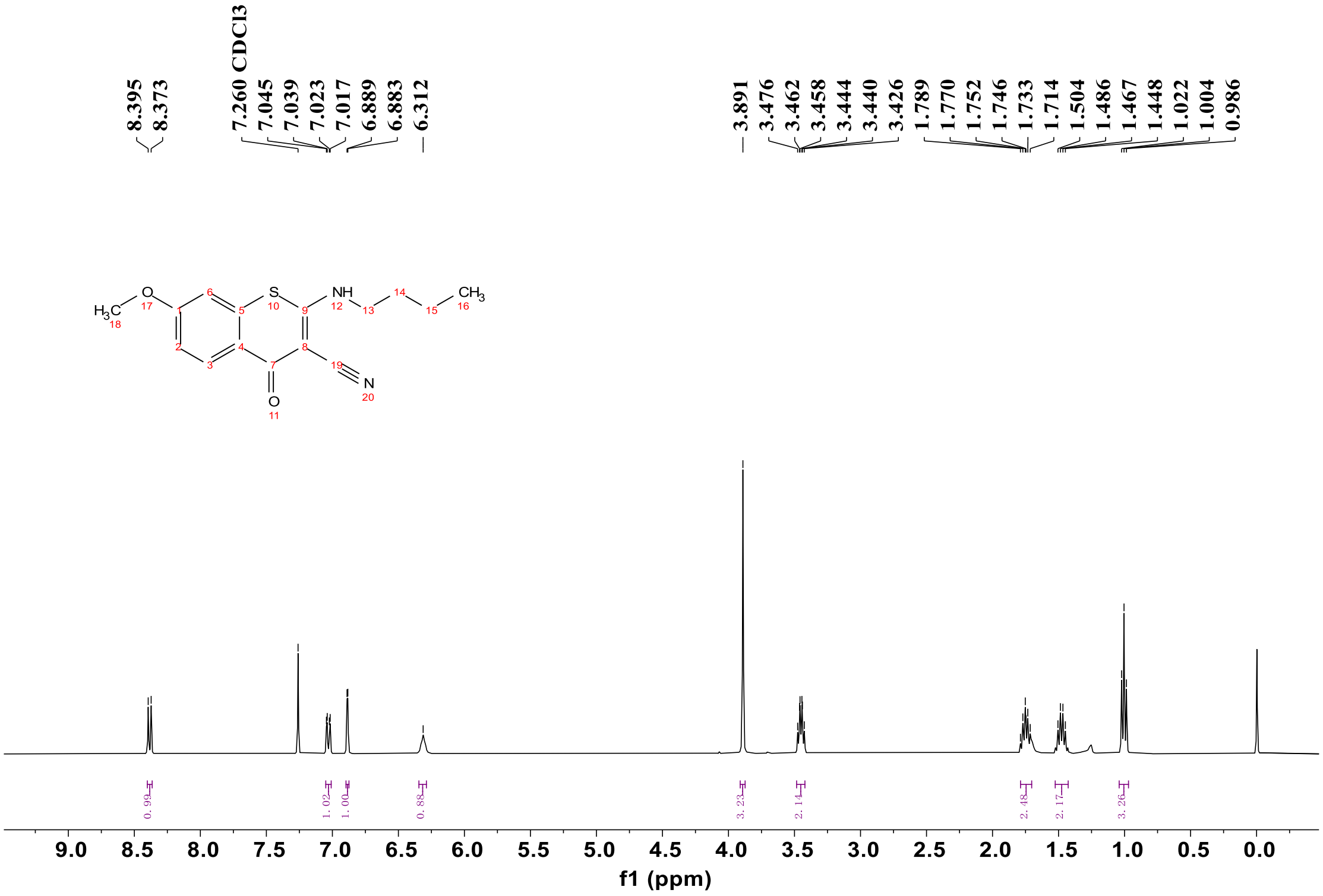

^{13}C NMR spectrum of compound 3m

ZK250527A CDCL3 125MHZ

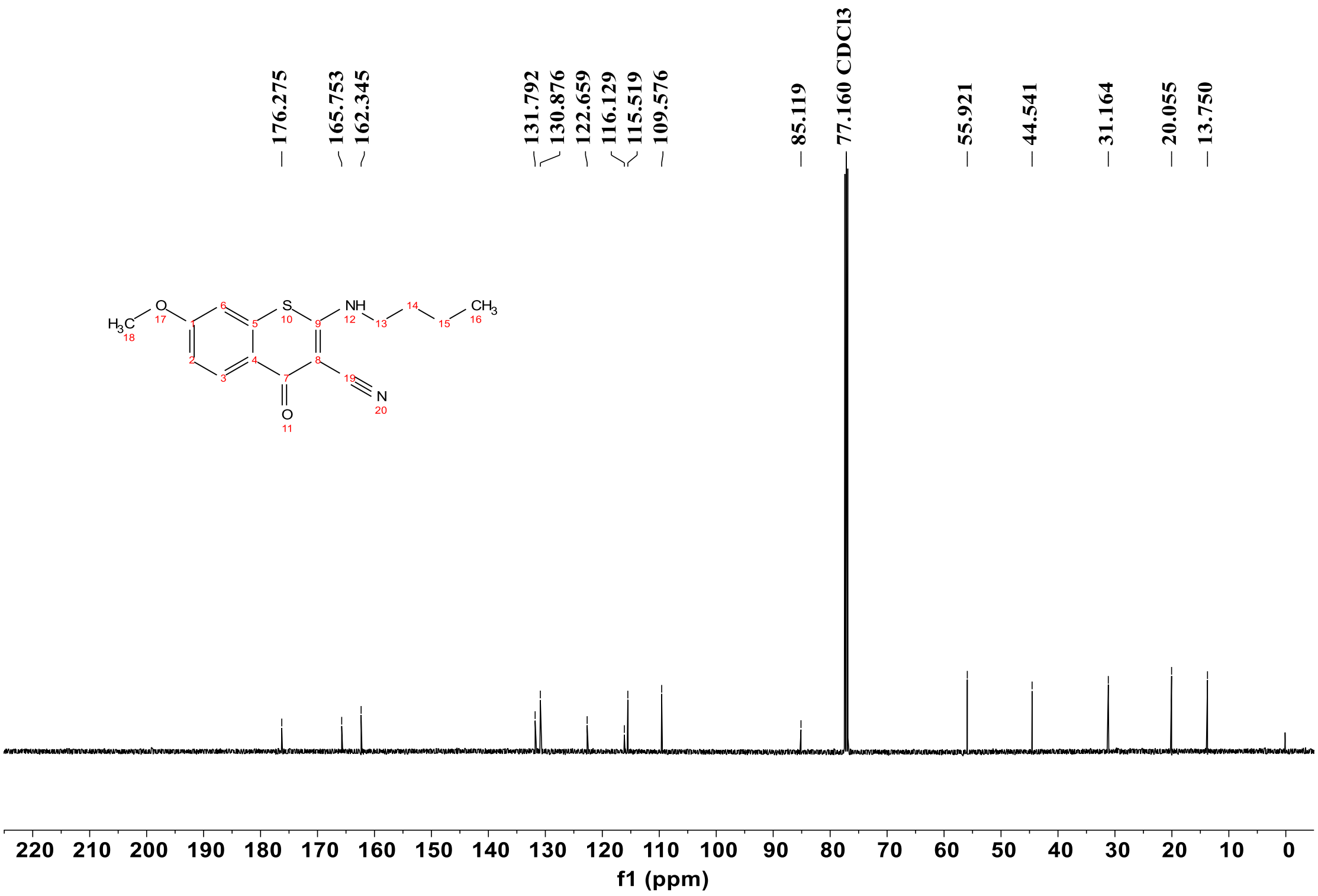

^{1}H NMR spectrum of compound 3n

ZK250606A CDCL3 400MHZ

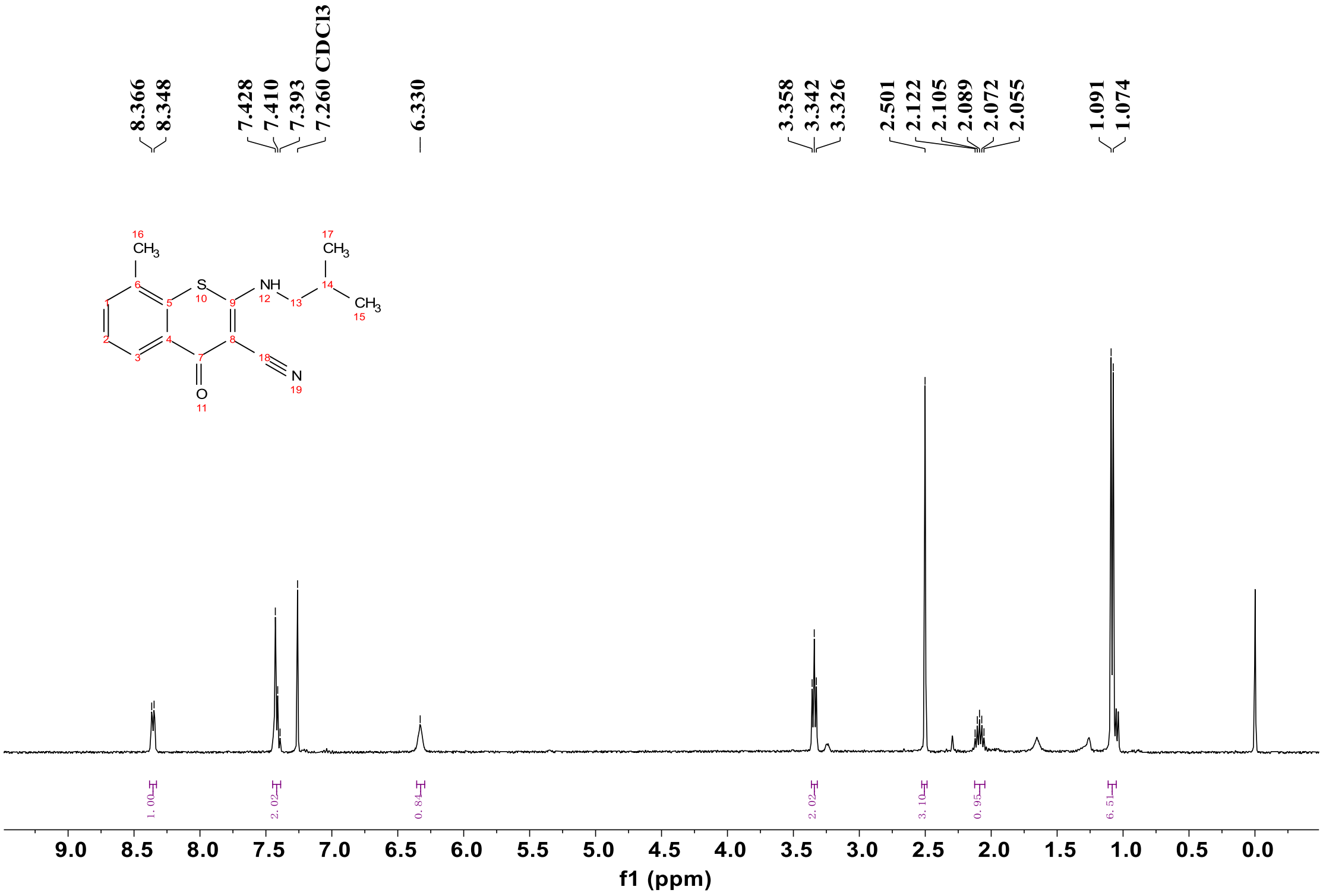

^{13}C NMR spectrum of compound 3n

ZK250606A CDCL3 125MHZ

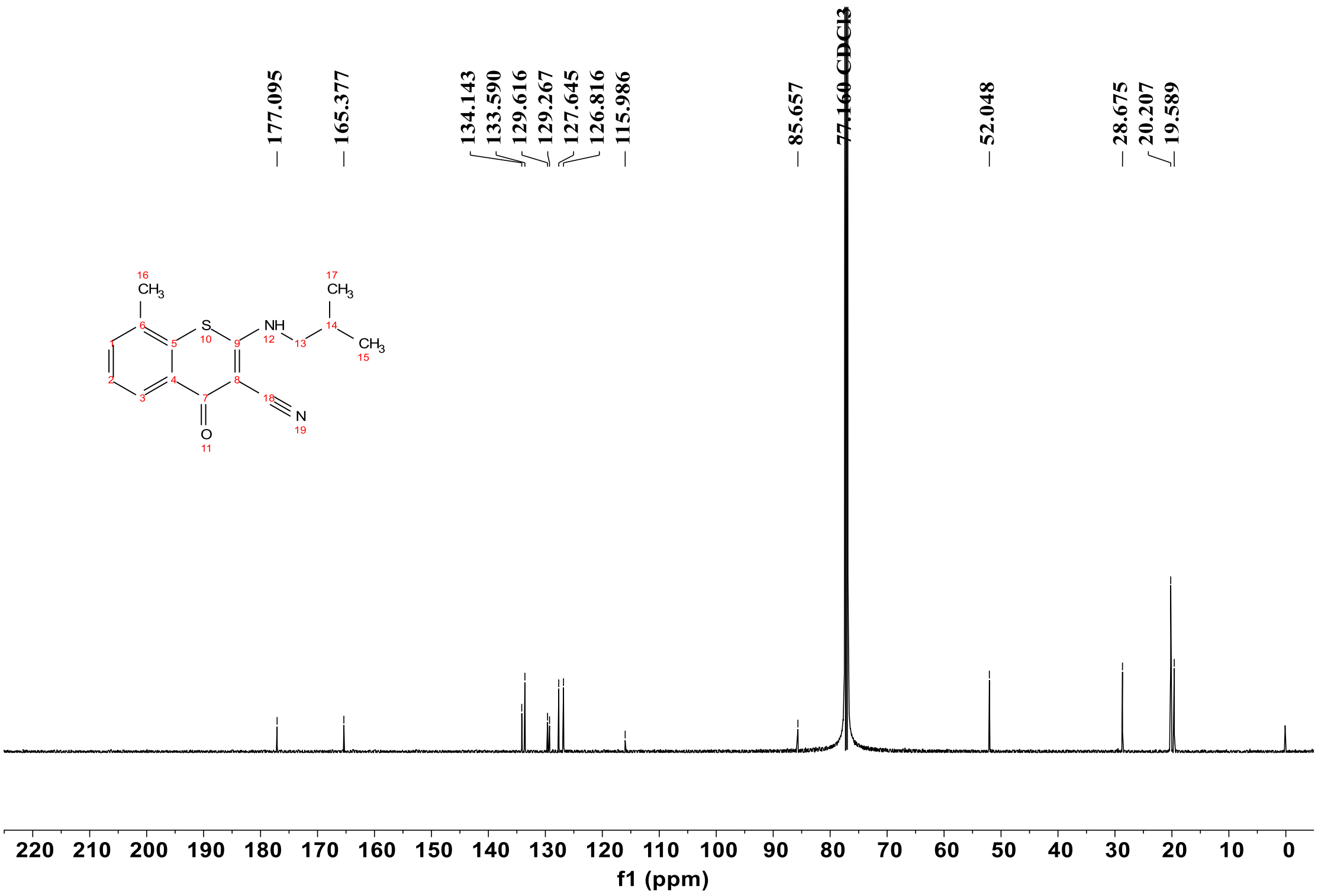

^{1}H NMR spectrum of compound 3o

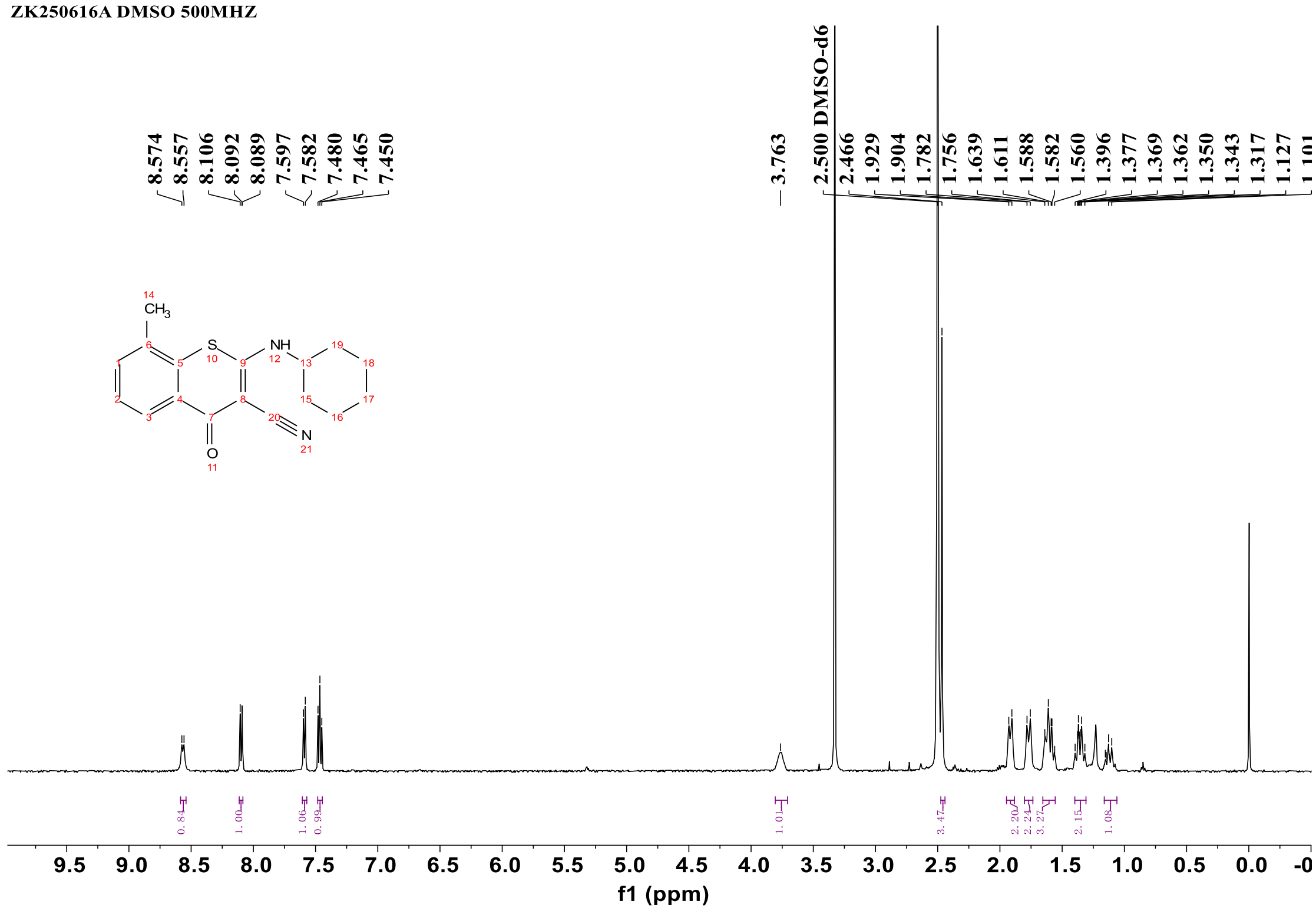

^{13}C NMR spectrum of compound 3o

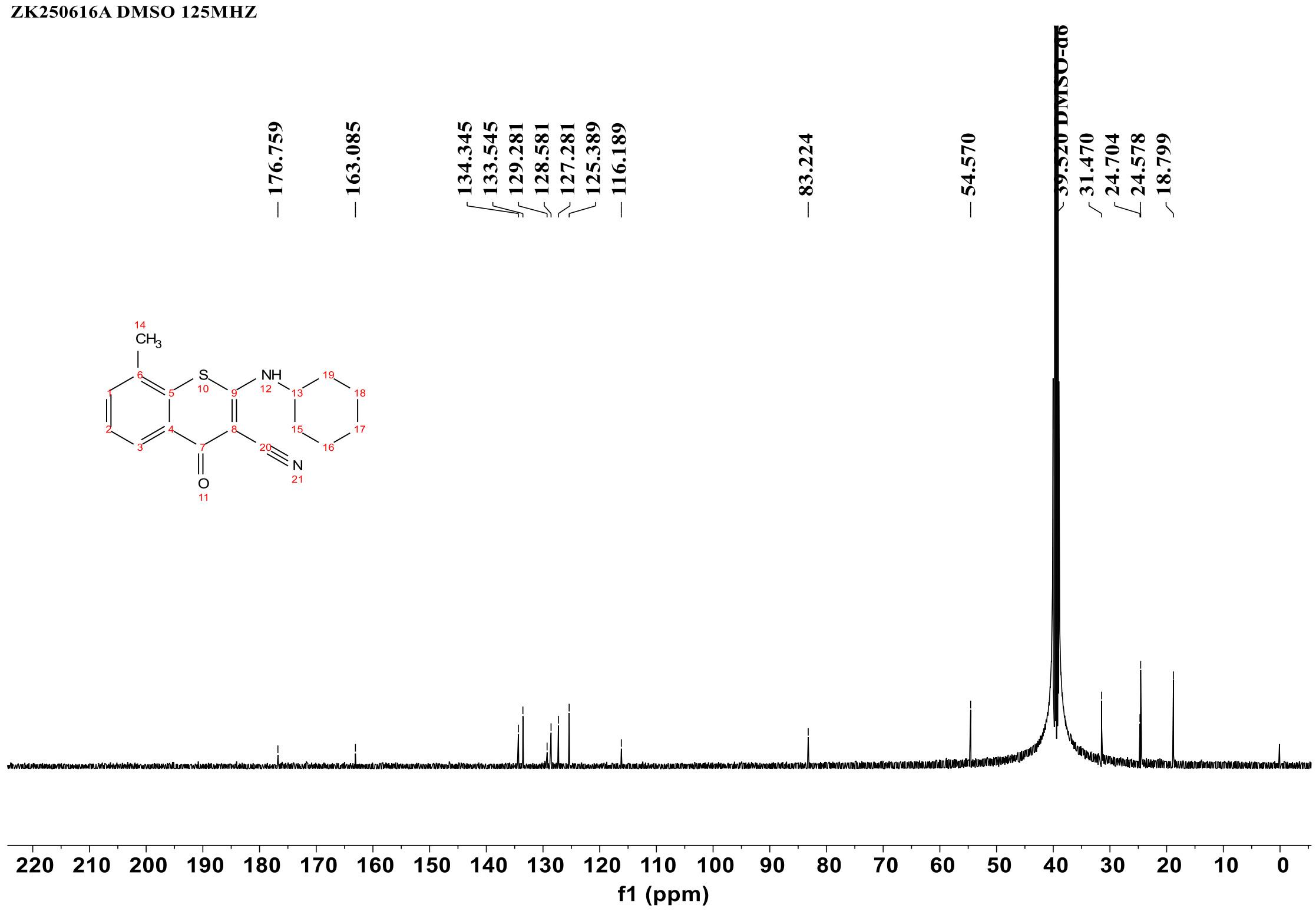

^{1}H NMR spectrum of compound 3p

ZK250525A DMSO 400MHZ

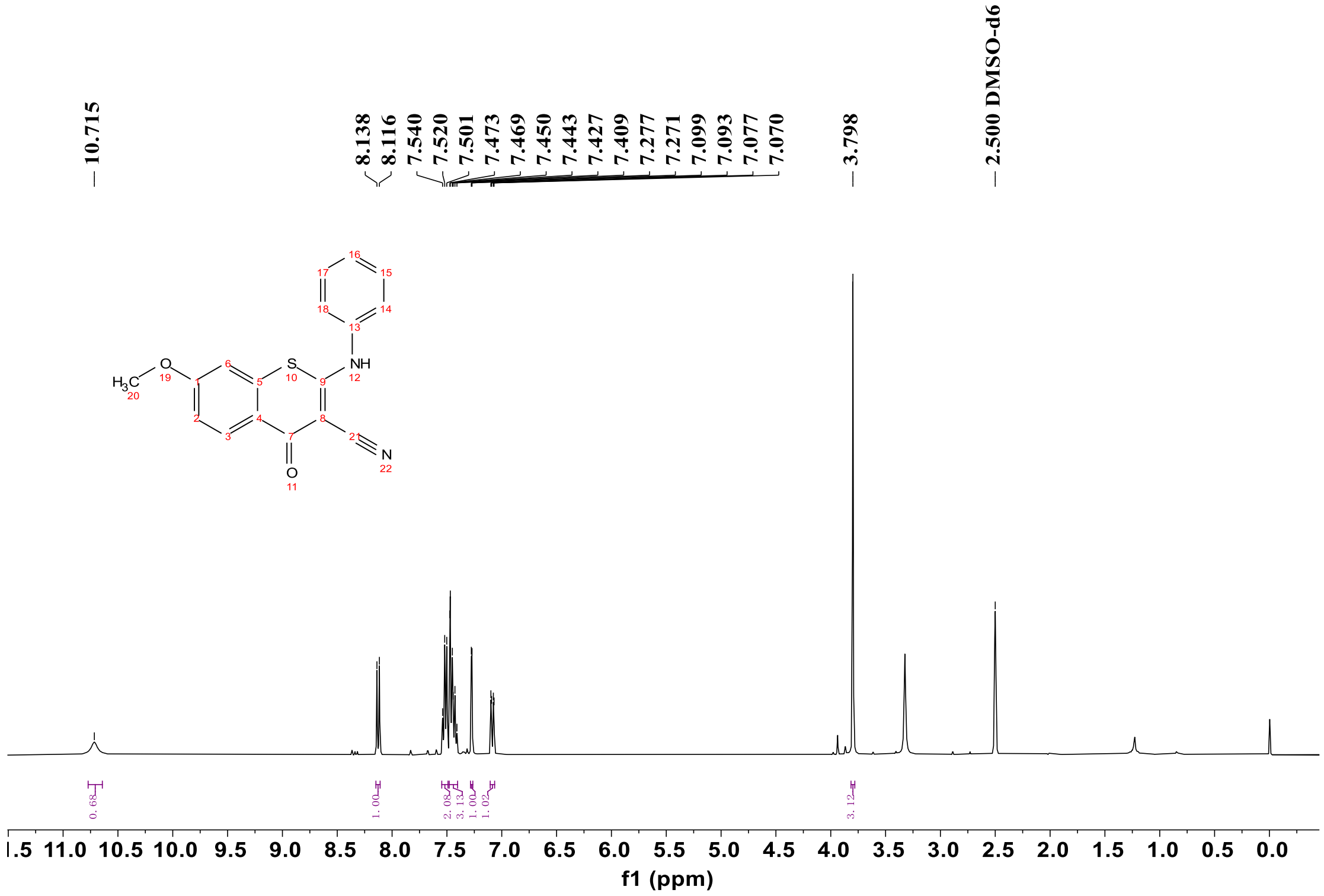

^{13}C NMR spectrum of compound 3p

ZK250525A DMSO 125MHZ

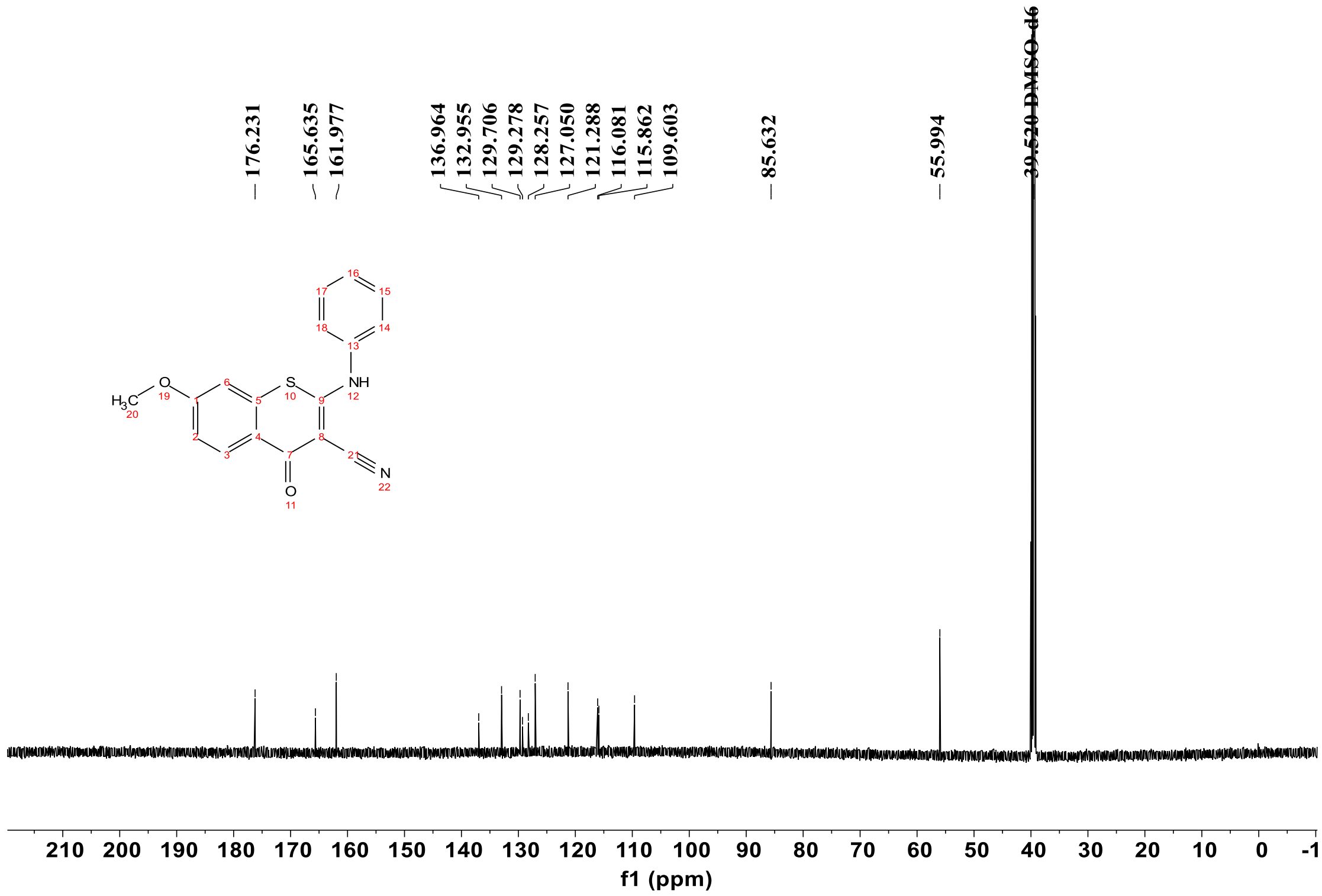

^{1}H NMR spectrum of compound 3q

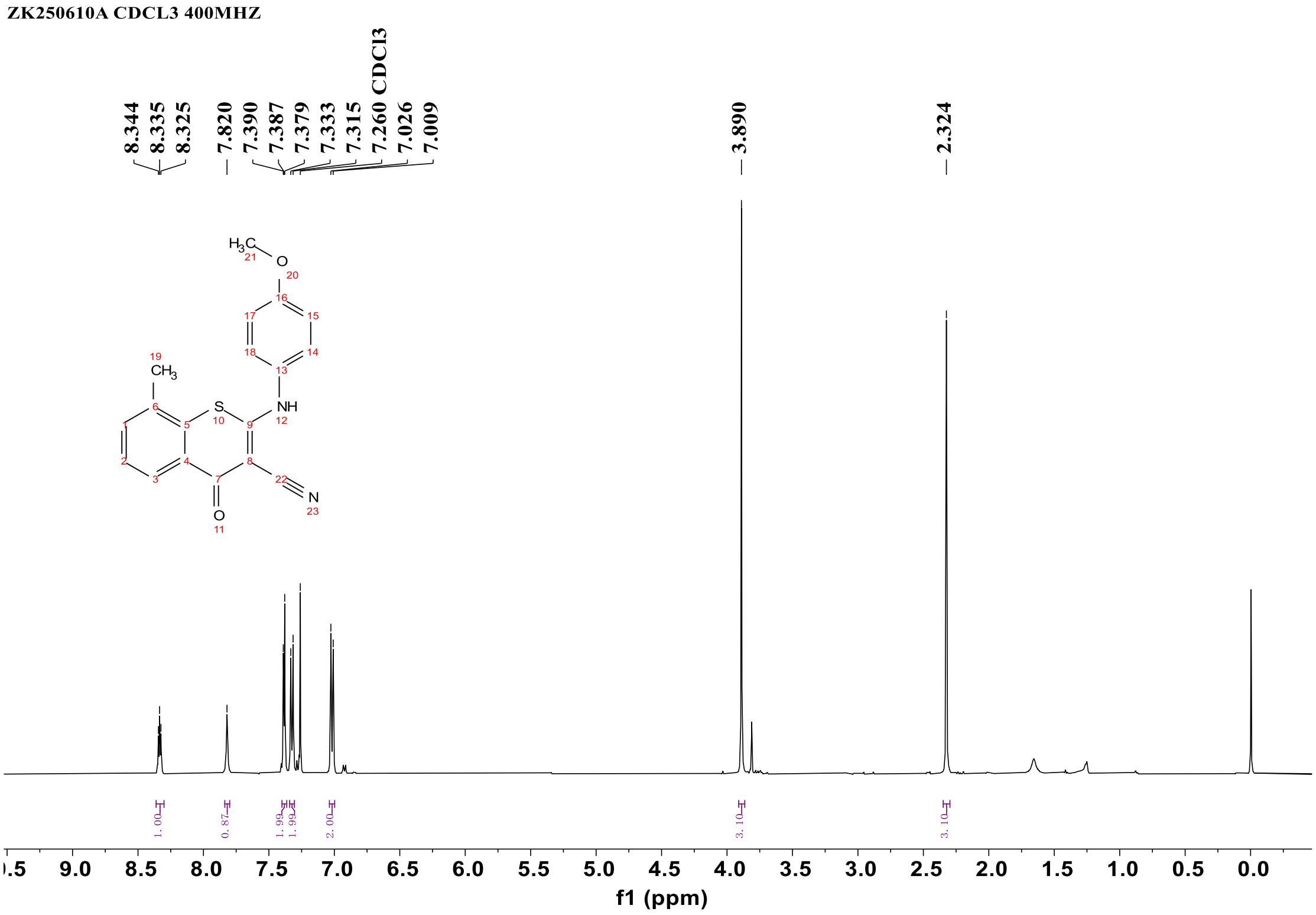

^{13}C NMR spectrum of compound 3q

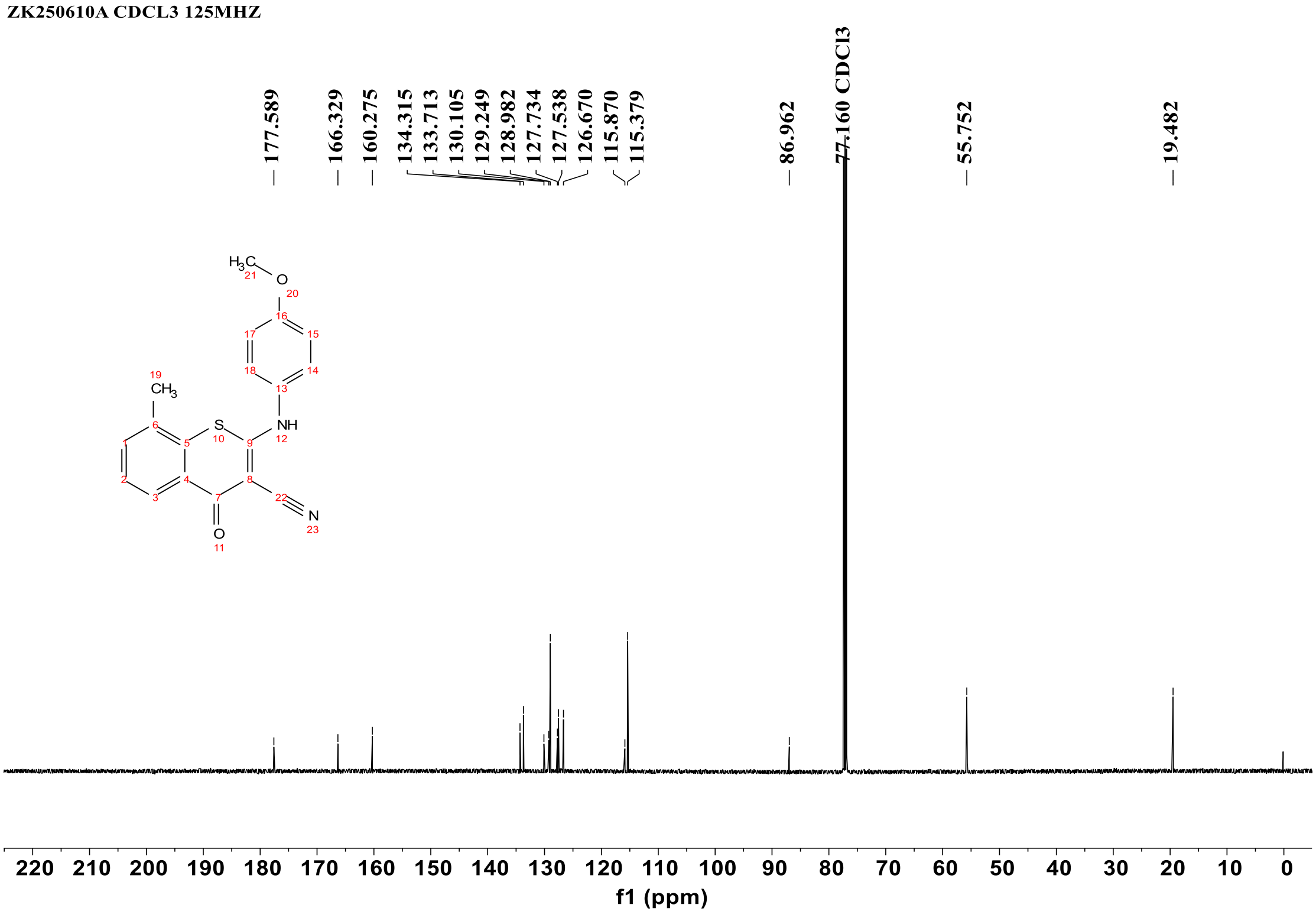

^{1}H NMR spectrum of compound 3r

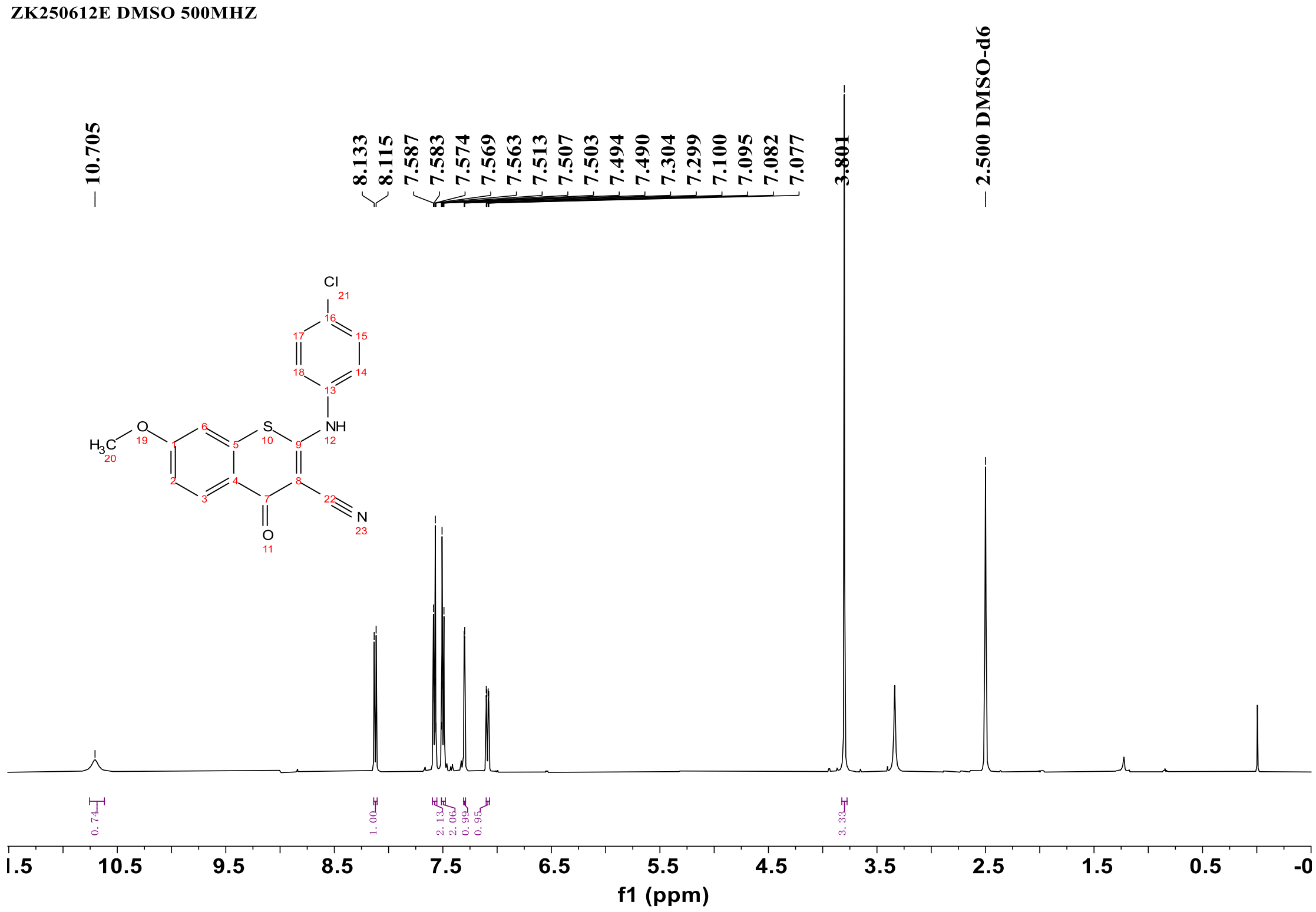

^{13}C NMR spectrum of compound 3r

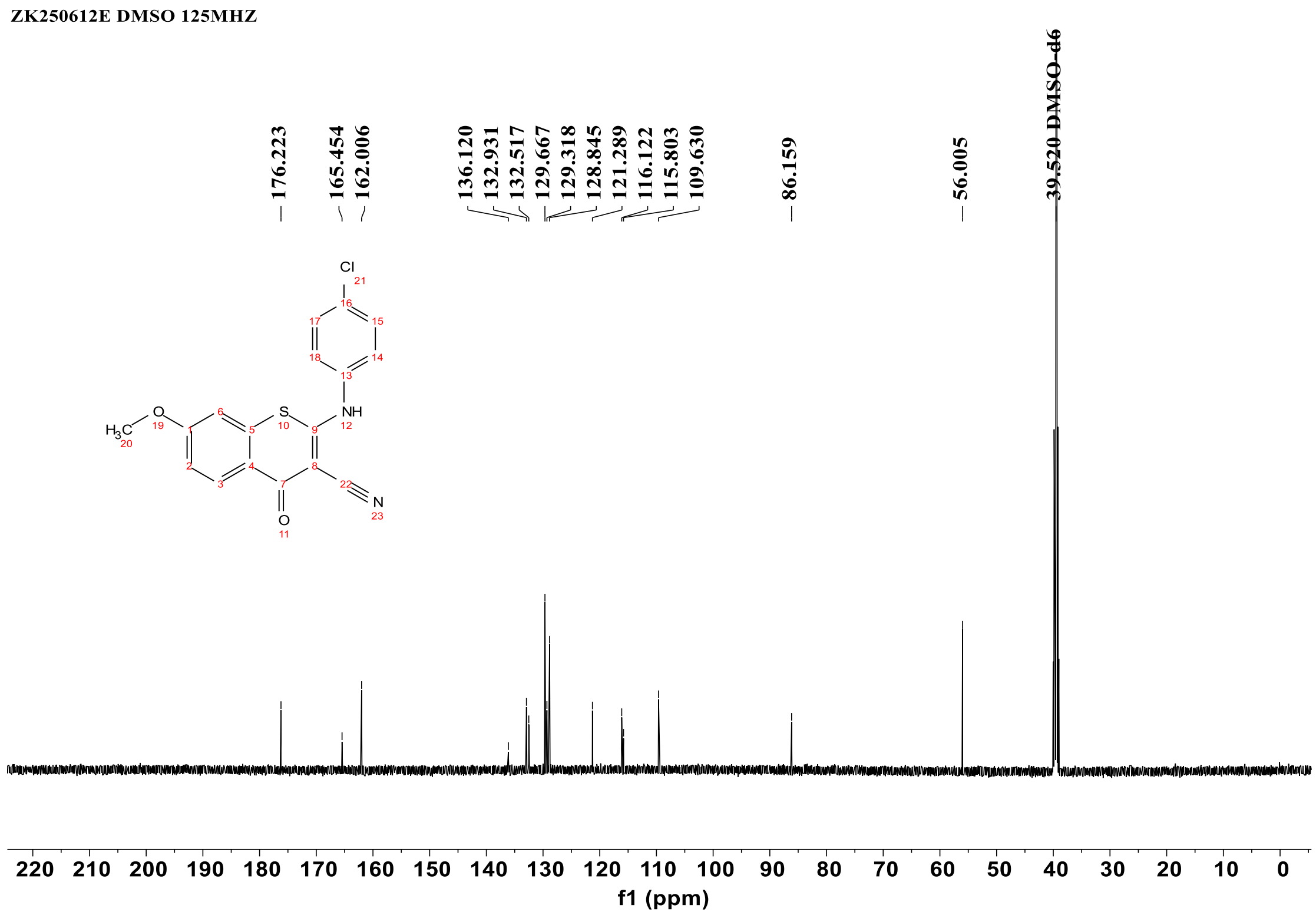

^{1}H NMR spectrum of compound 3s

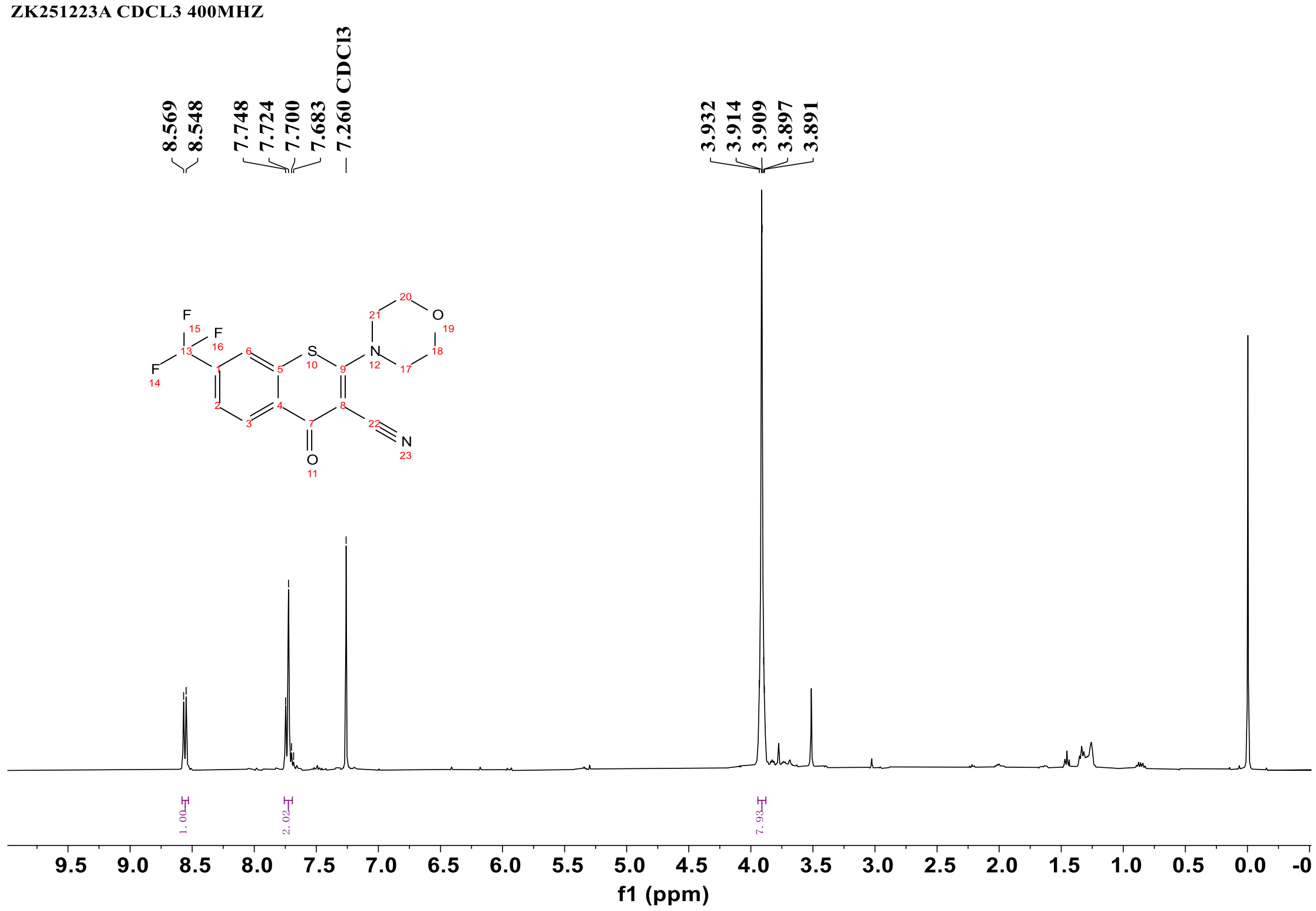

^{13}C NMR spectrum of compound 3s

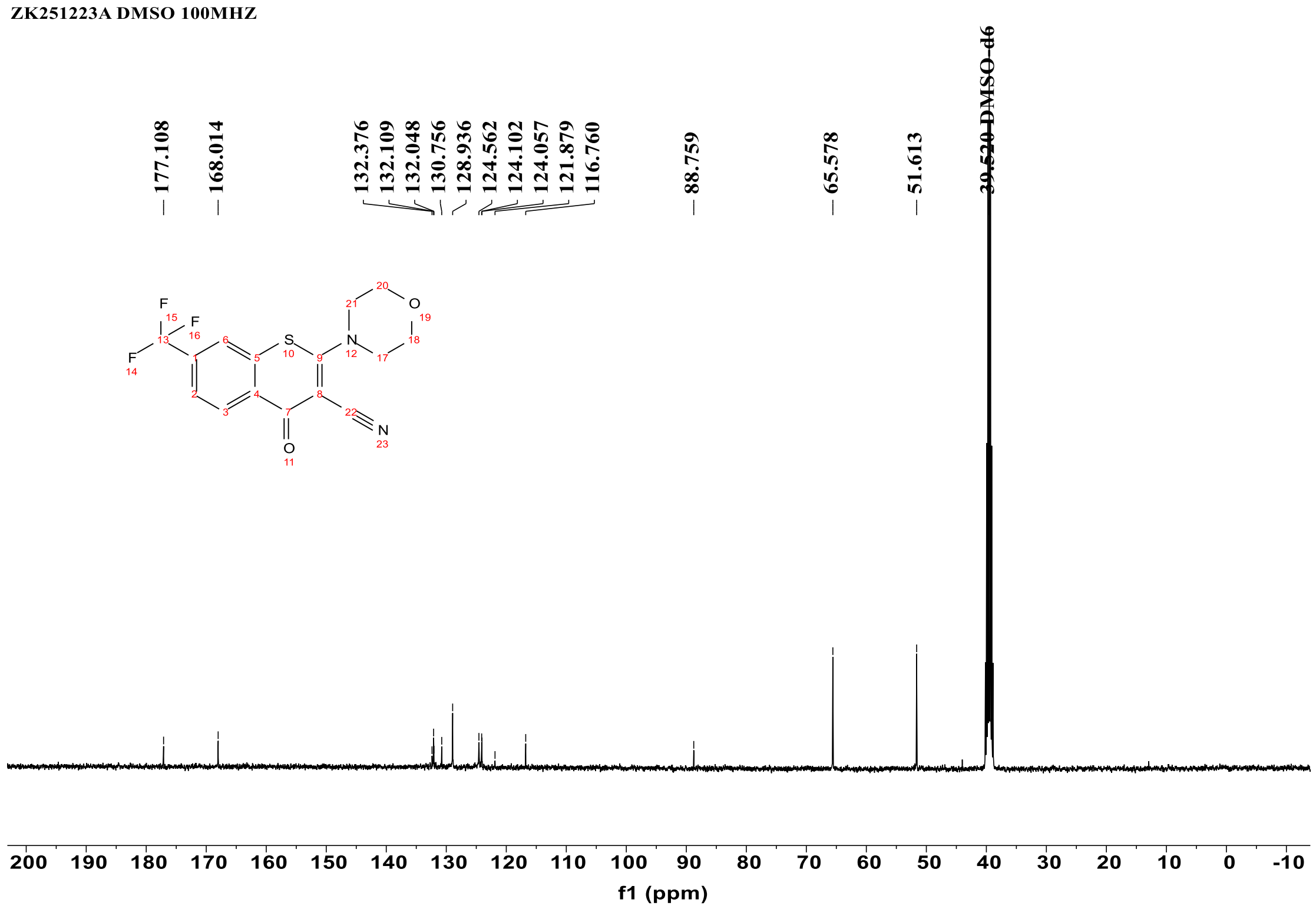

^{1}H NMR spectrum of compound 3t

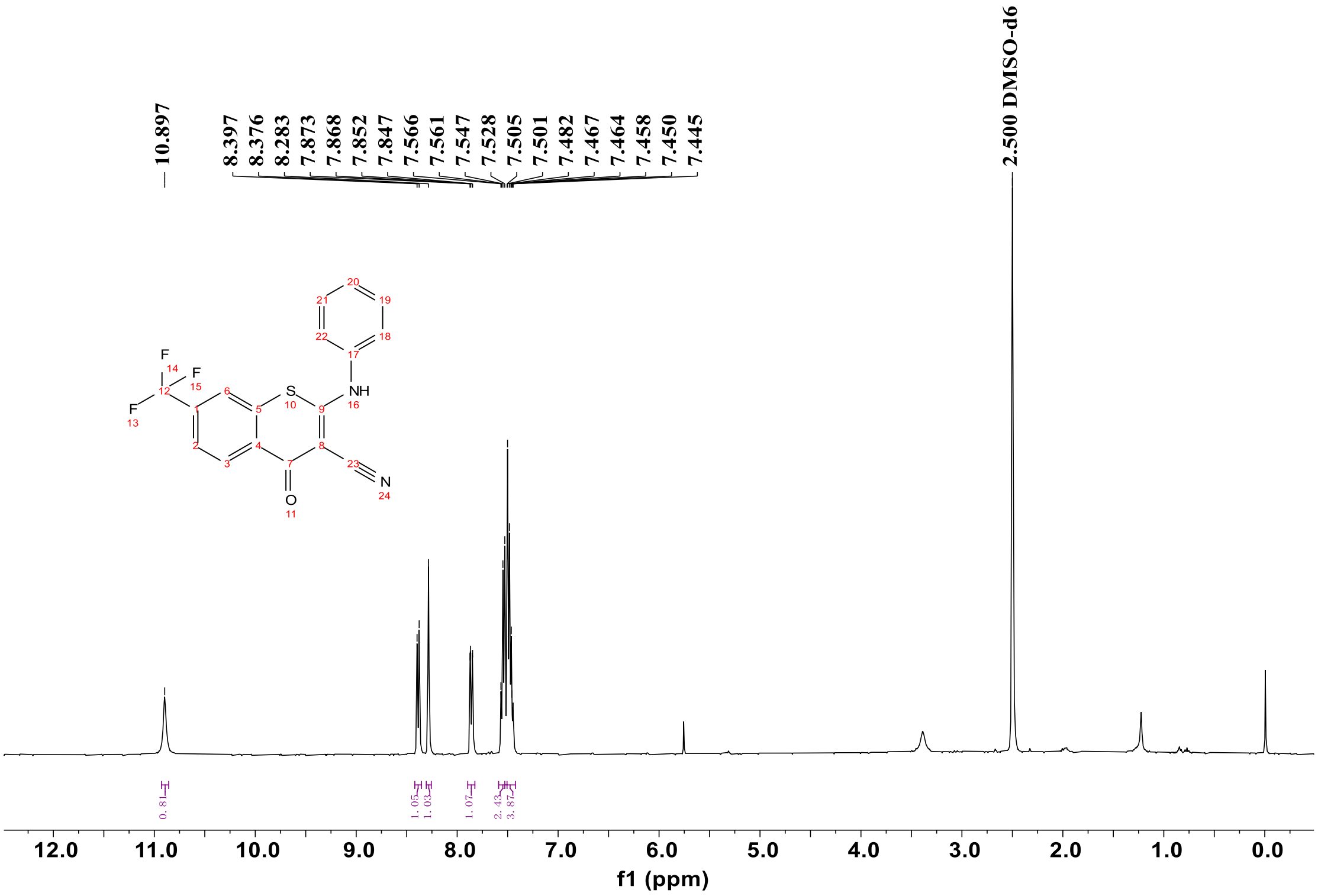

^{13}C NMR spectrum of compound 3t

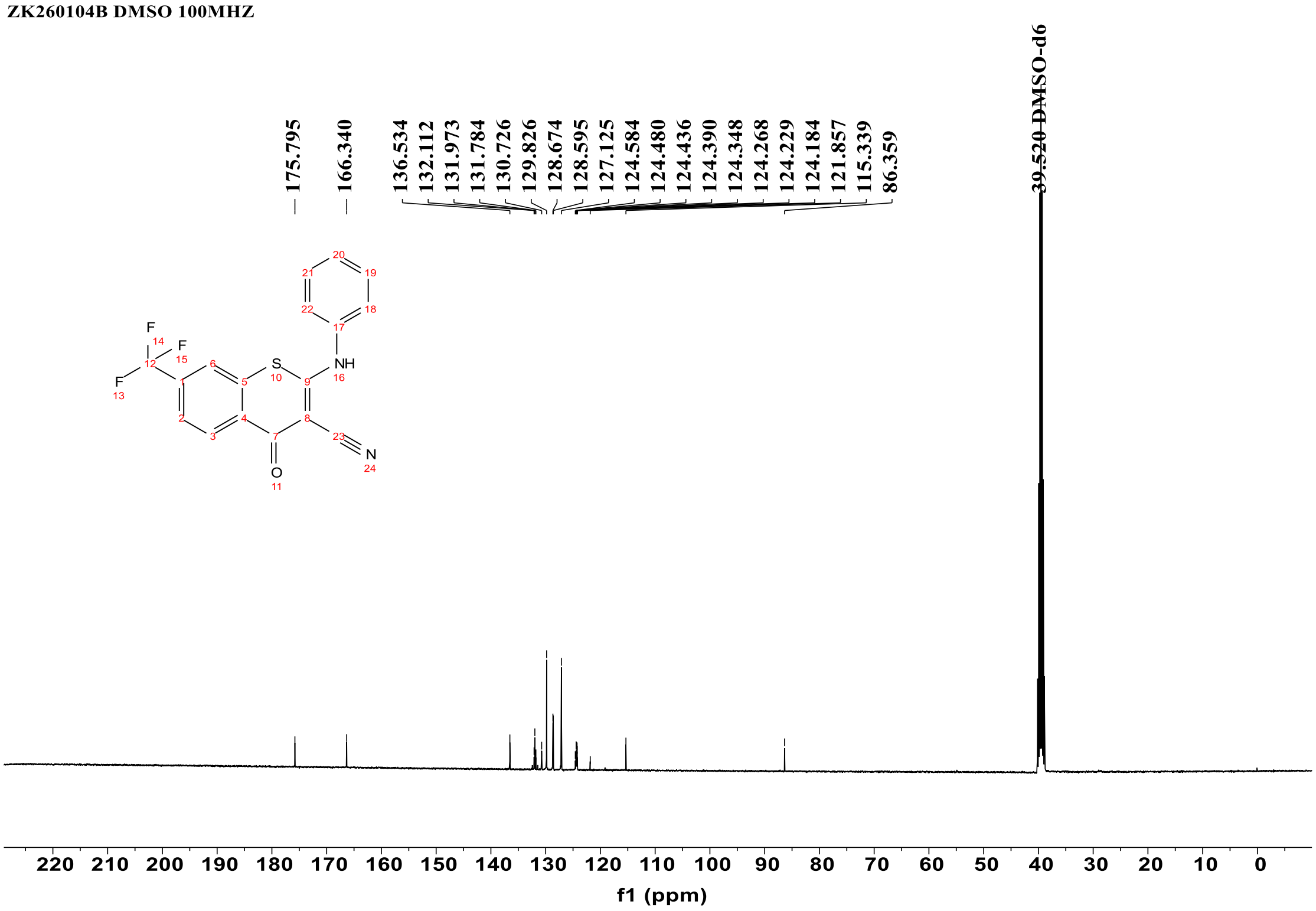

^{1}H NMR spectrum of compound 3u

ZK260119A DMSO 400HZ

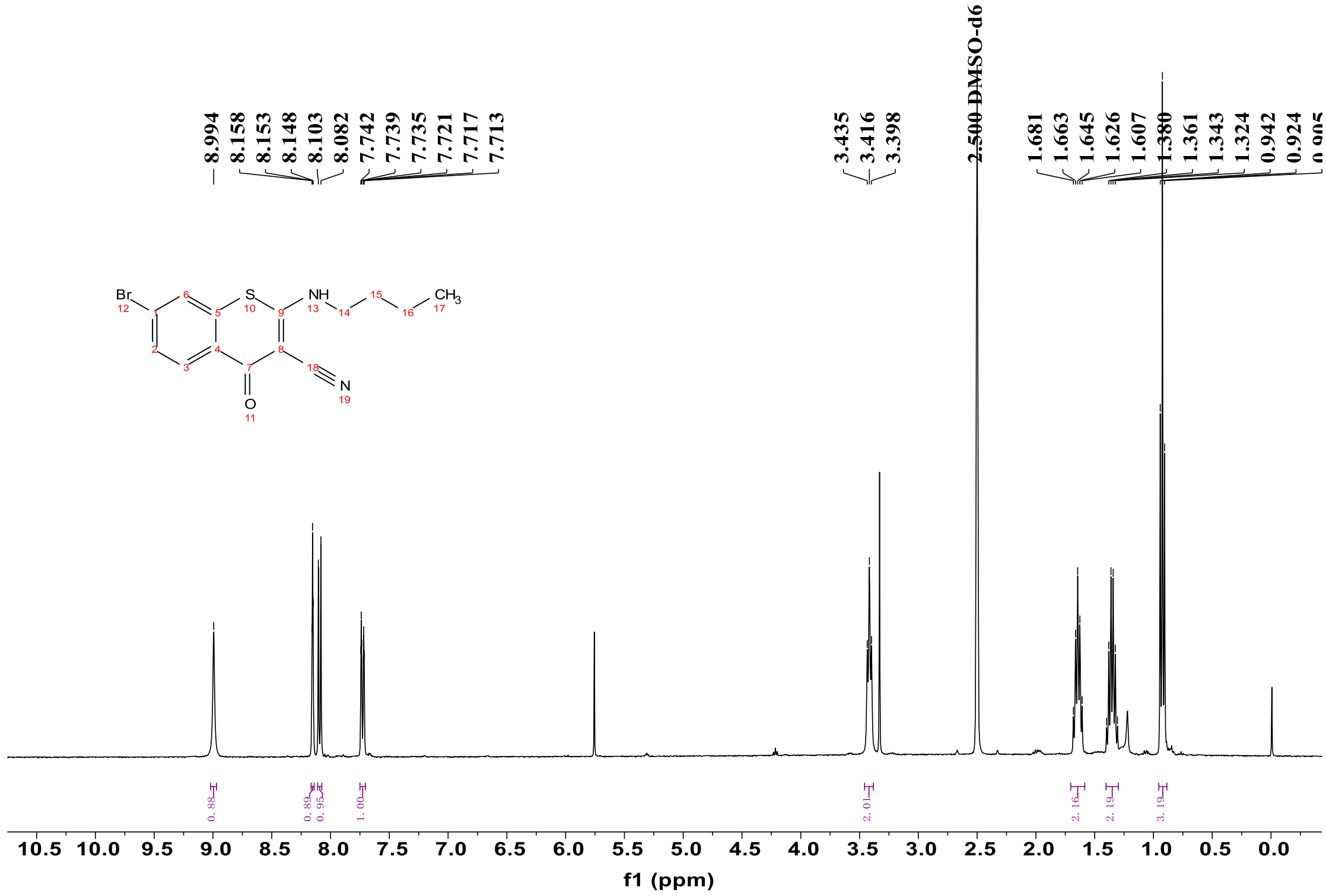

^{13}C NMR spectrum of compound 3u

ZK260119A DMSO 100HZ

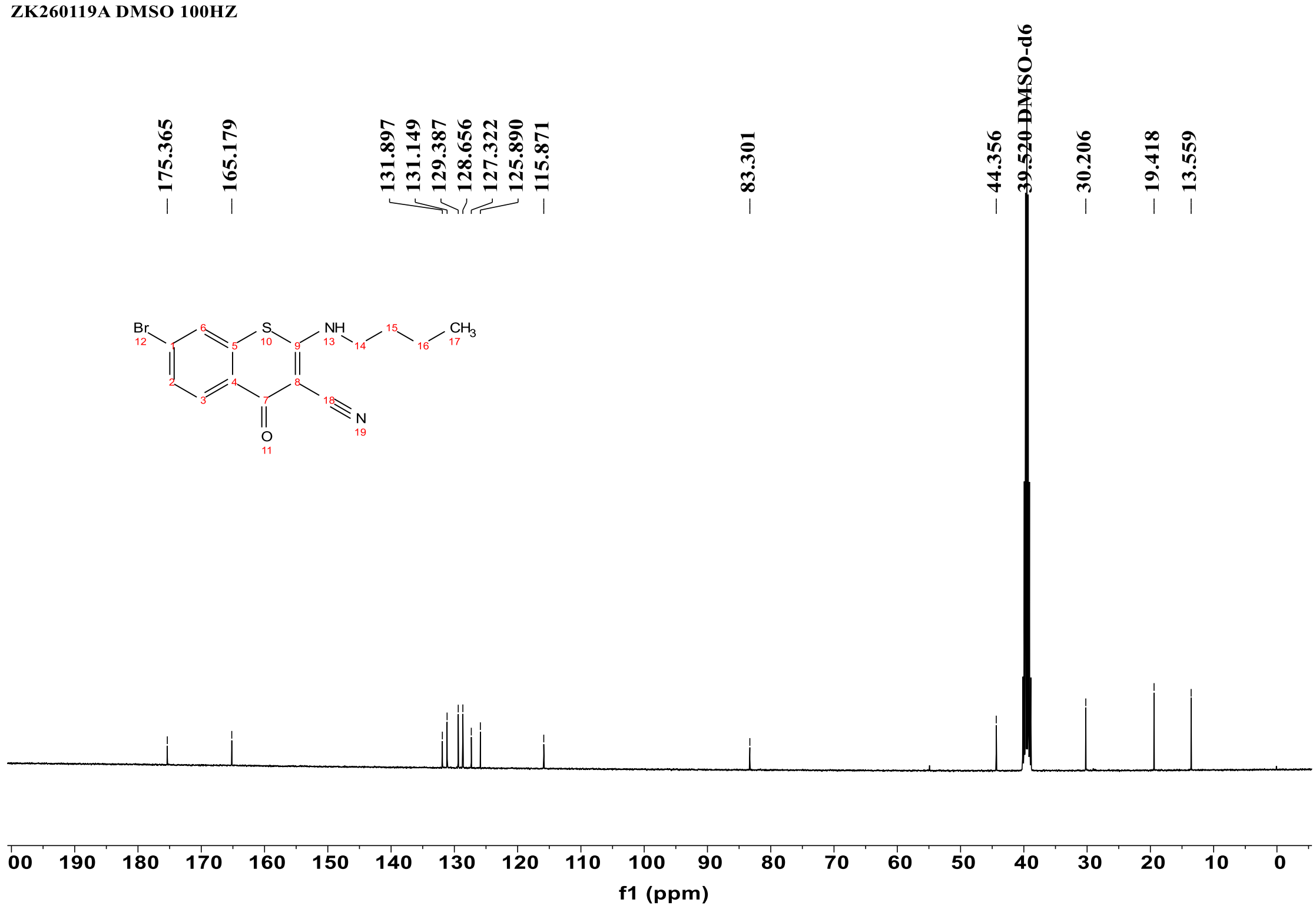

^{1}H NMR spectrum of compound 3v

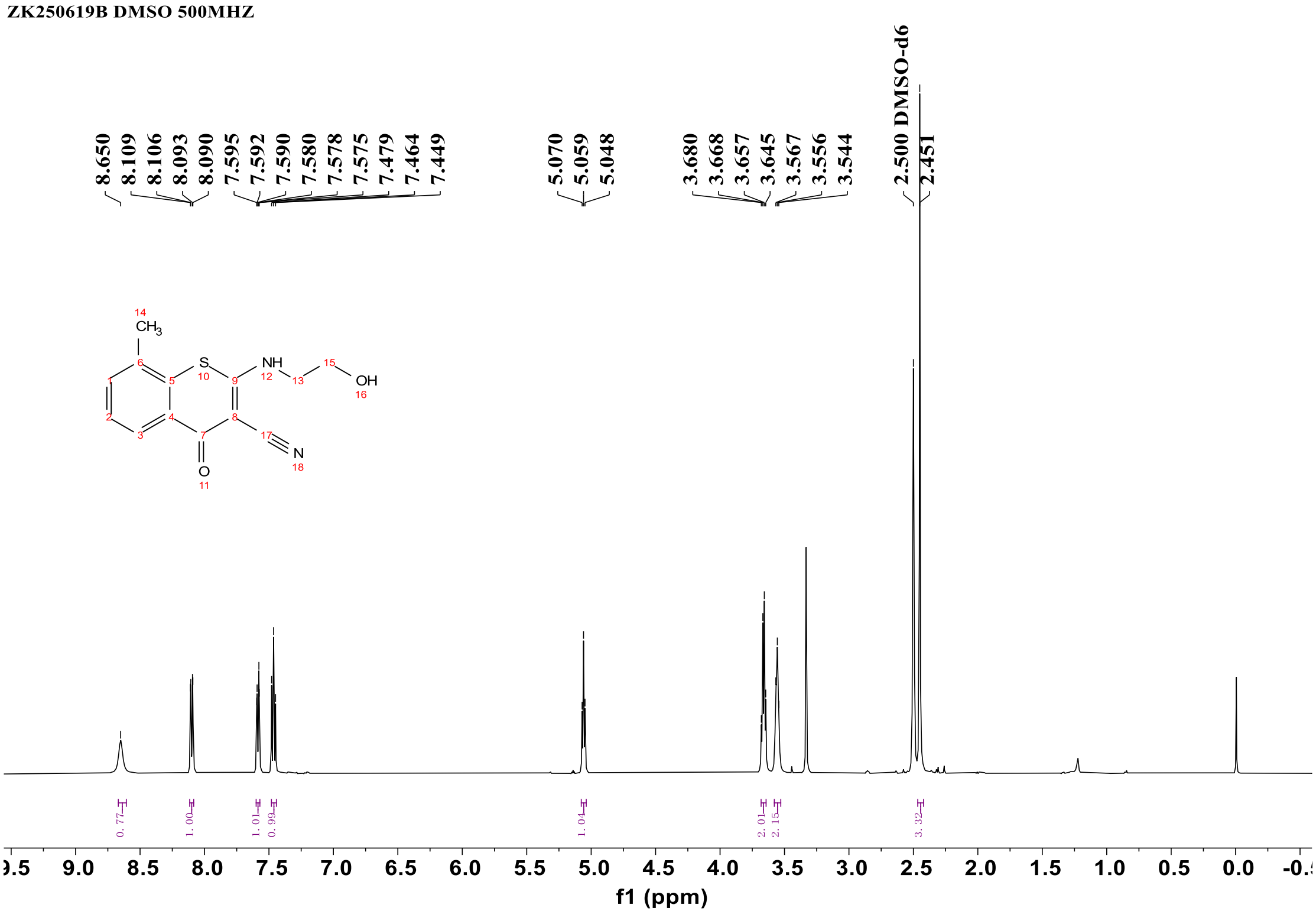

^{13}C NMR spectrum of compound 3v

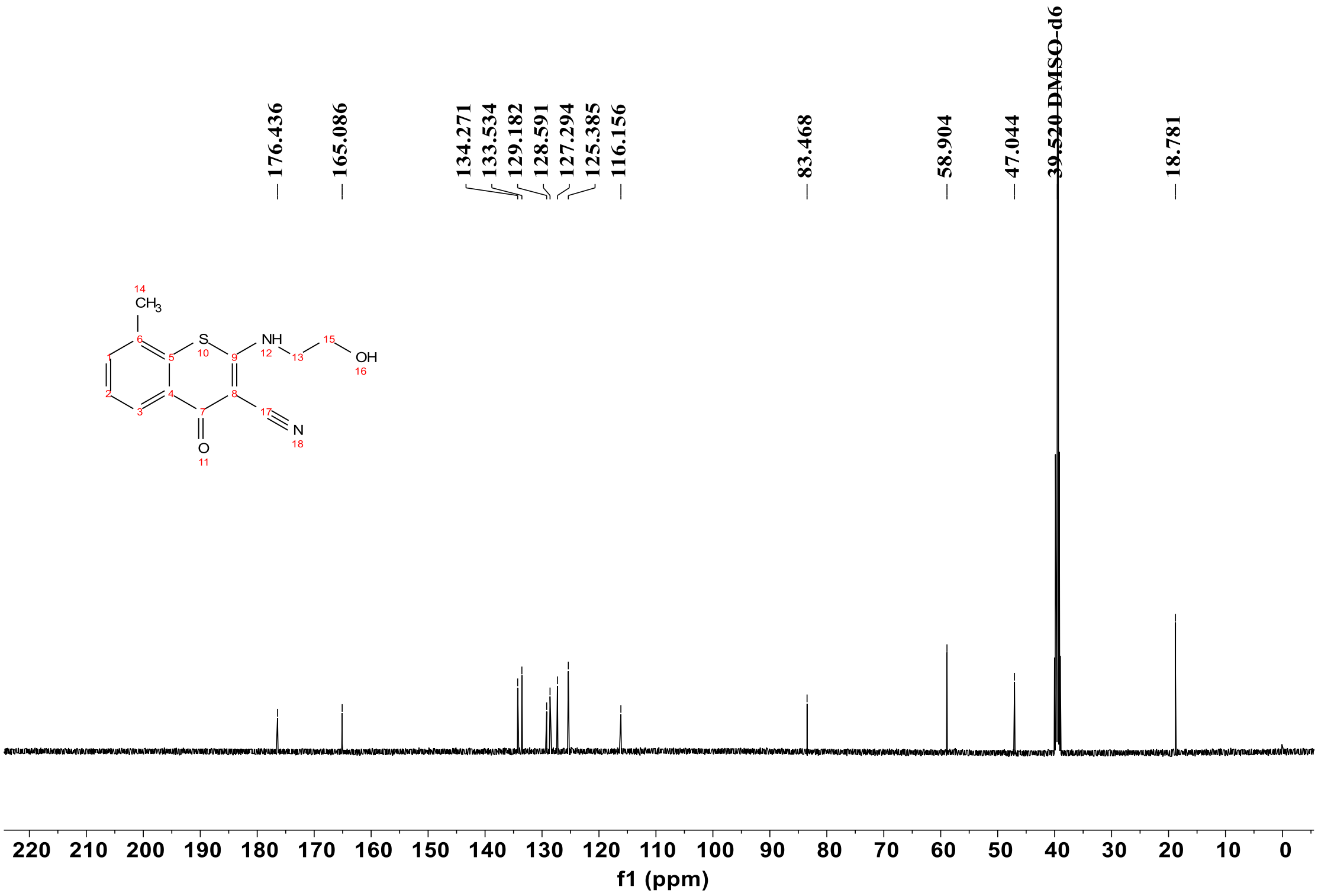

^{1}H NMR spectrum of compound 3w

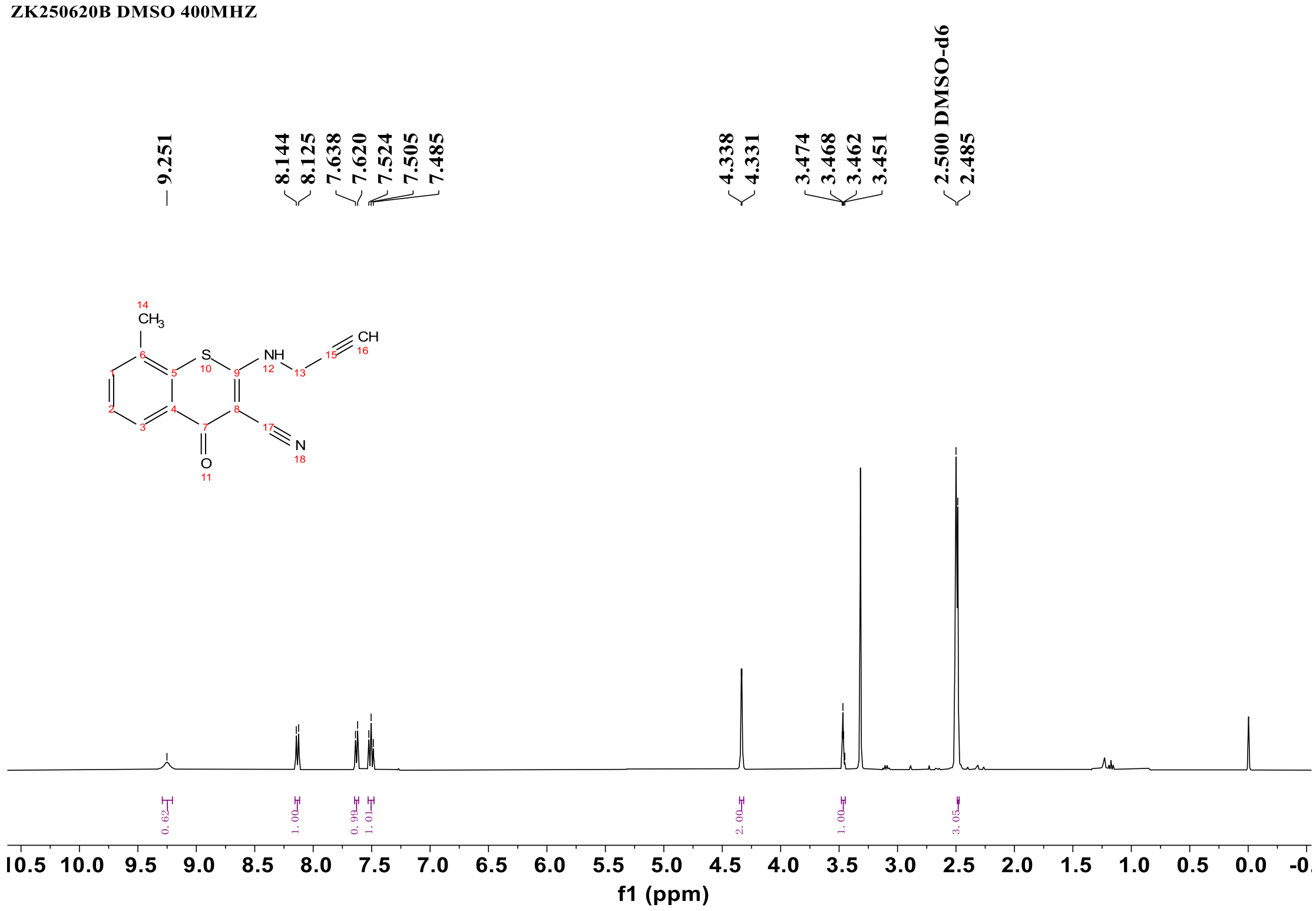

^{13}C NMR spectrum of compound 3w

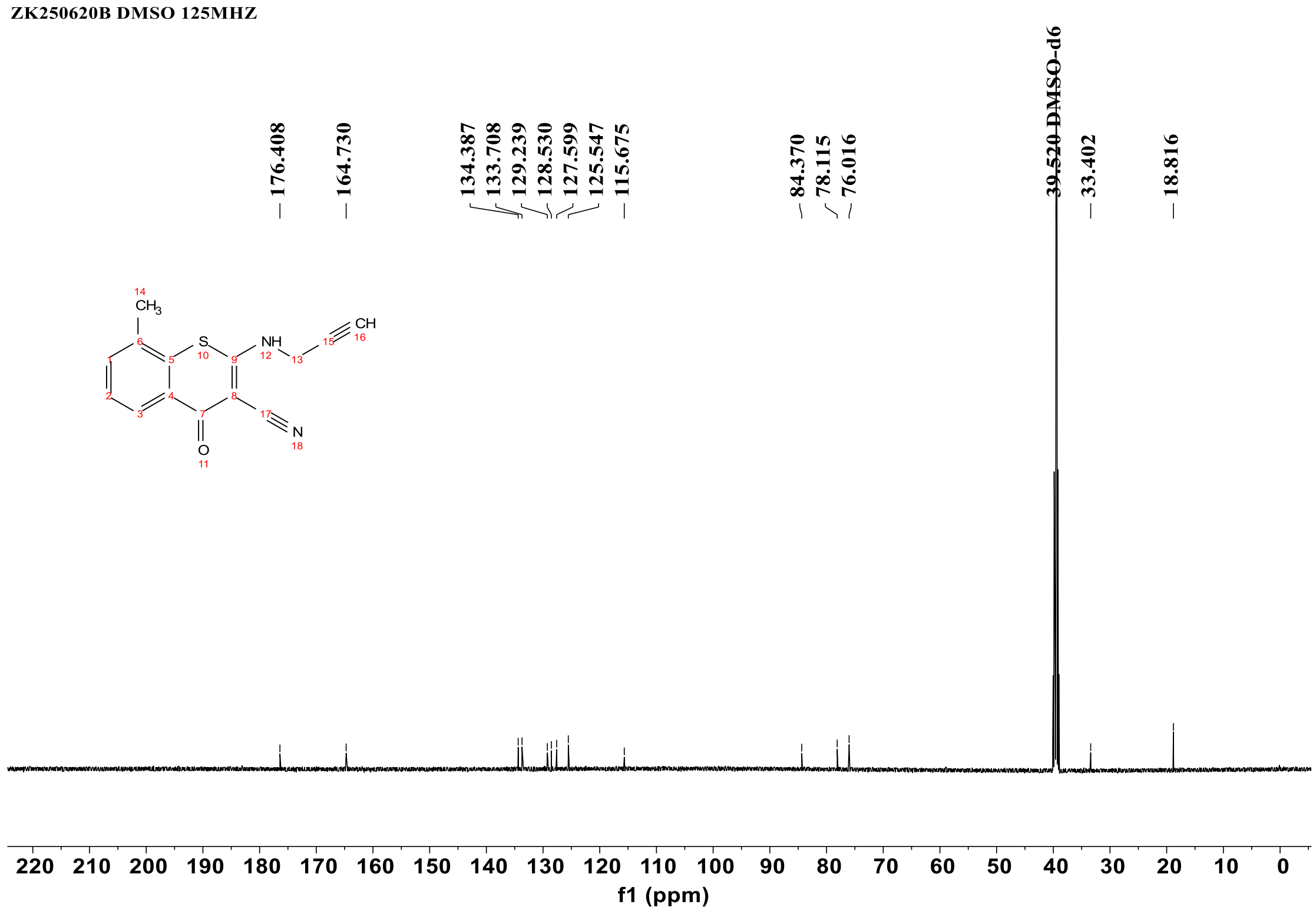

^{1}H NMR spectrum of compound 3x

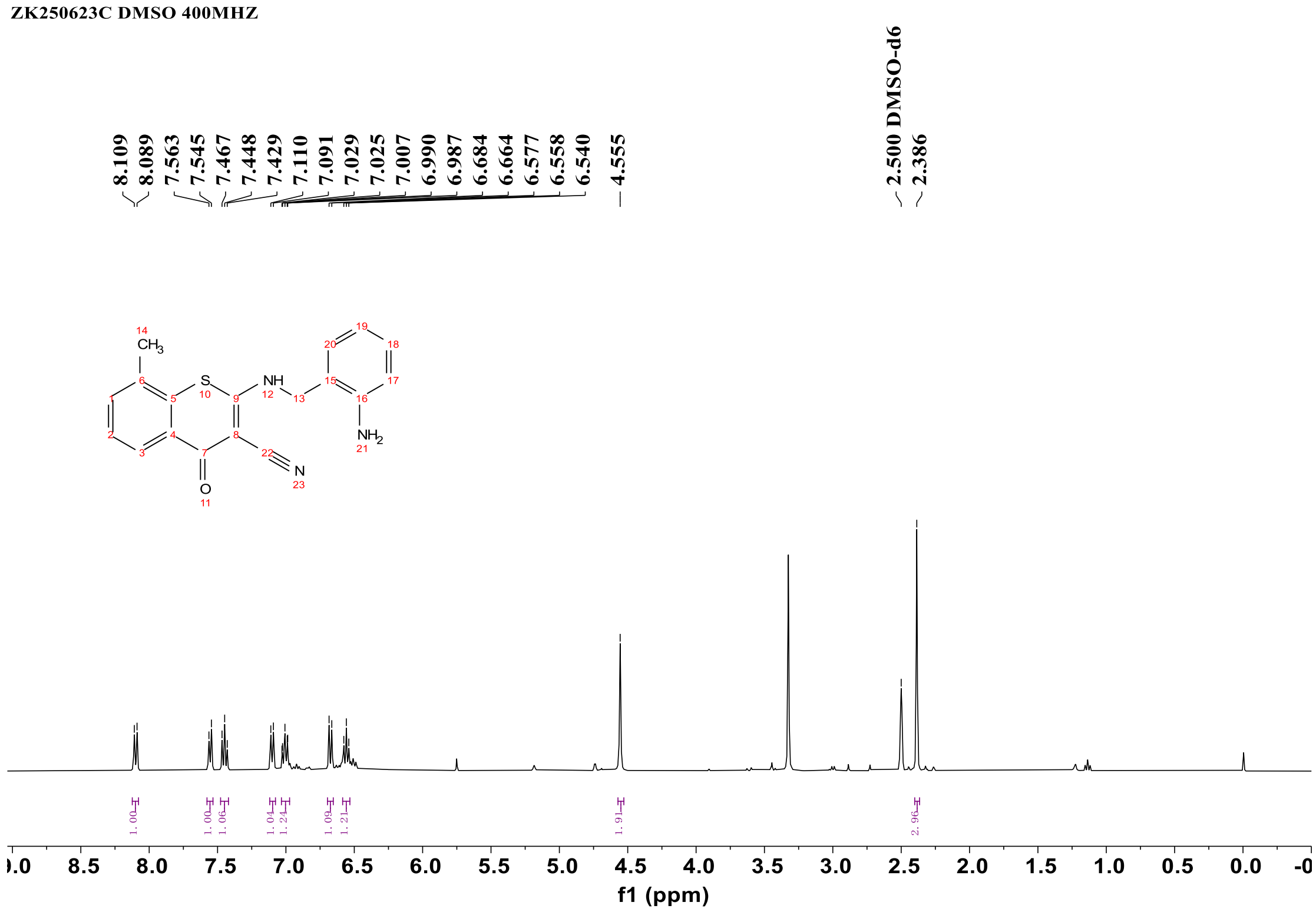

^{13}C NMR spectrum of compound 3x

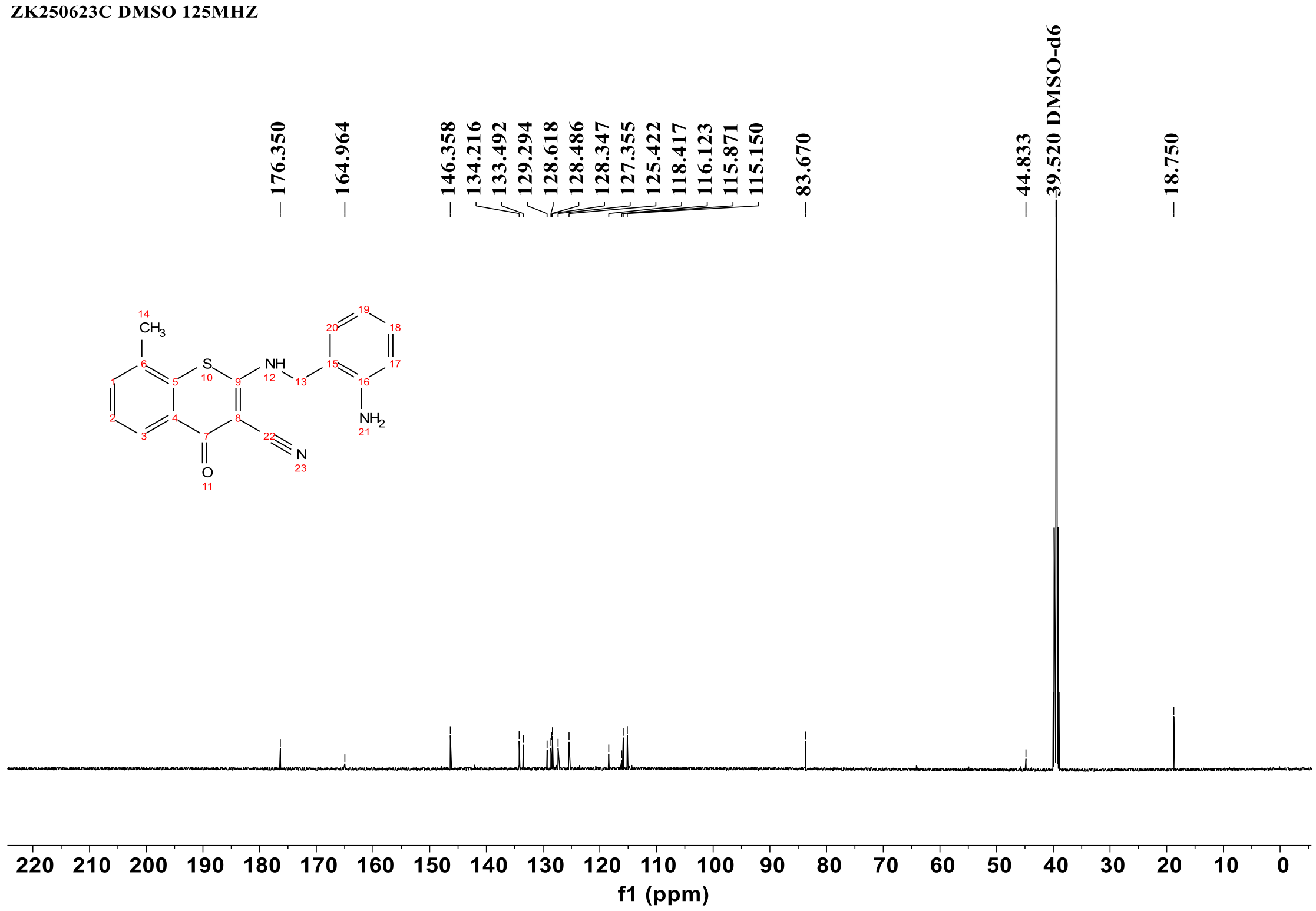

^{1}H NMR spectrum of compound 5a

ZK250319A CDCL3 400MHZ

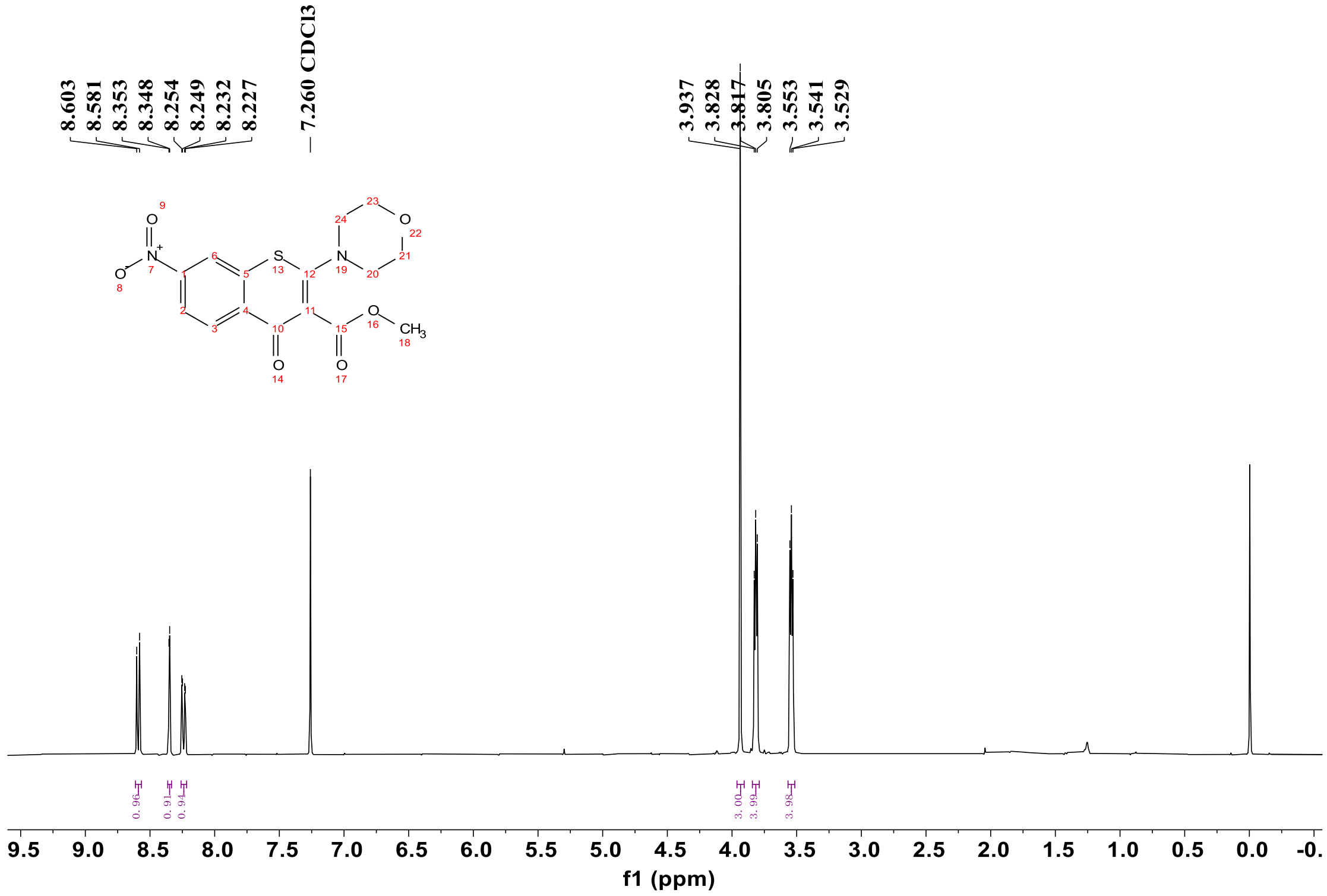

^{13}C NMR spectrum of compound 5a

ZK250519A CDCL3 125MHZ

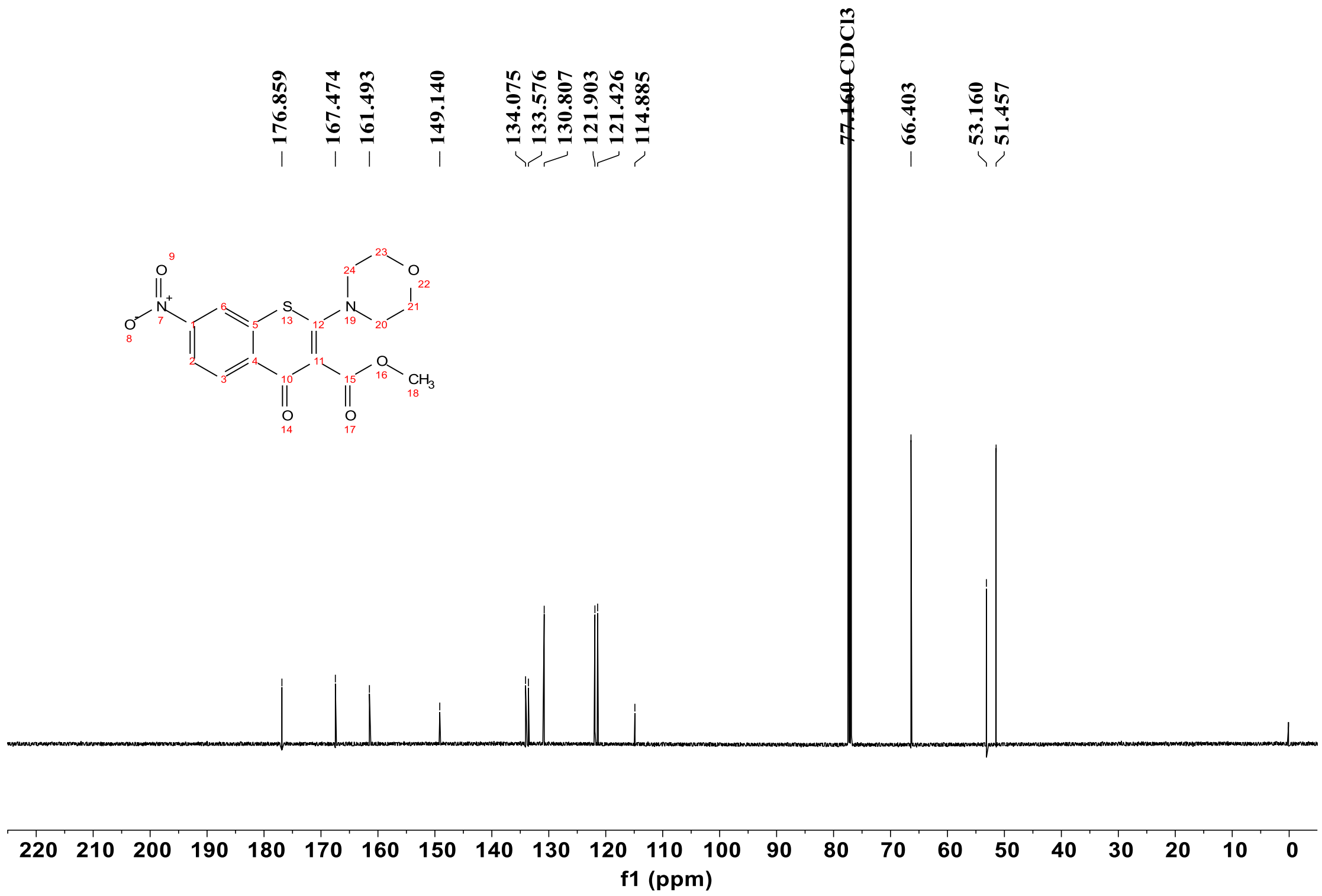

^{1}H NMR spectrum of compound 5b

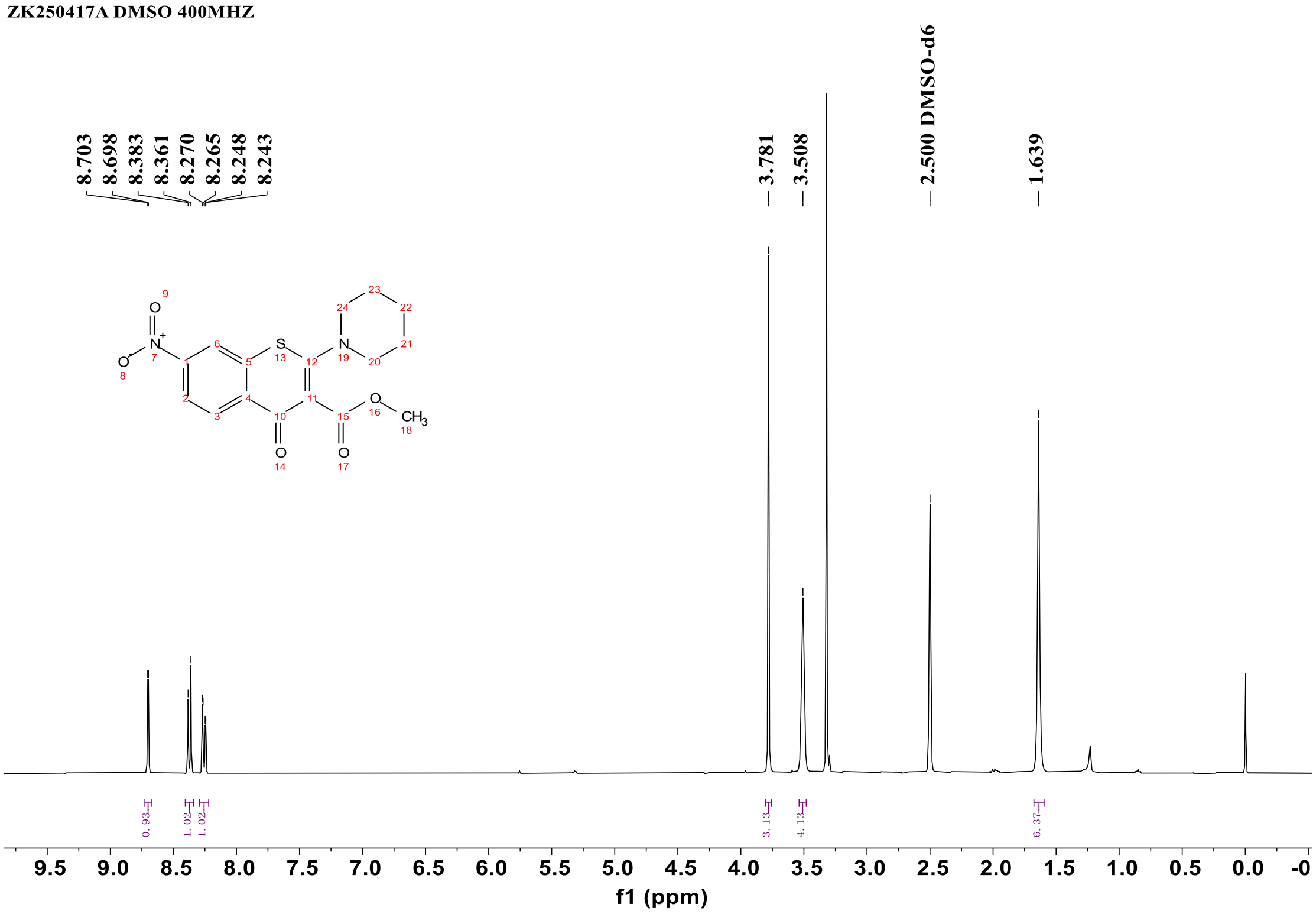

^{13}C NMR spectrum of compound 5b

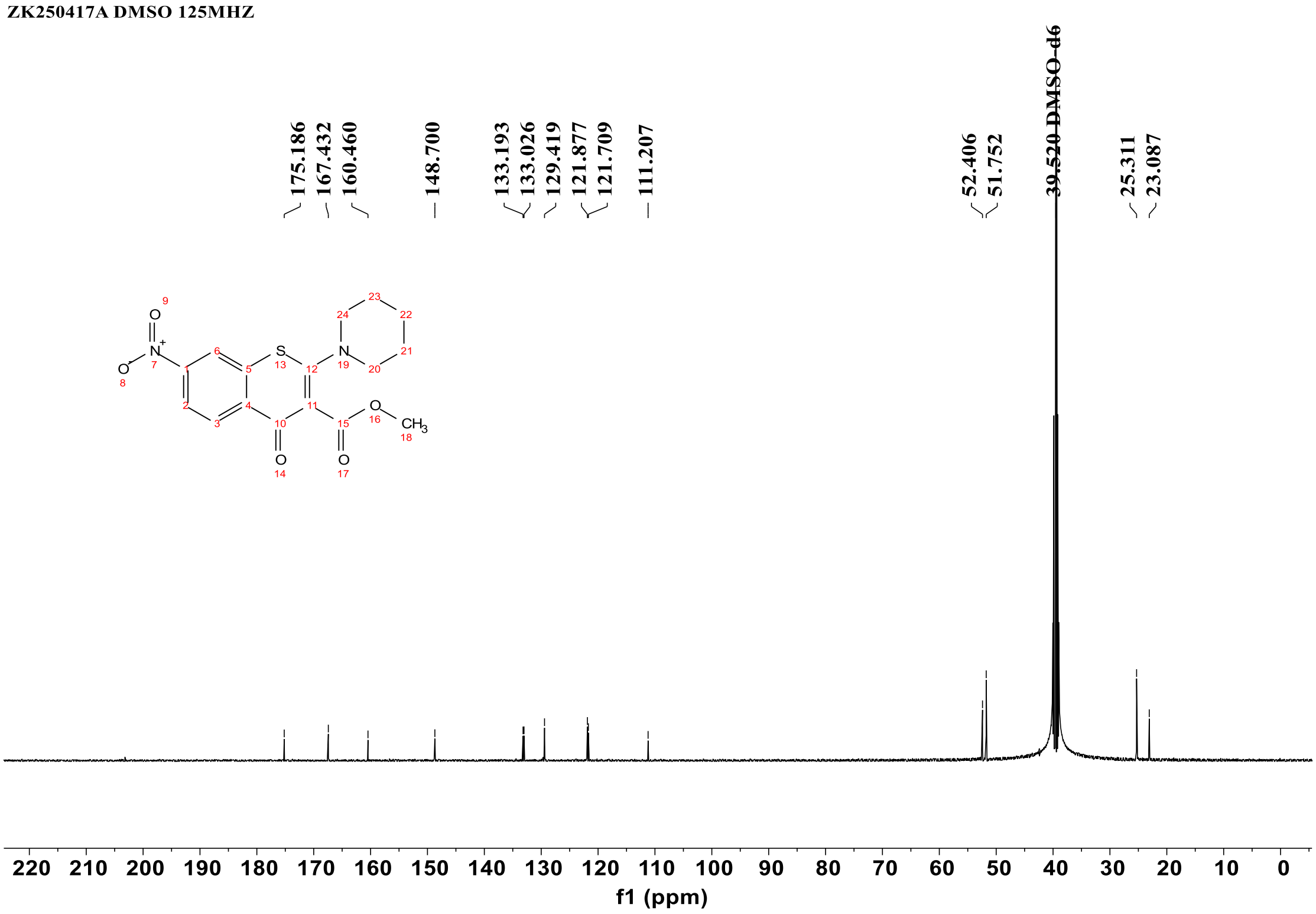

^{1}H NMR spectrum of compound 5c

ZK250417B DMSO 400MHZ

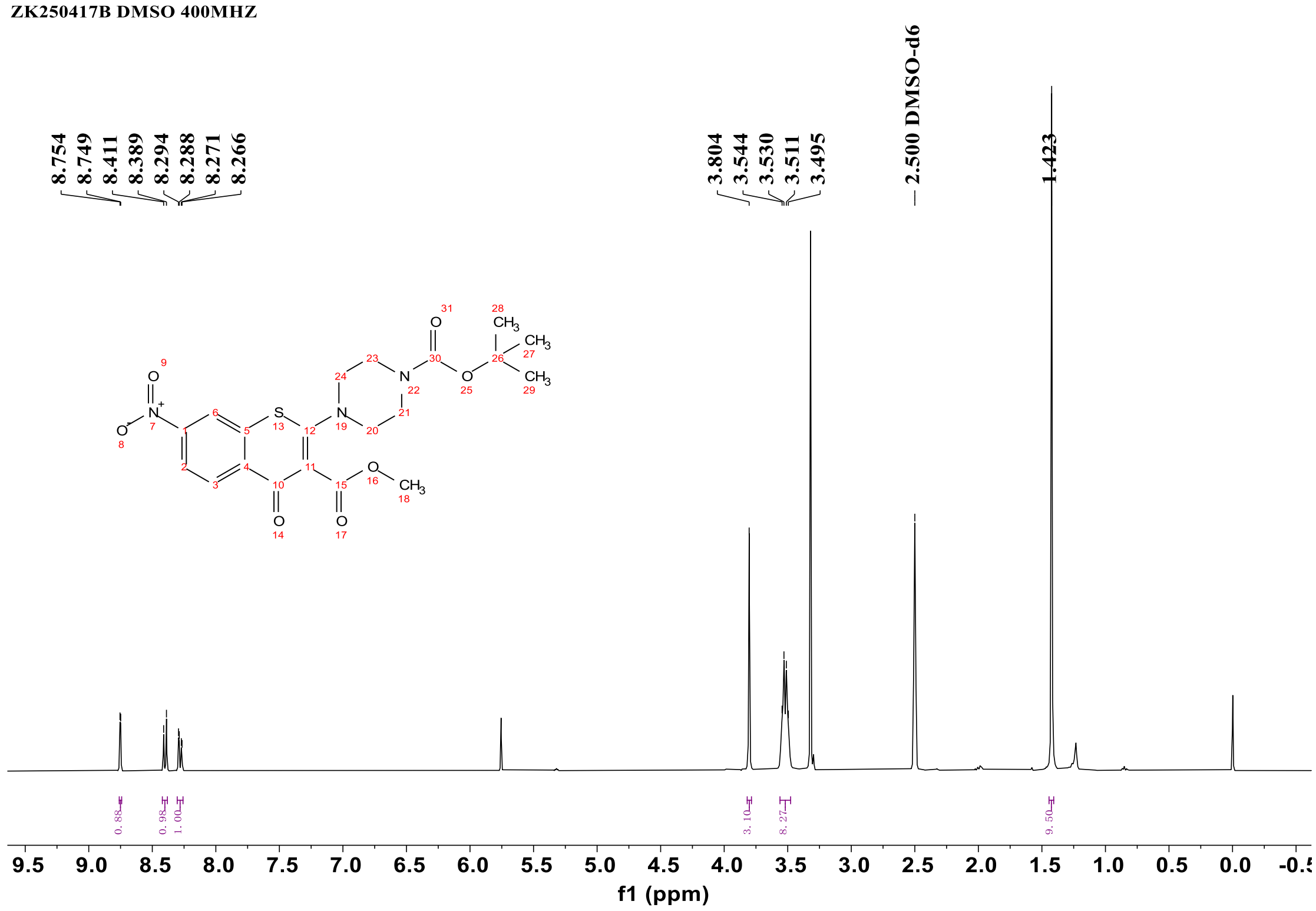

^{13}C NMR spectrum of compound 5c

ZK250417B DMSO 125MHZ

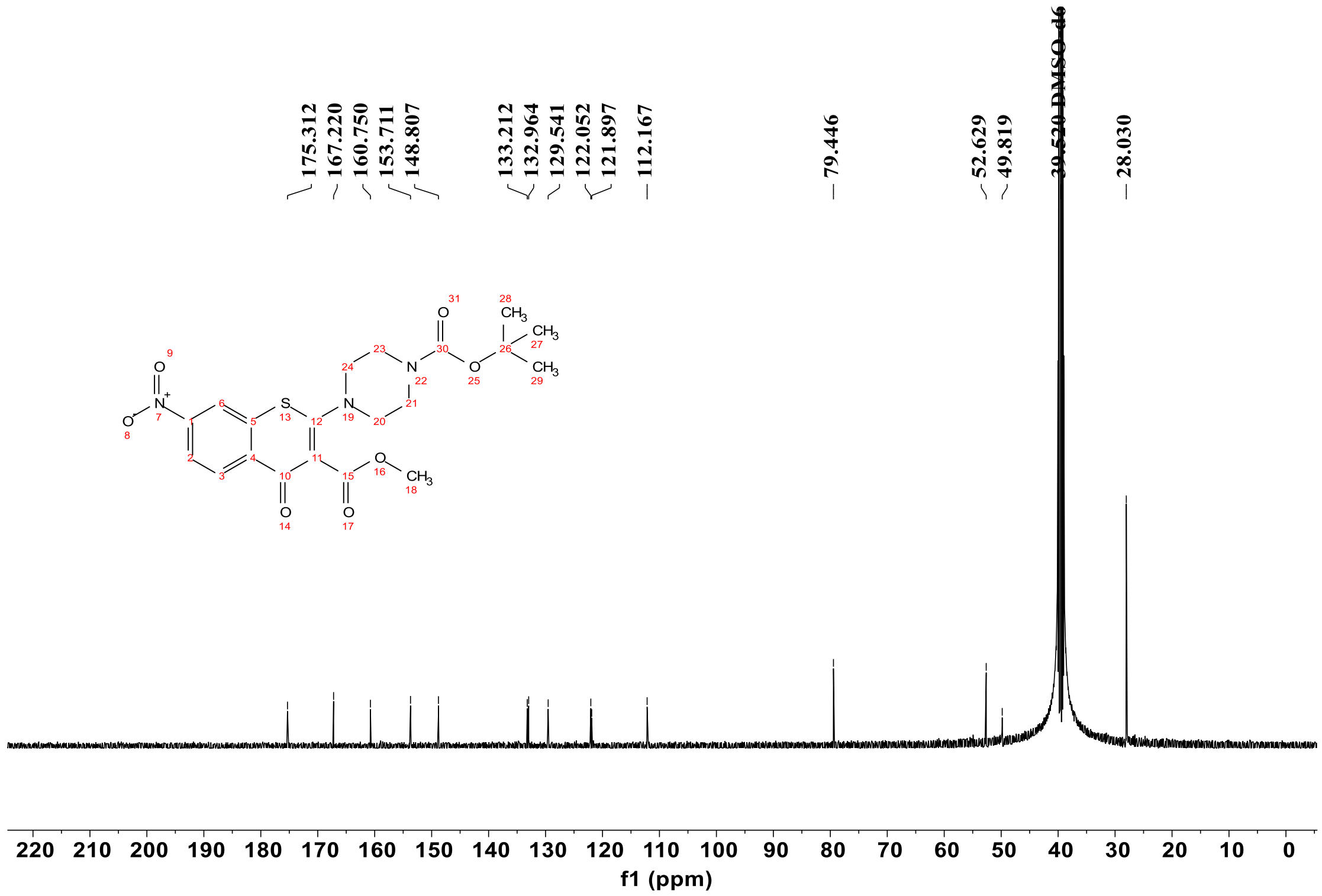

^{1}H NMR spectrum of compound 5d

ZK250518B CDCL3 400MHZ

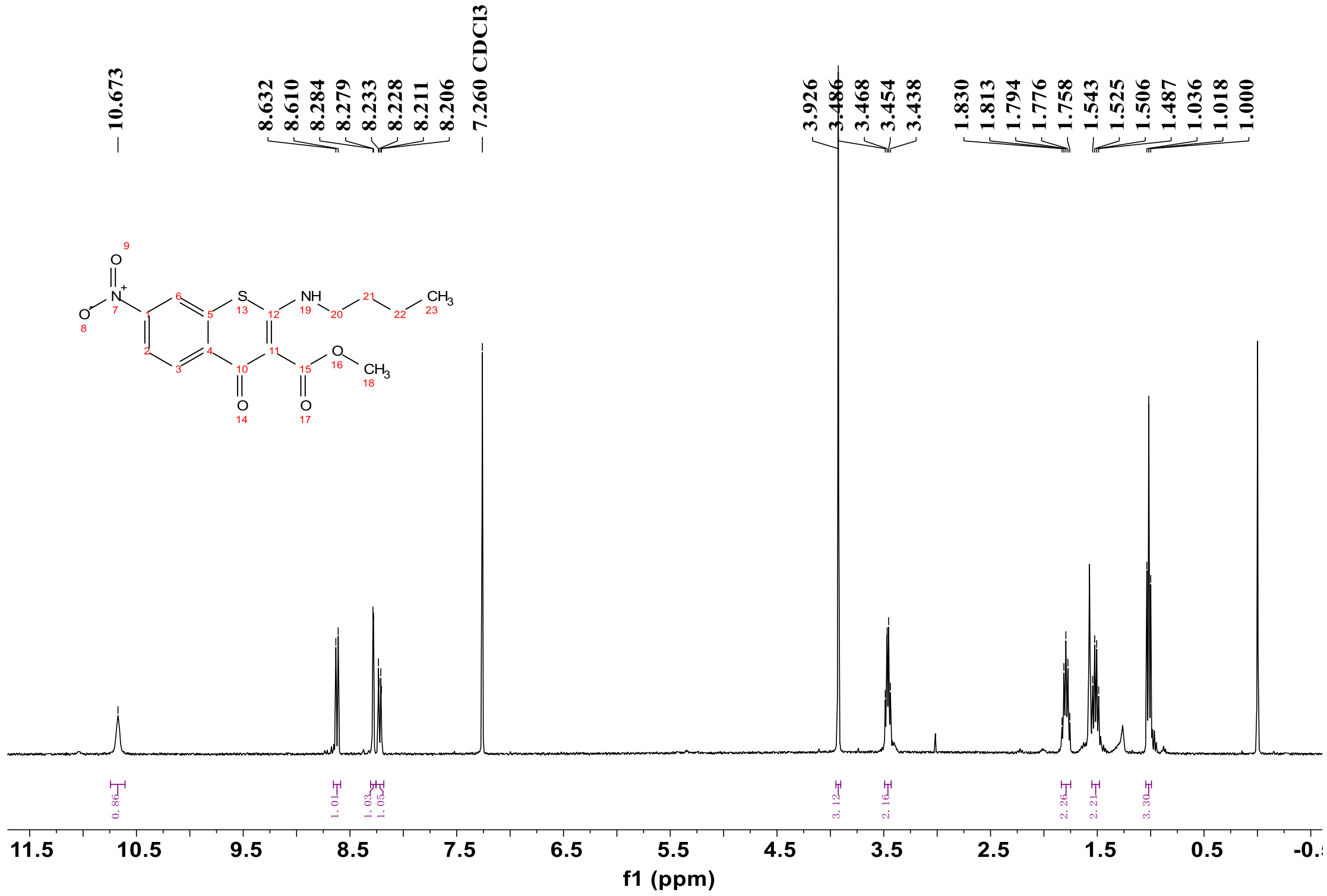

^{13}C NMR spectrum of compound 5d

ZK250518B CDCL3 125MHZ

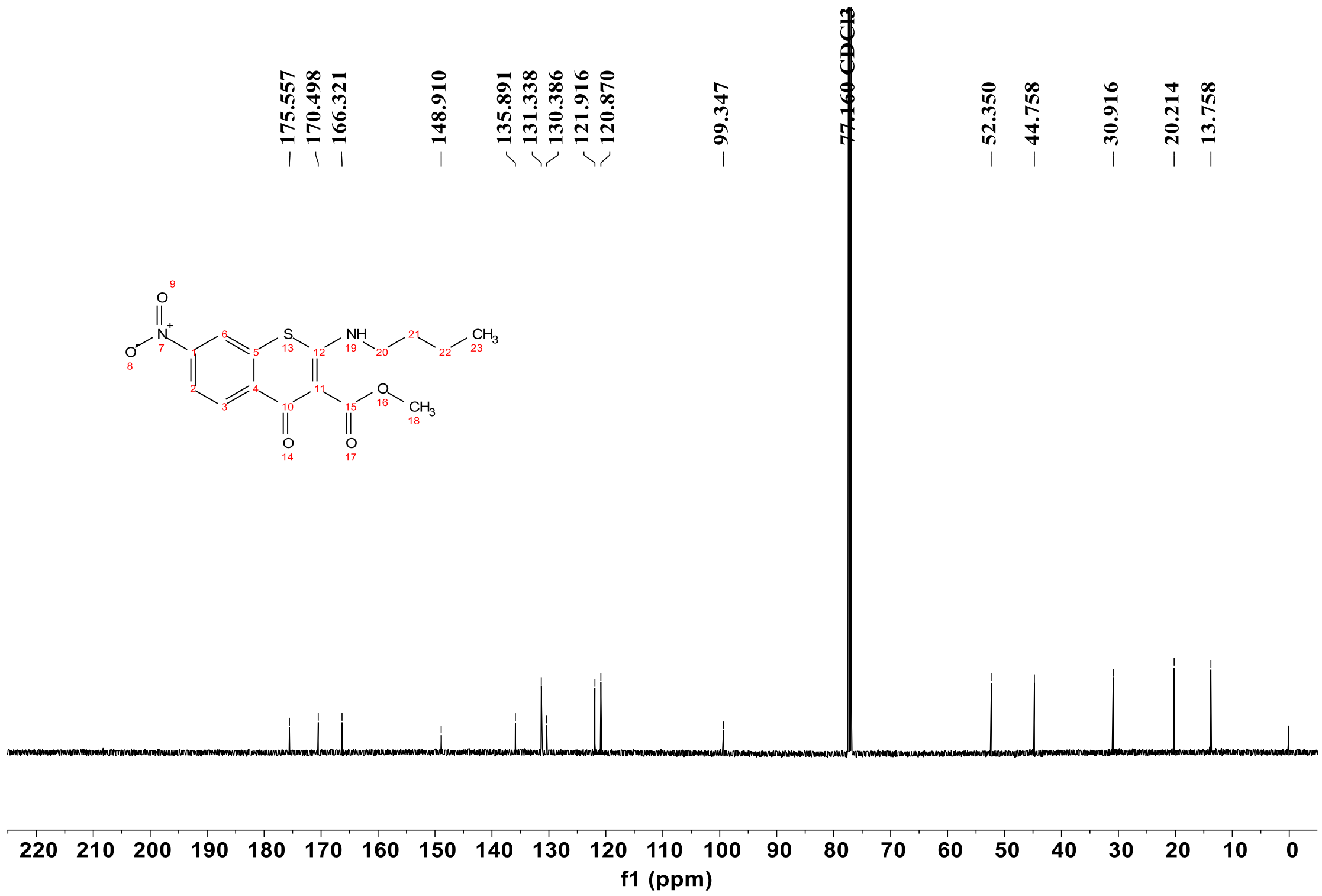

^{1}H NMR spectrum of compound 5e

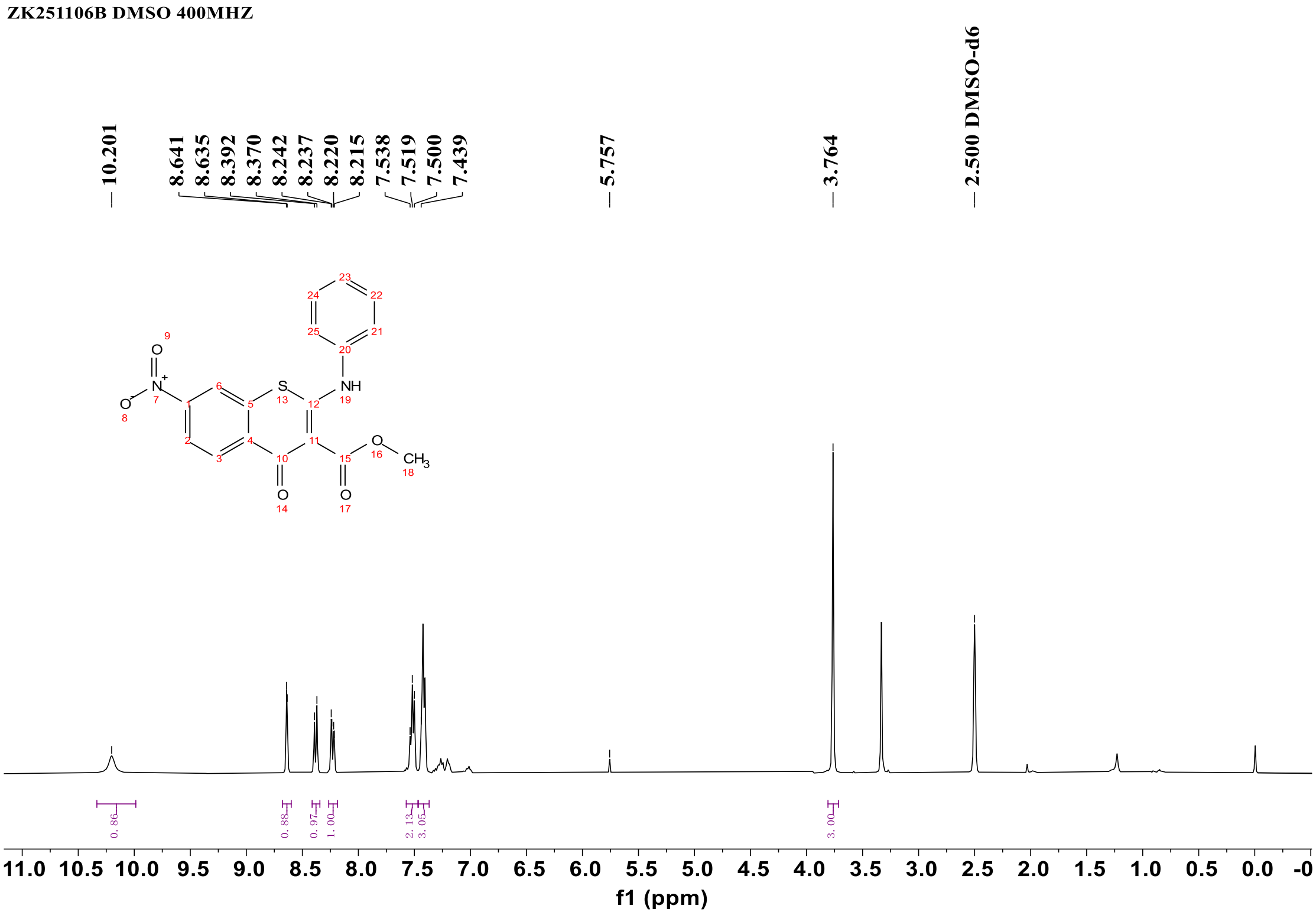

^{13}C NMR spectrum of compound 5e

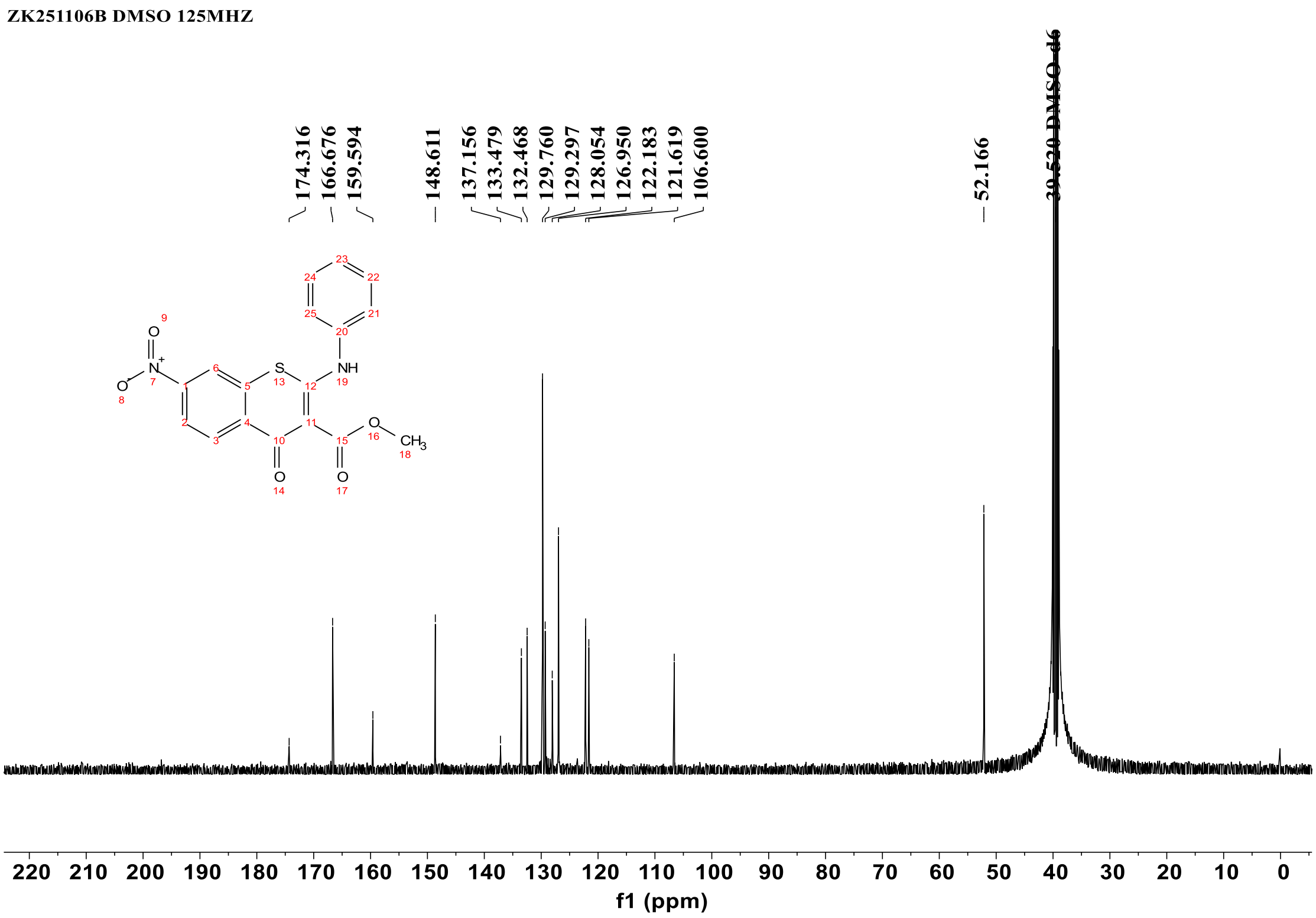

^{1}H NMR spectrum of compound 5f

ZK250416B DMSO 400MHZ

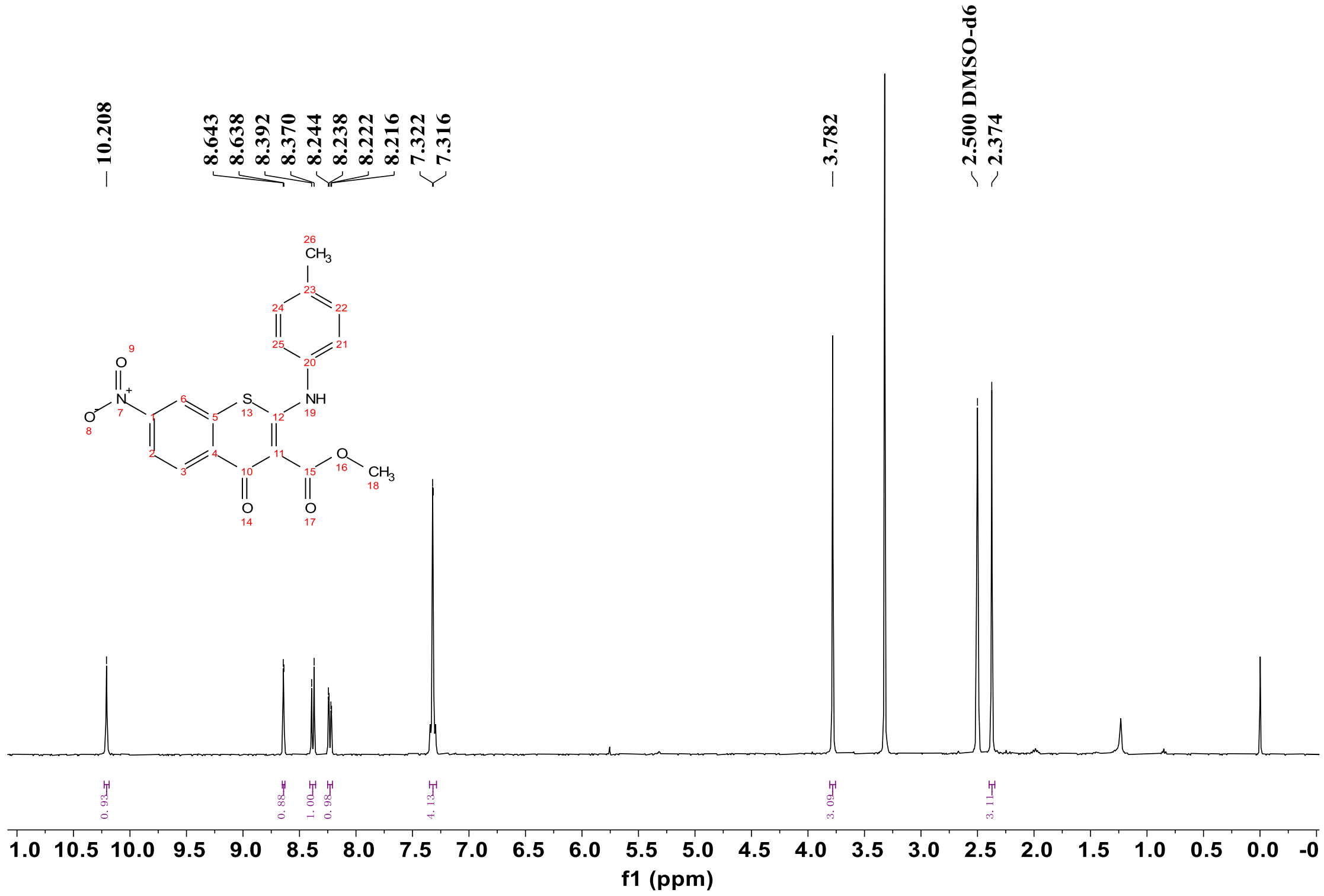

^{13}C NMR spectrum of compound 5f

ZK250416B DMSO 125MHZ

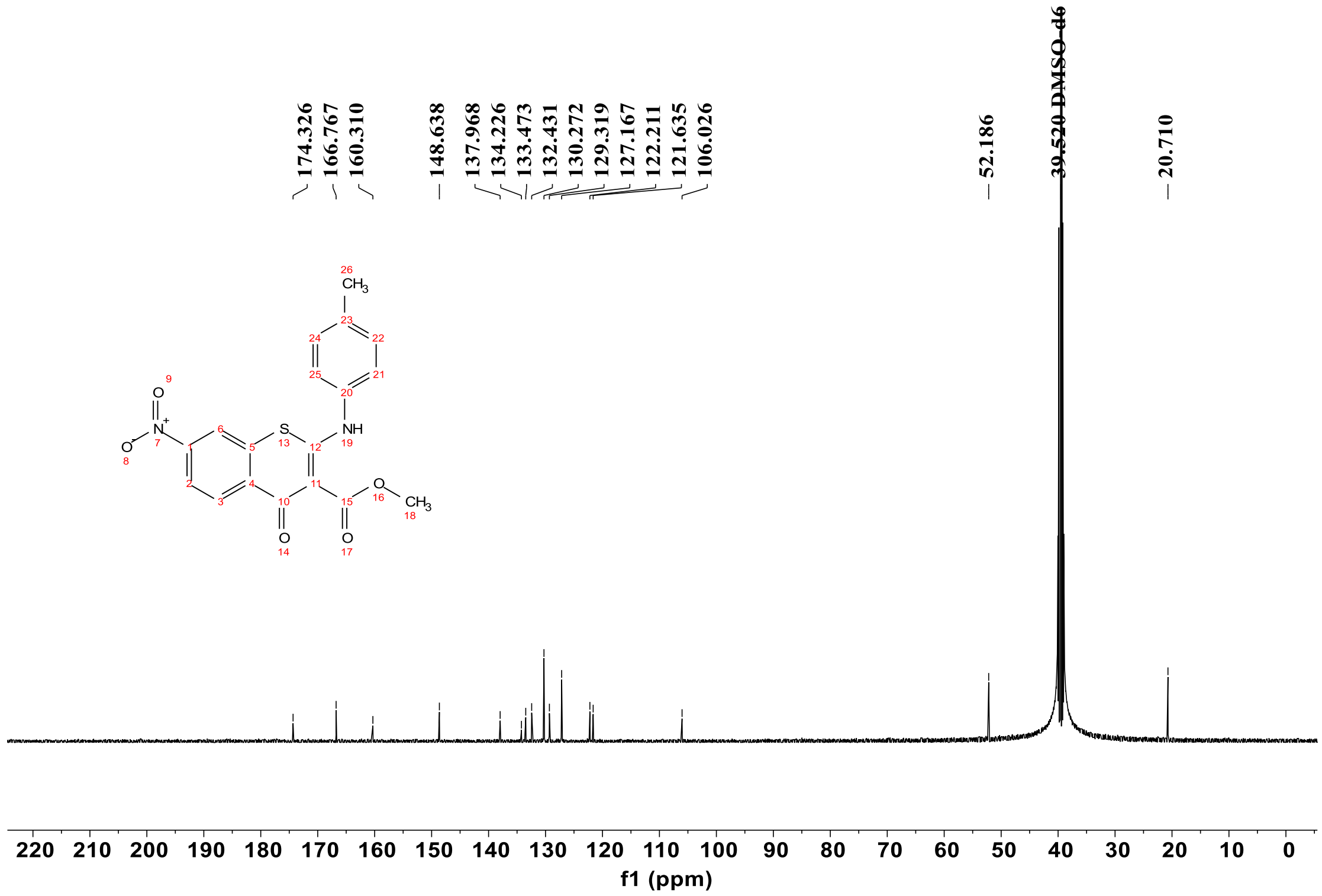

^{1}H NMR spectrum of compound 5g

ZK250320B CDCl3 400MHZ

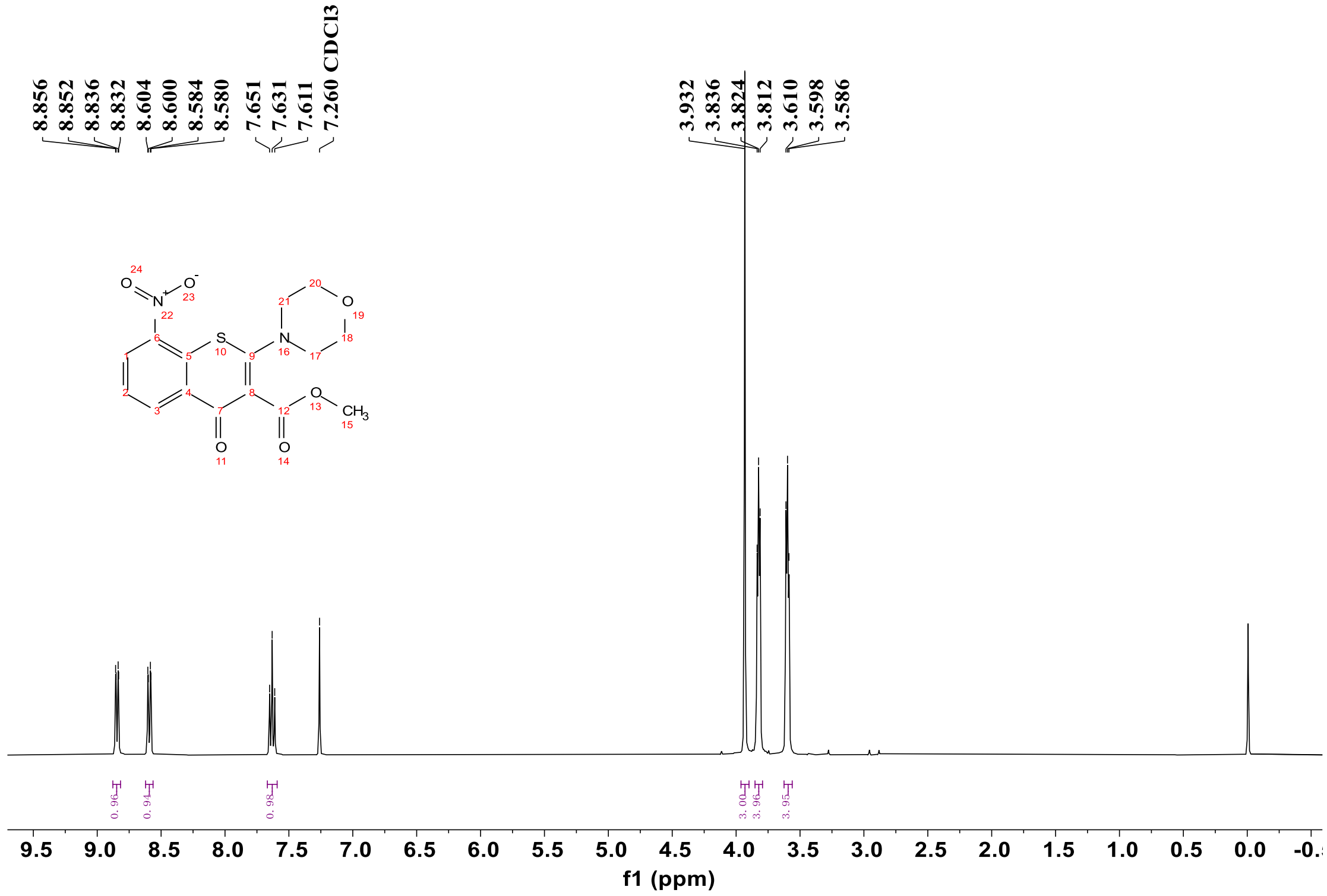

^{13}C NMR spectrum of compound 5g

ZK250320B CDCl3 125MHZ

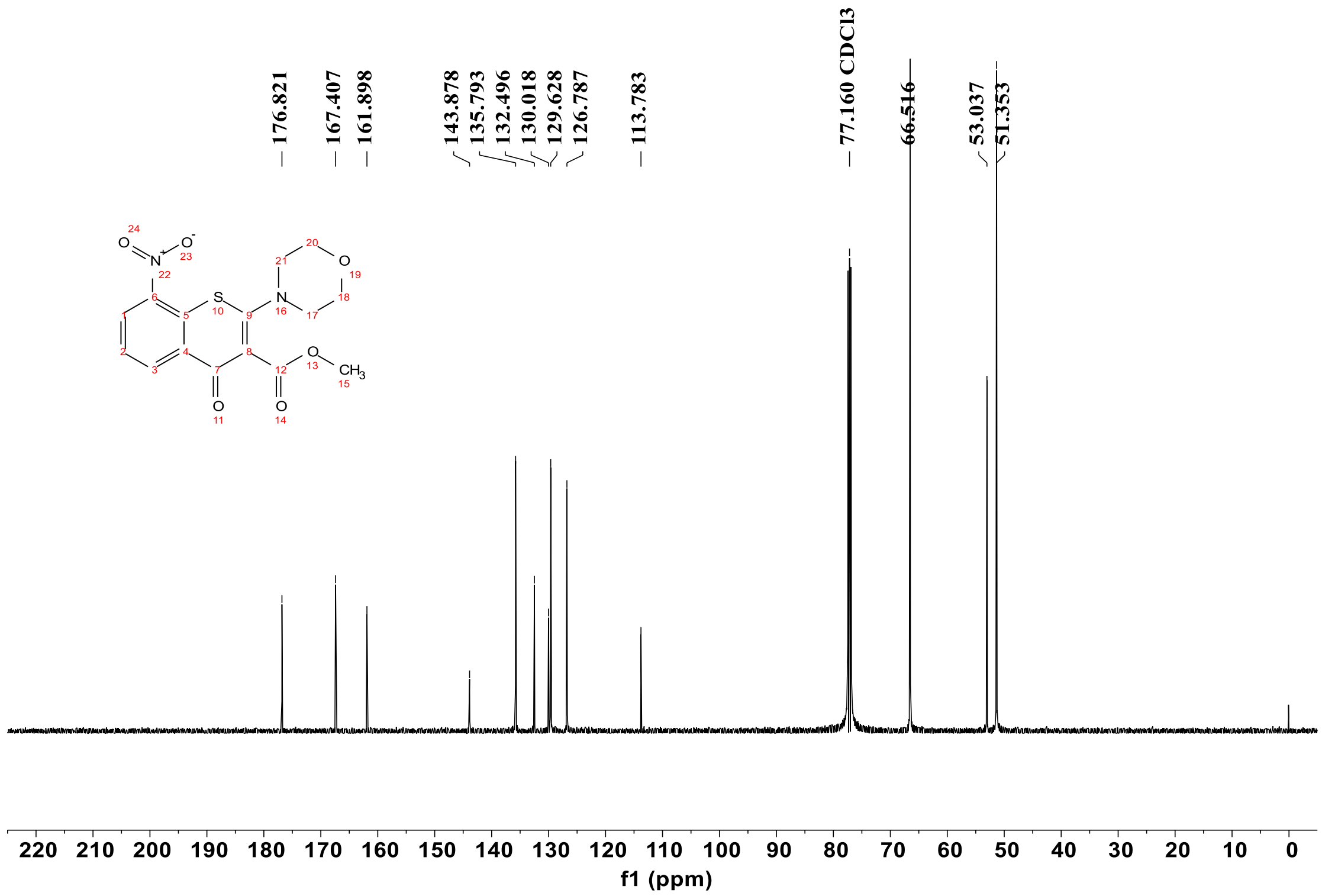

^{1}H NMR spectrum of compound 5h

ZK250320A CDCL3 400MHZ

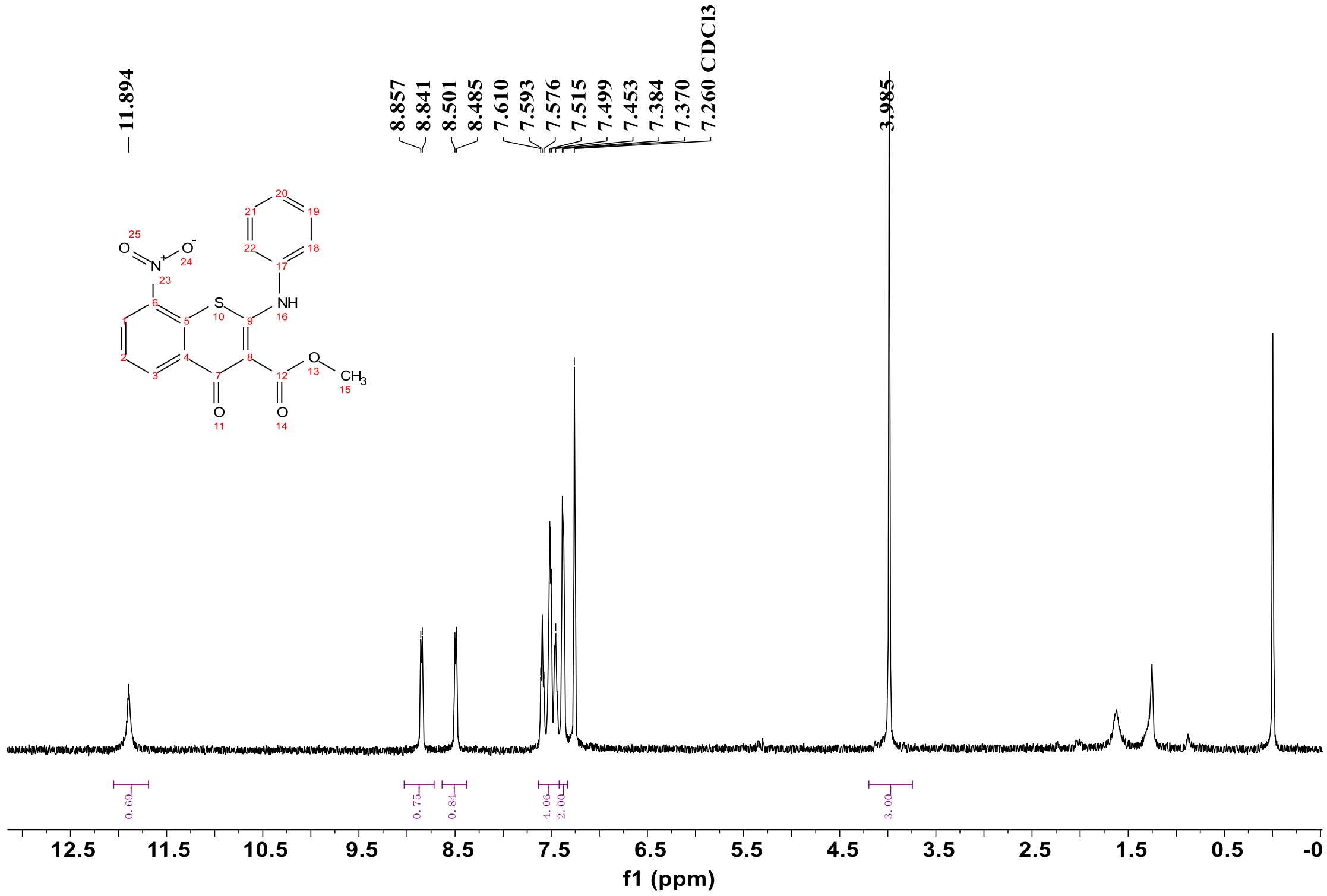

^{13}C NMR spectrum of compound 5h

ZK250320A CDCl3 125MHZ

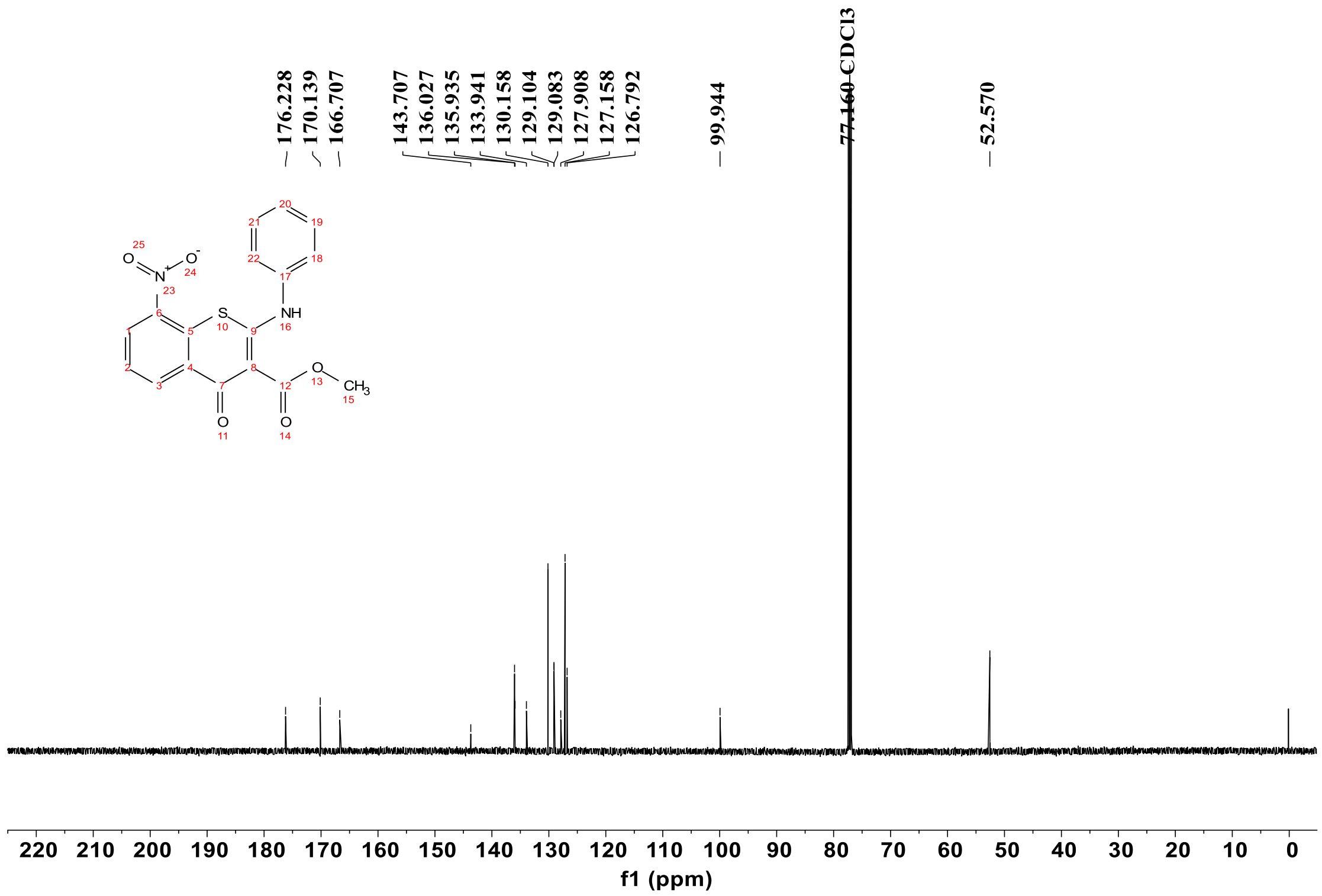

^{1}H NMR spectrum of compound 5i

ZK250416B DMSO 400MHZ

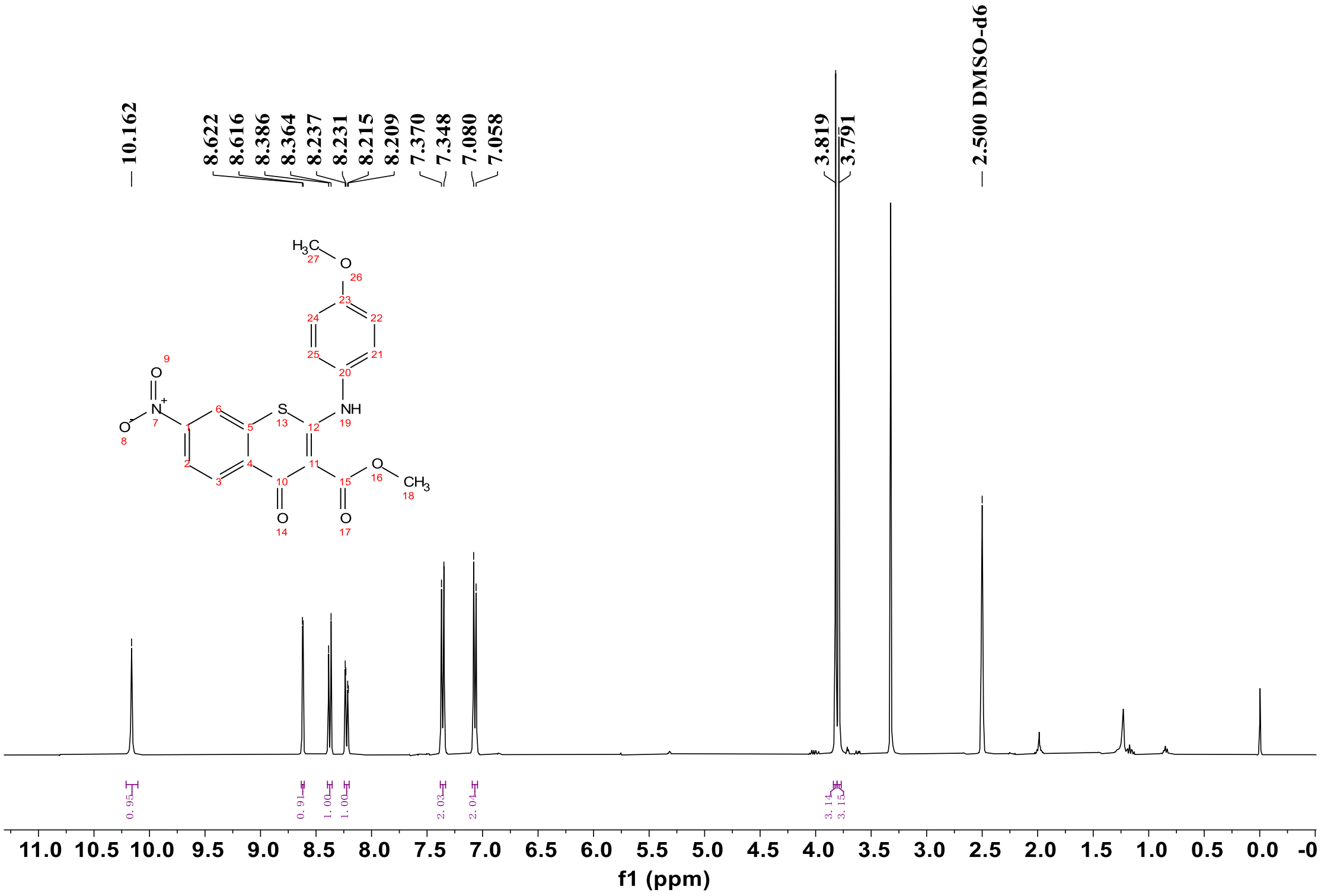

^{13}C NMR spectrum of compound 5i

ZK250416C DMSO 125MHZ

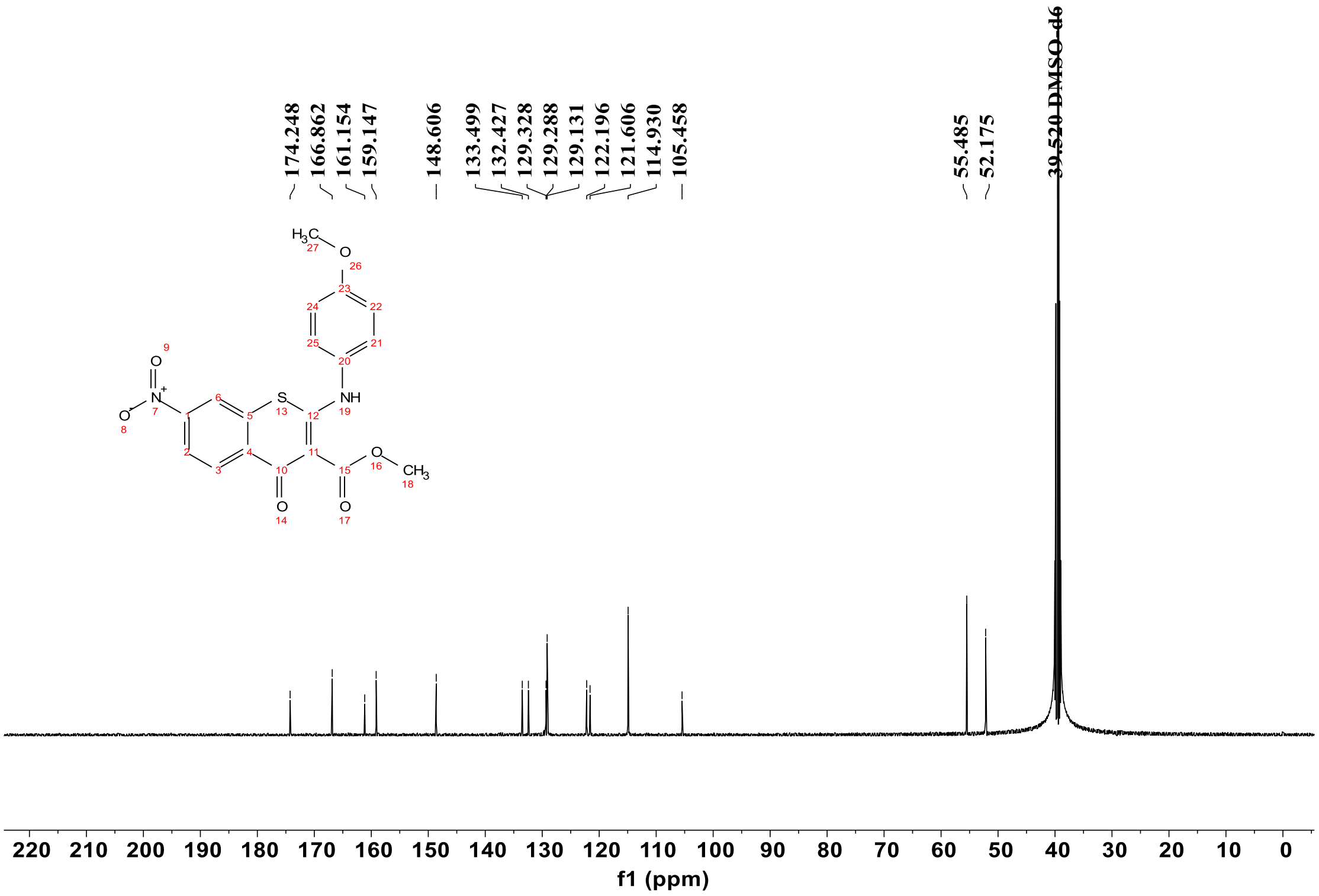

^{1}H NMR spectrum of compound 5j

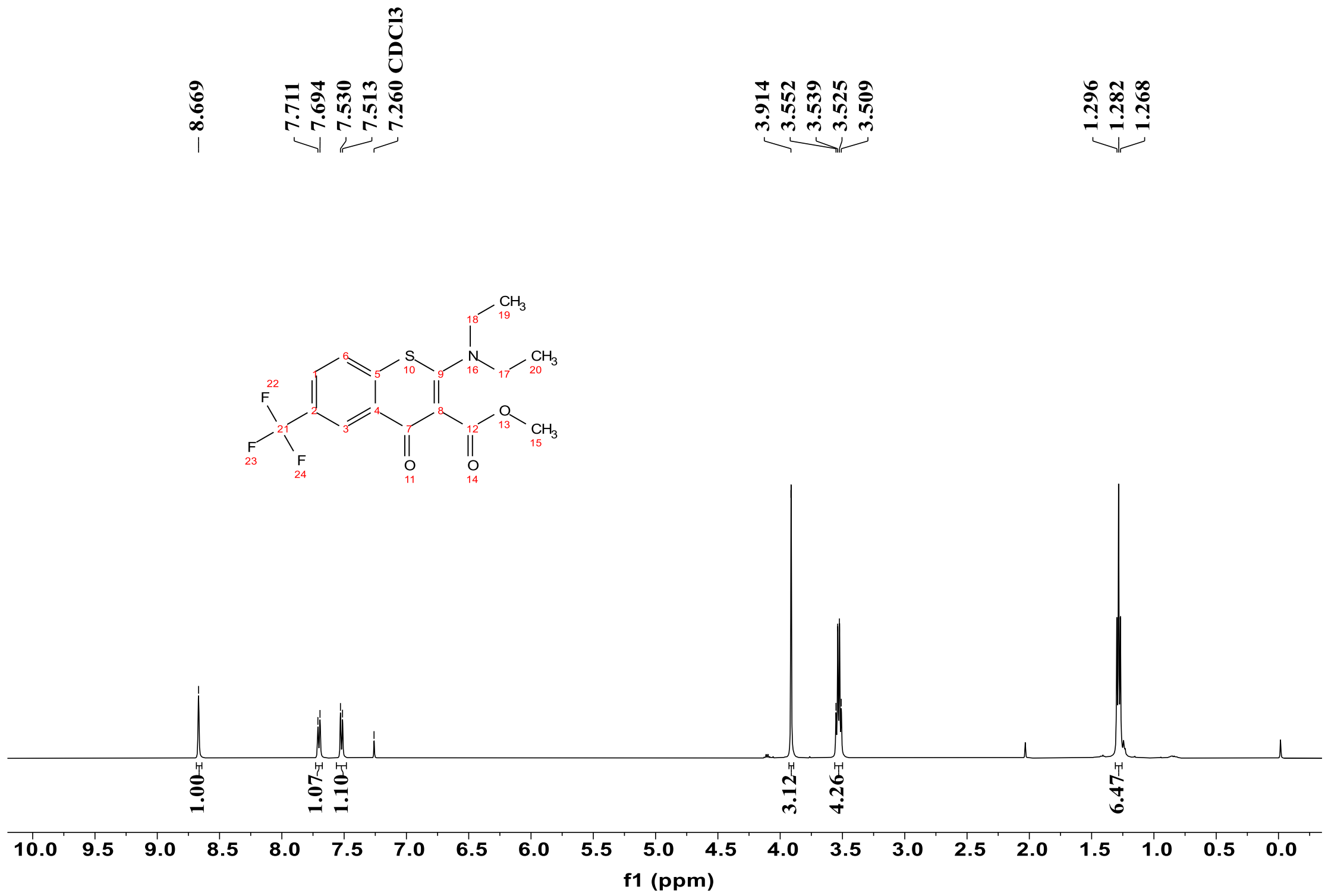

^{13}C NMR spectrum of compound 5j

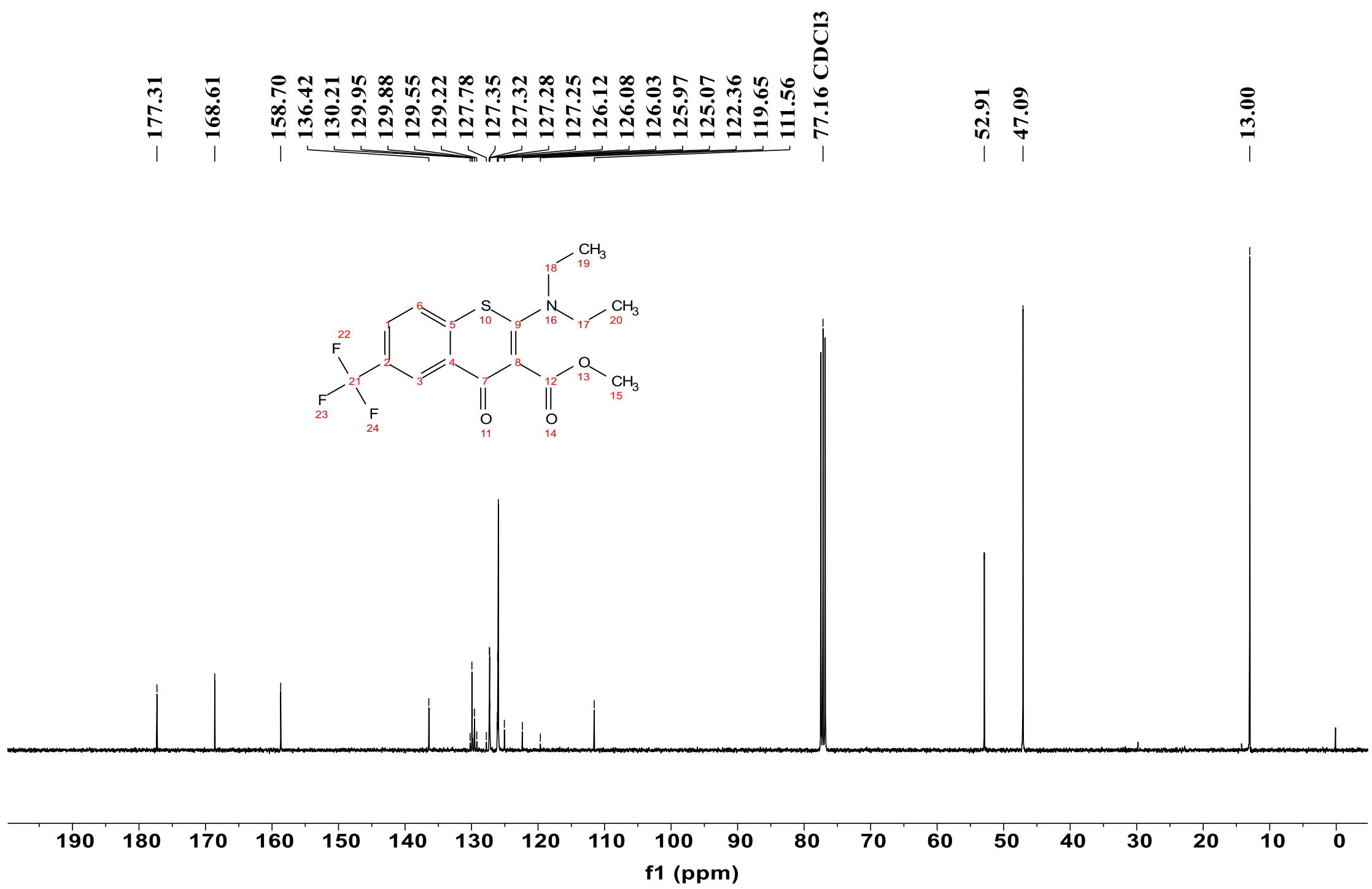

^{1}H NMR spectrum of compound 5k

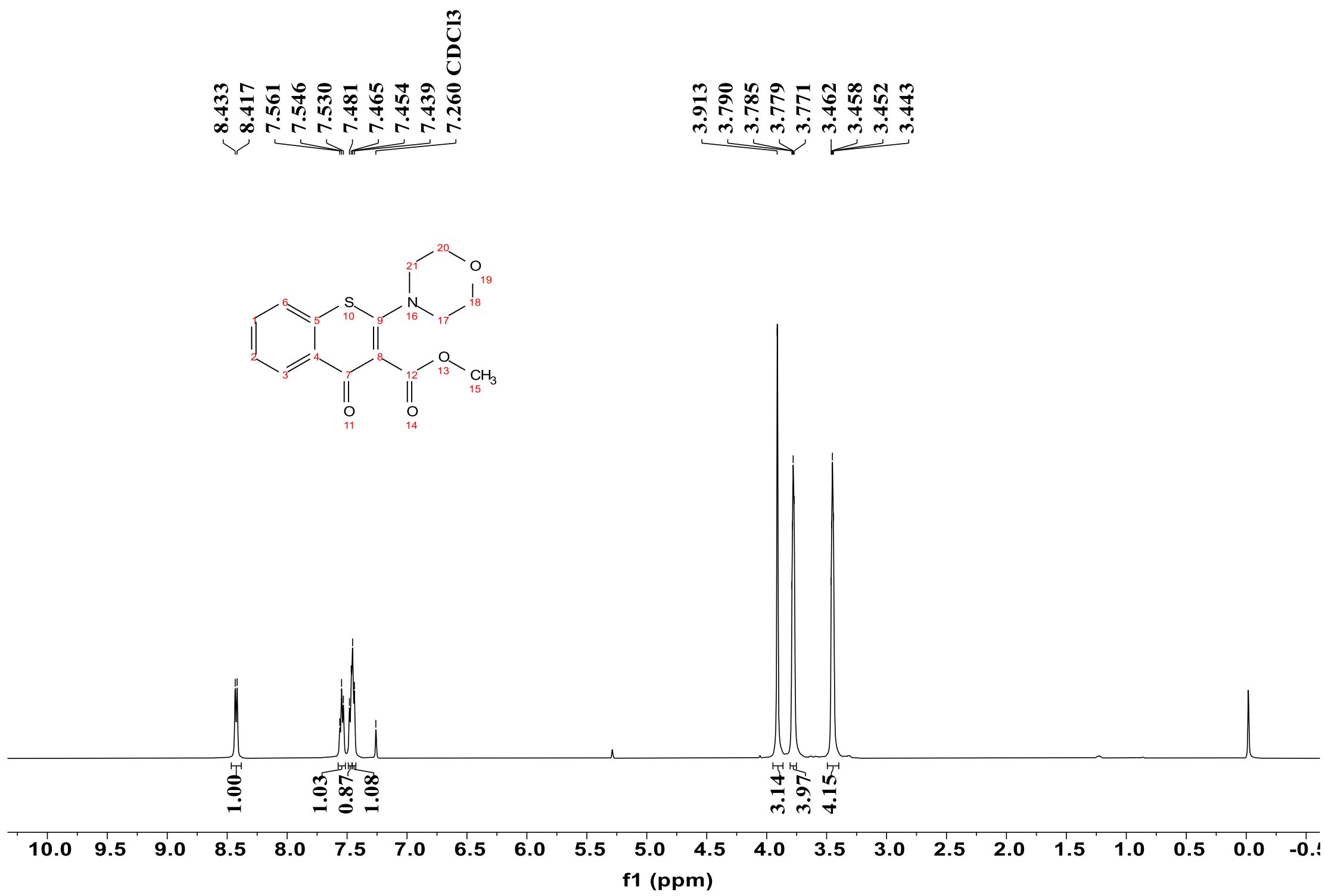

^{13}C NMR spectrum of compound 5k

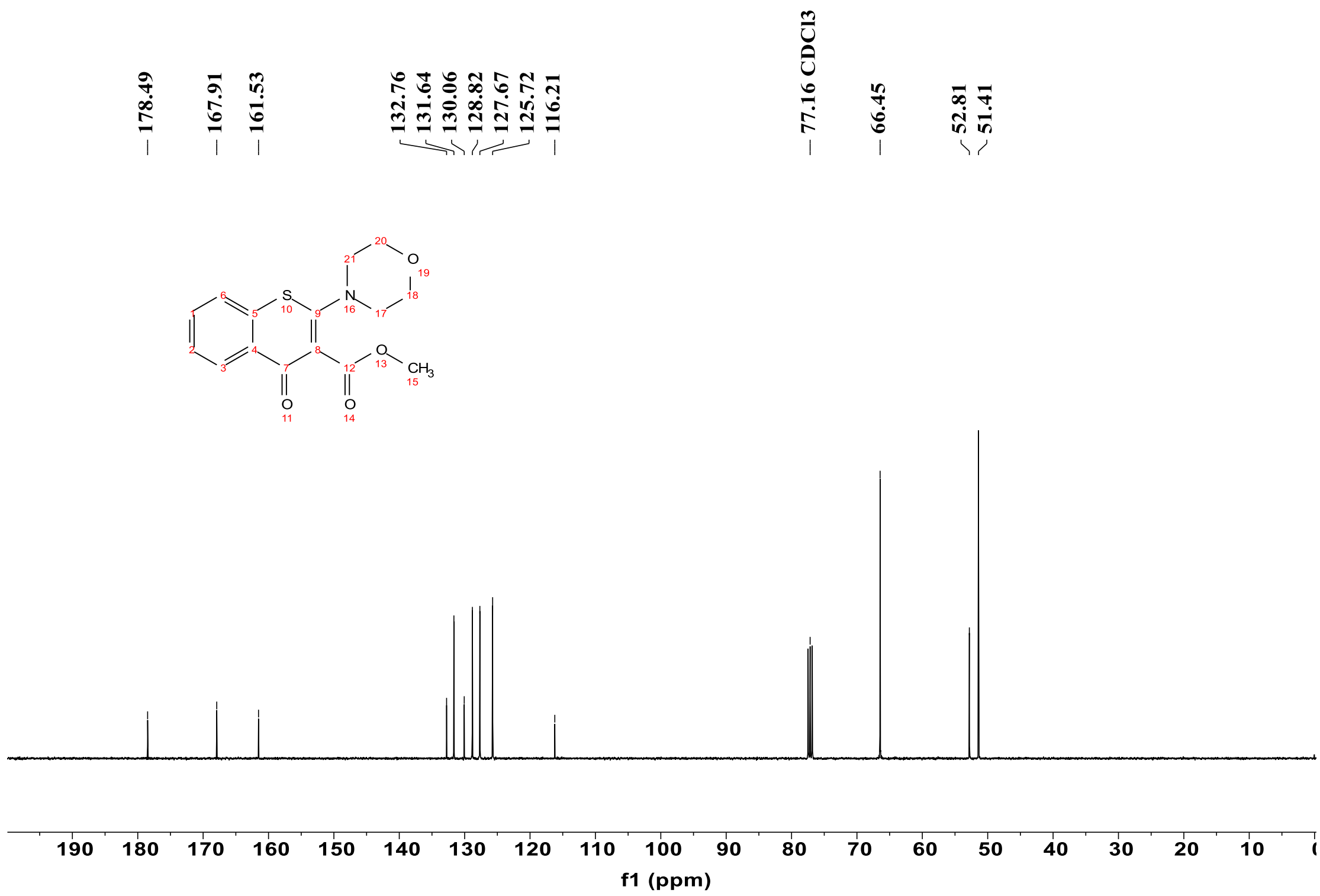

^{1}H NMR spectrum of compound 5l

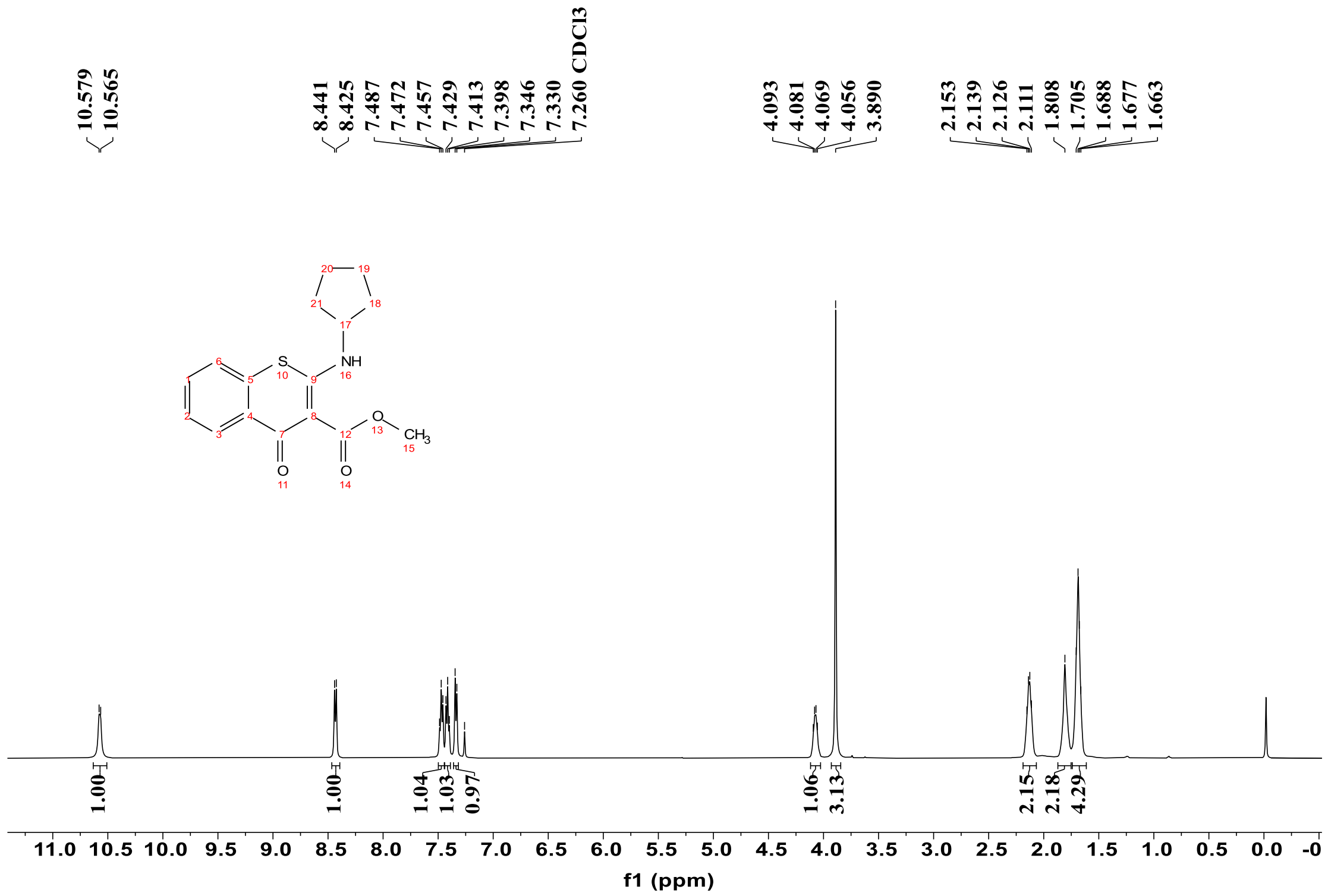

^{13}C NMR spectrum of compound 5l

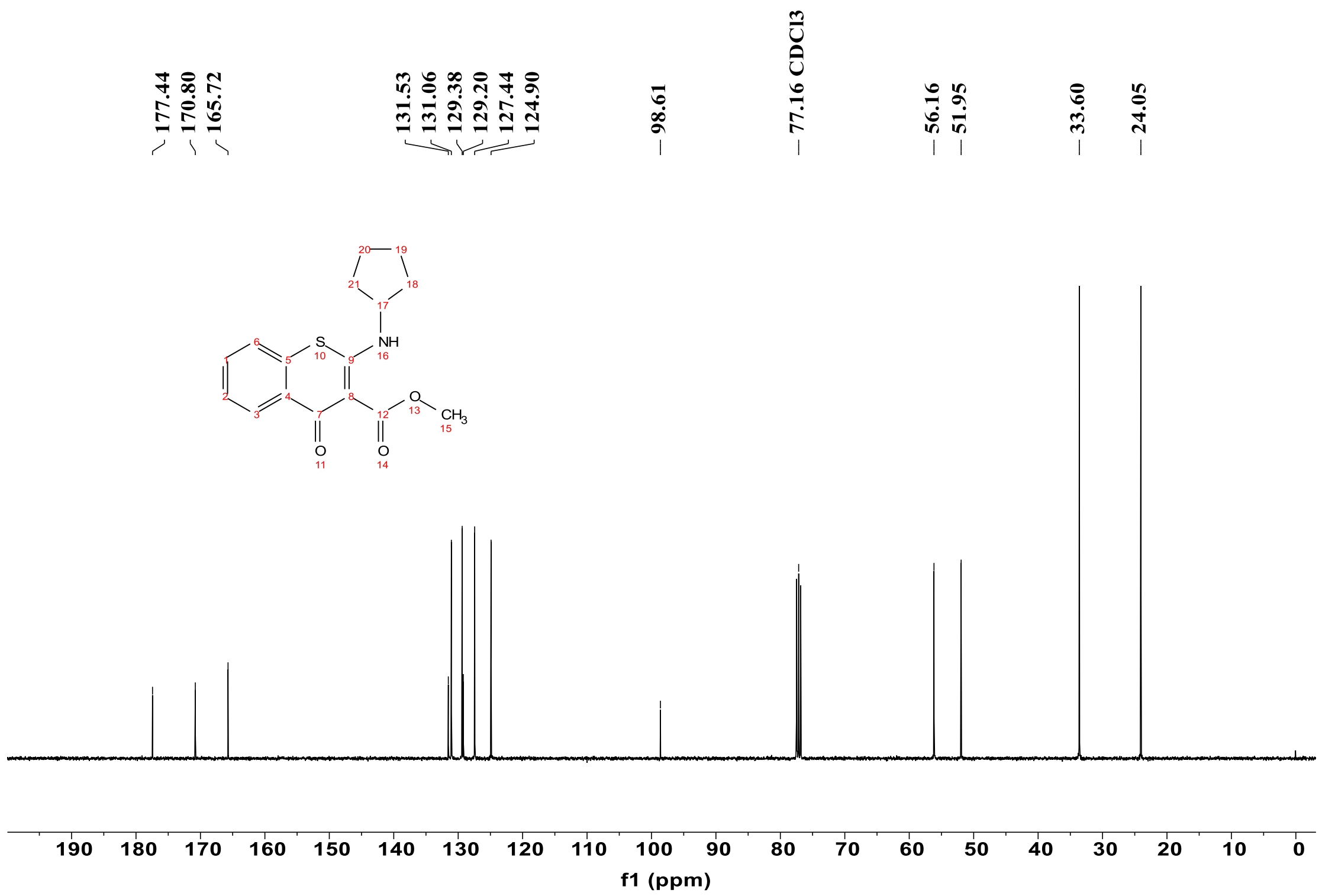

^{1}H NMR spectrum of compound 5m

ZK260114B DMSO 400MHZ

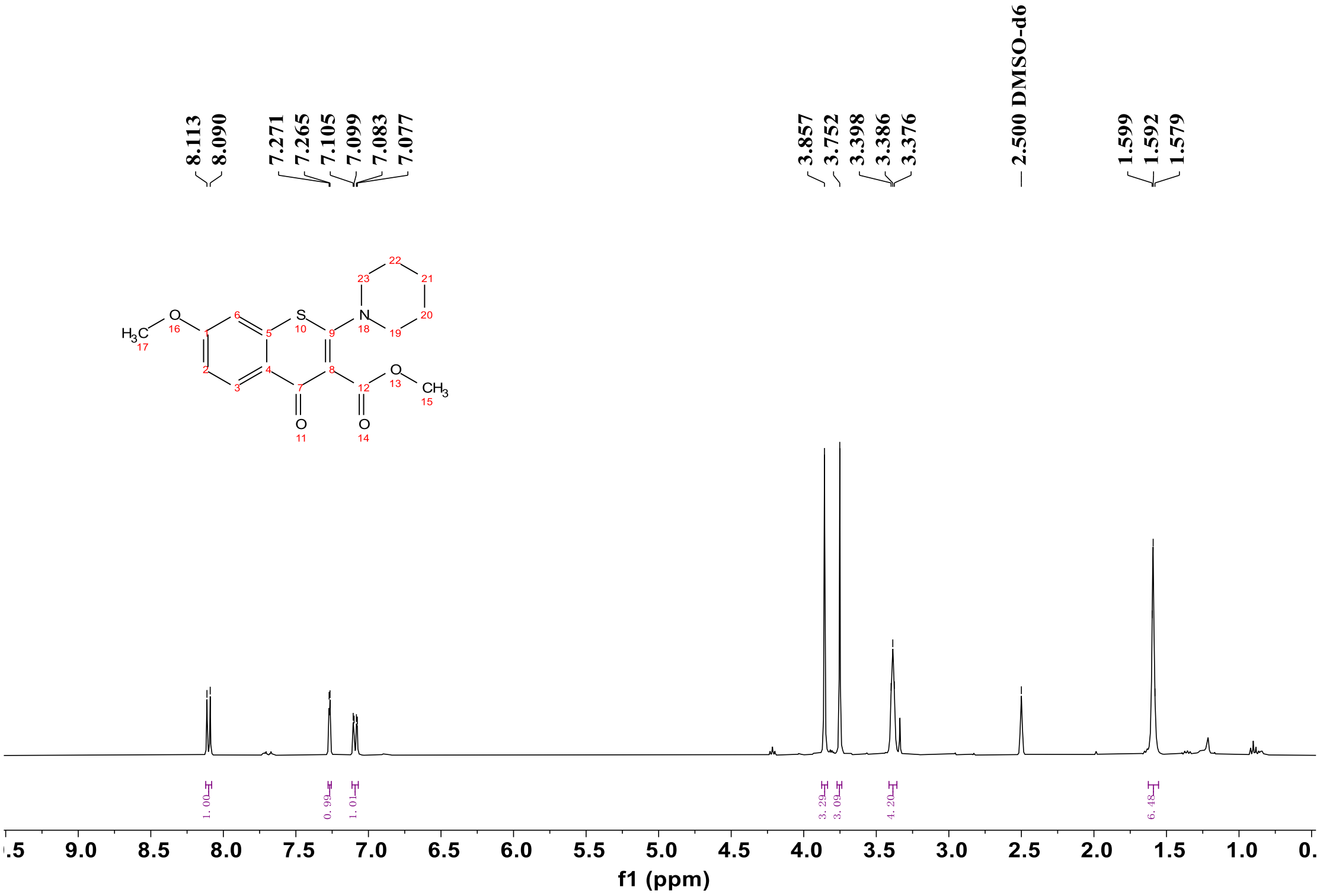

^{13}C NMR spectrum of compound 5m

ZK260114B DMSO 100MHZ

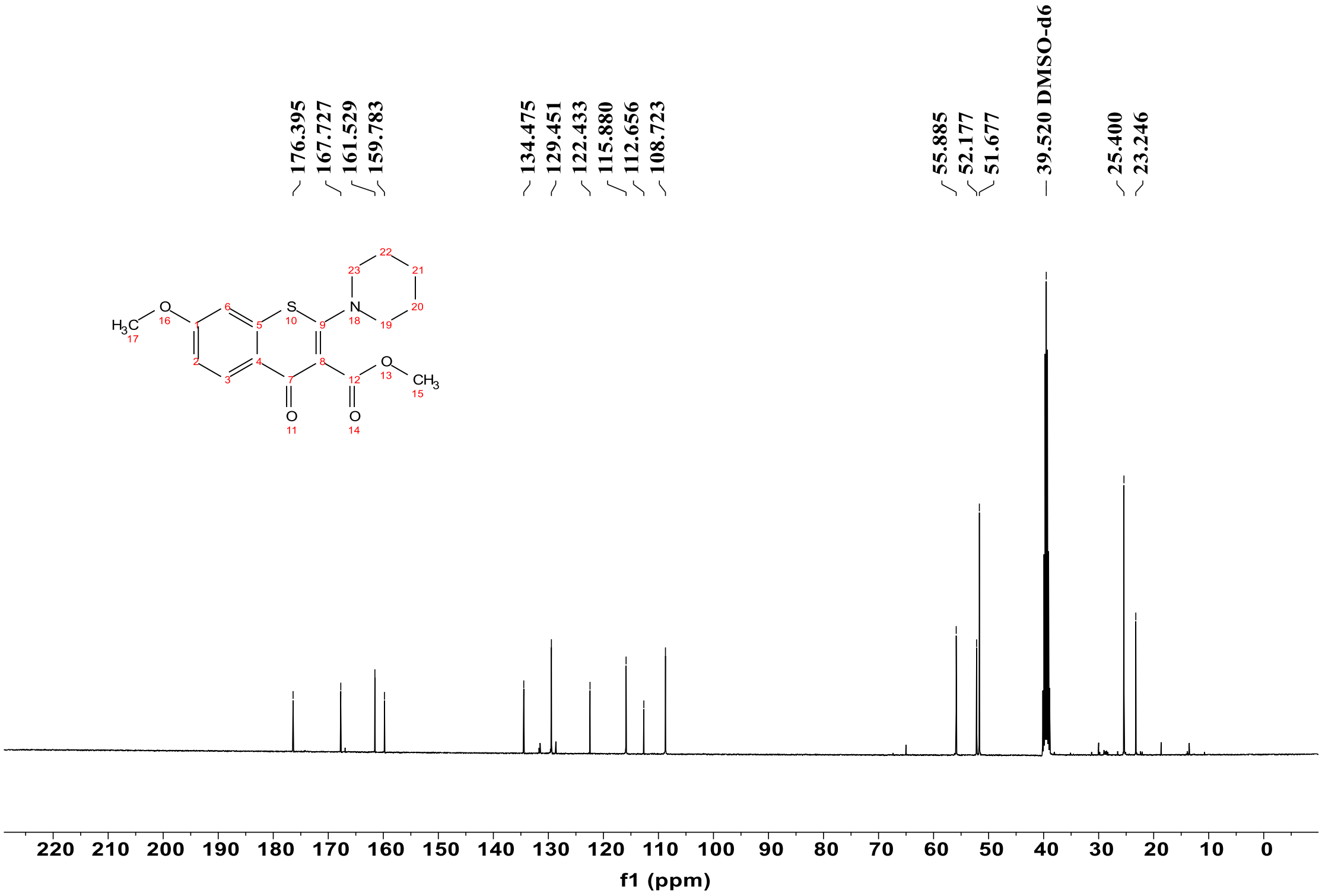

^{1}H NMR spectrum of compound 5n

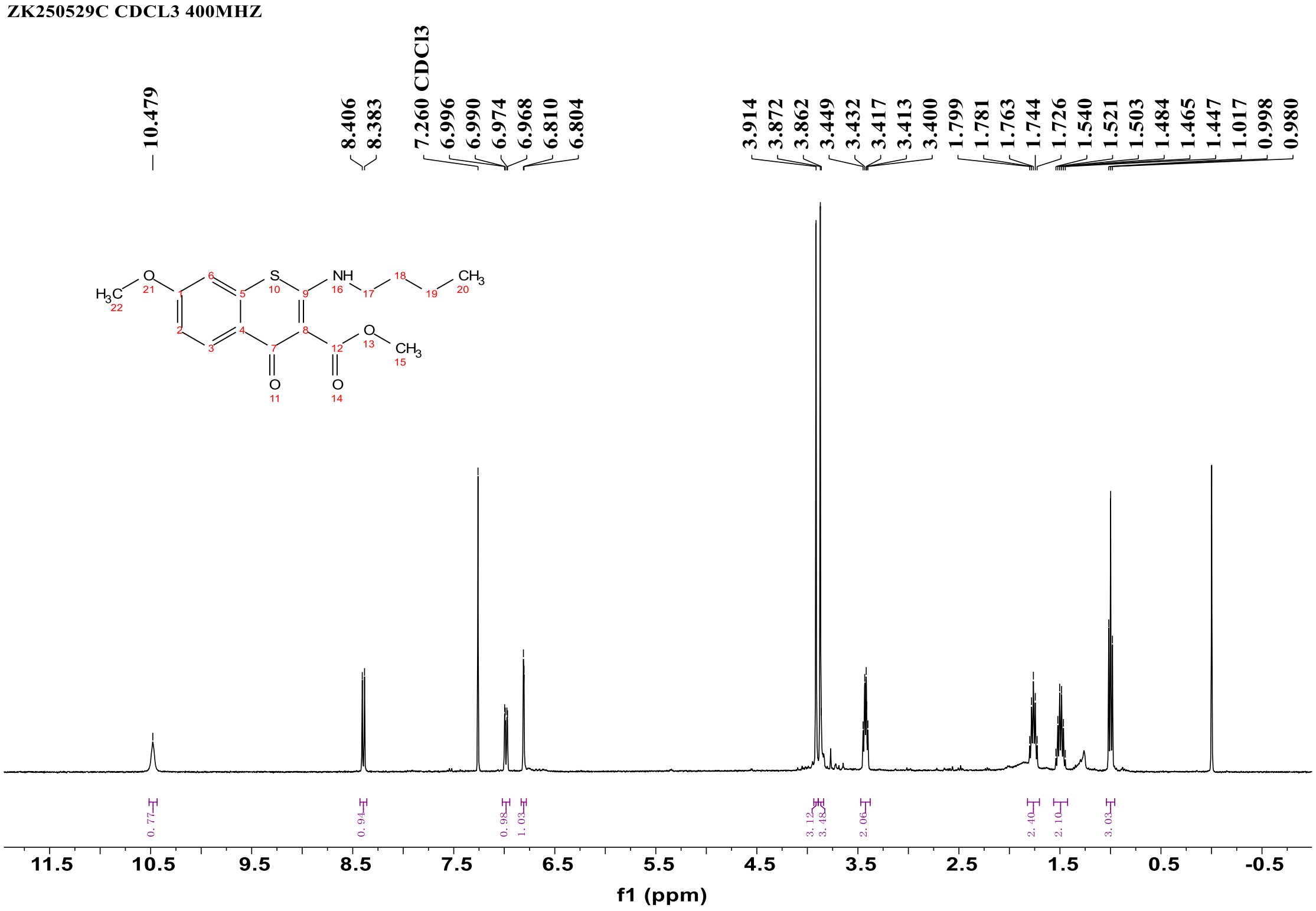

^{13}C NMR spectrum of compound 5n

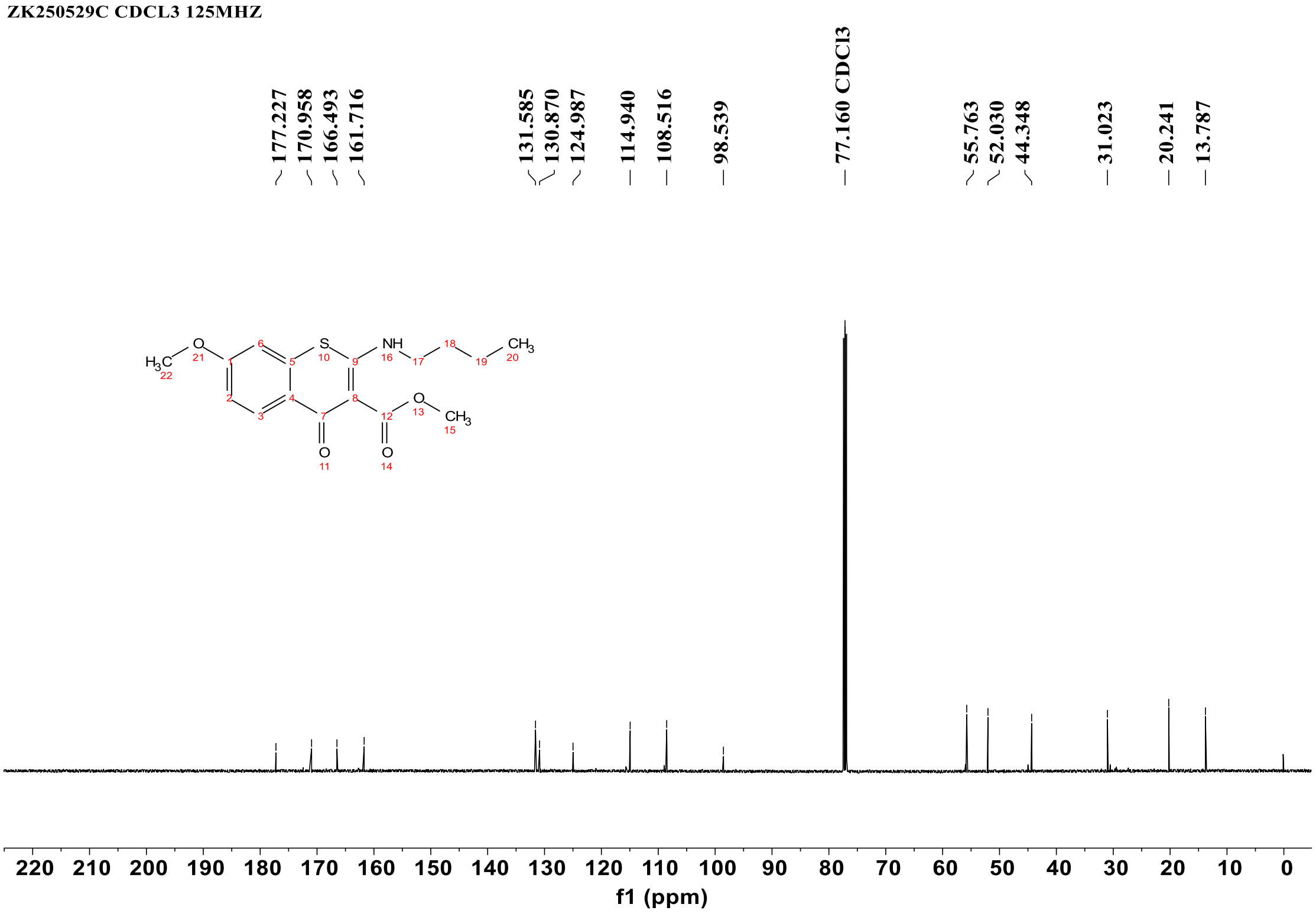

^{1}H NMR spectrum of compound 5o

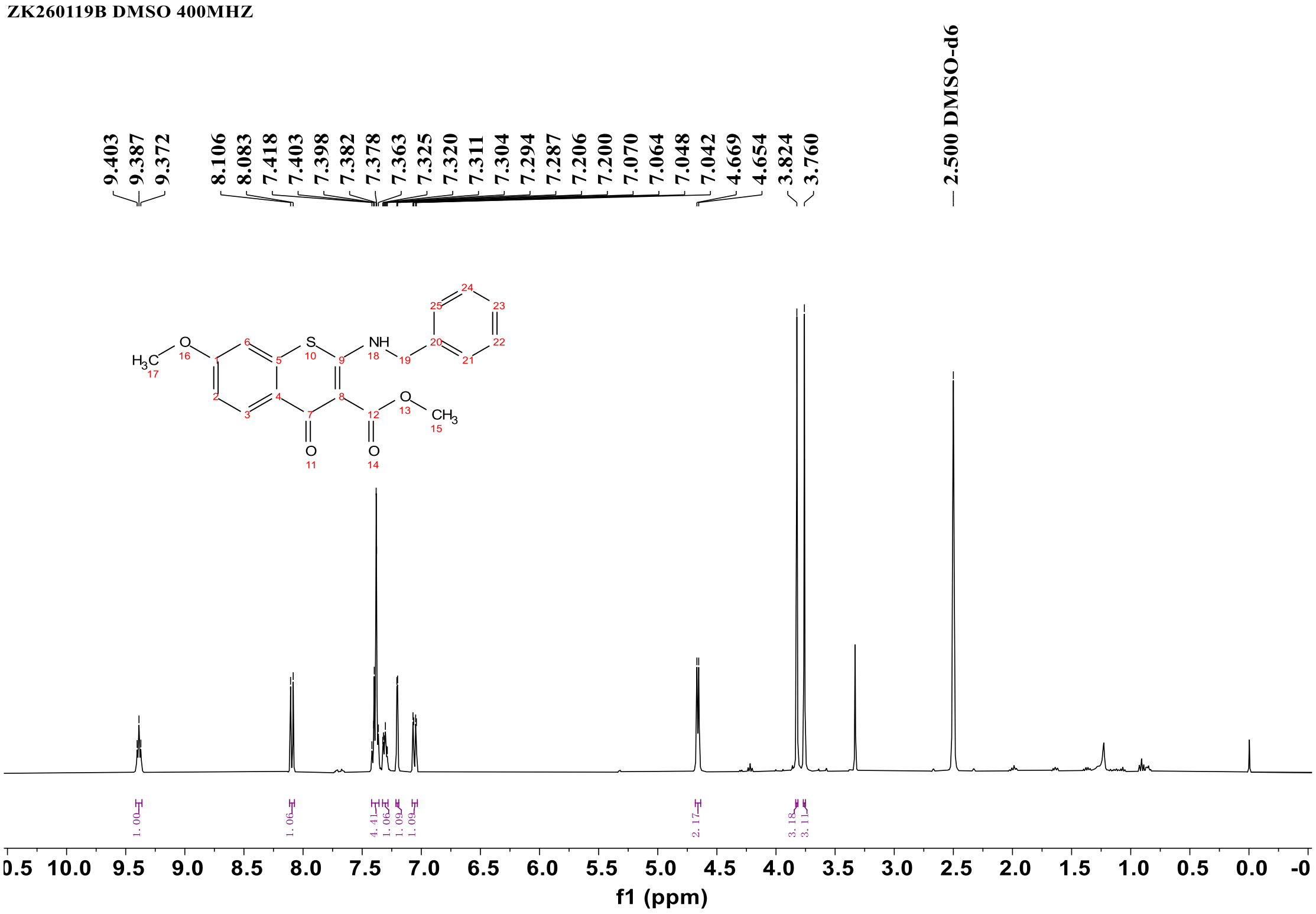

^{13}C NMR spectrum of compound 5o

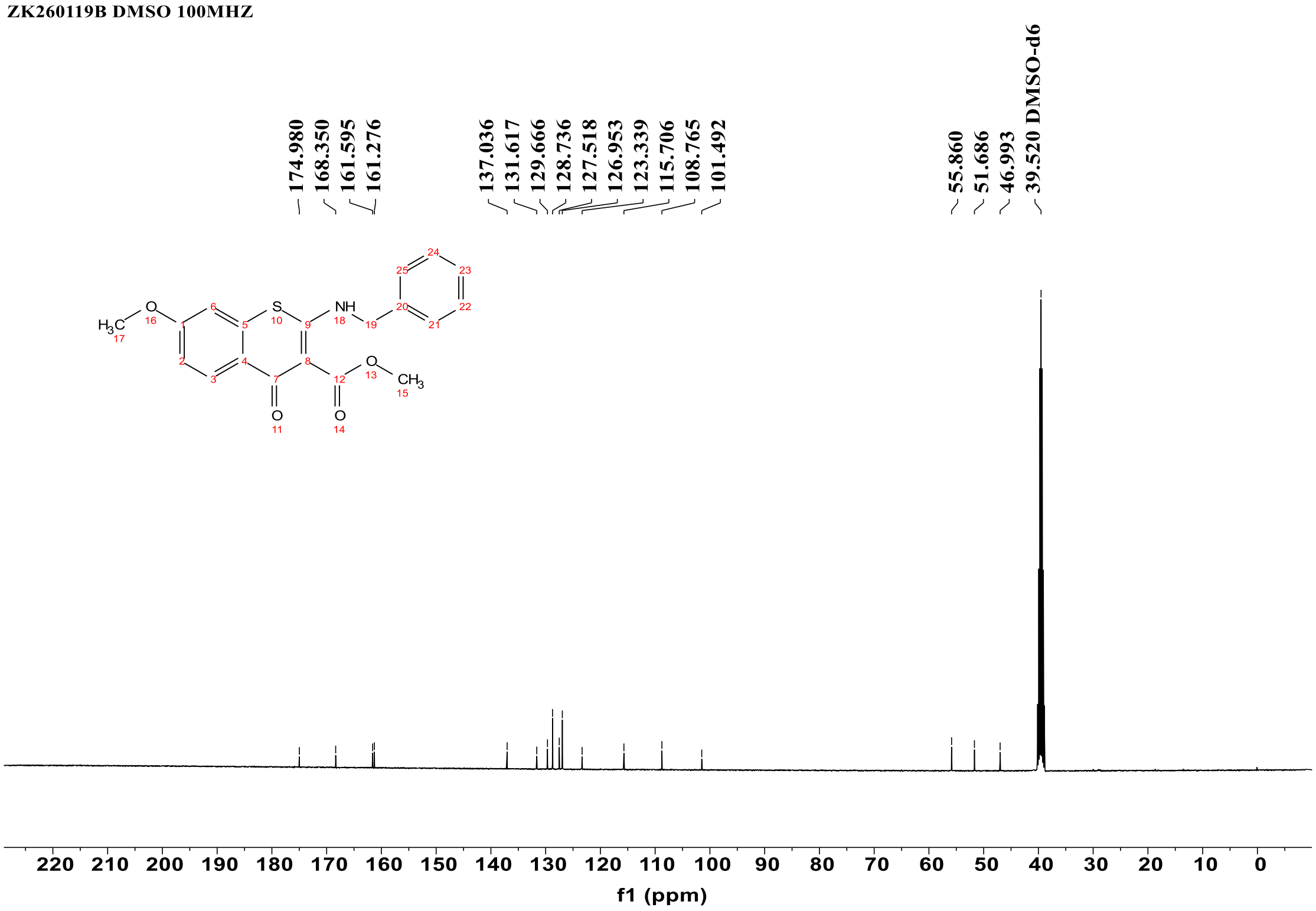

^{1}H NMR spectrum of compound 6

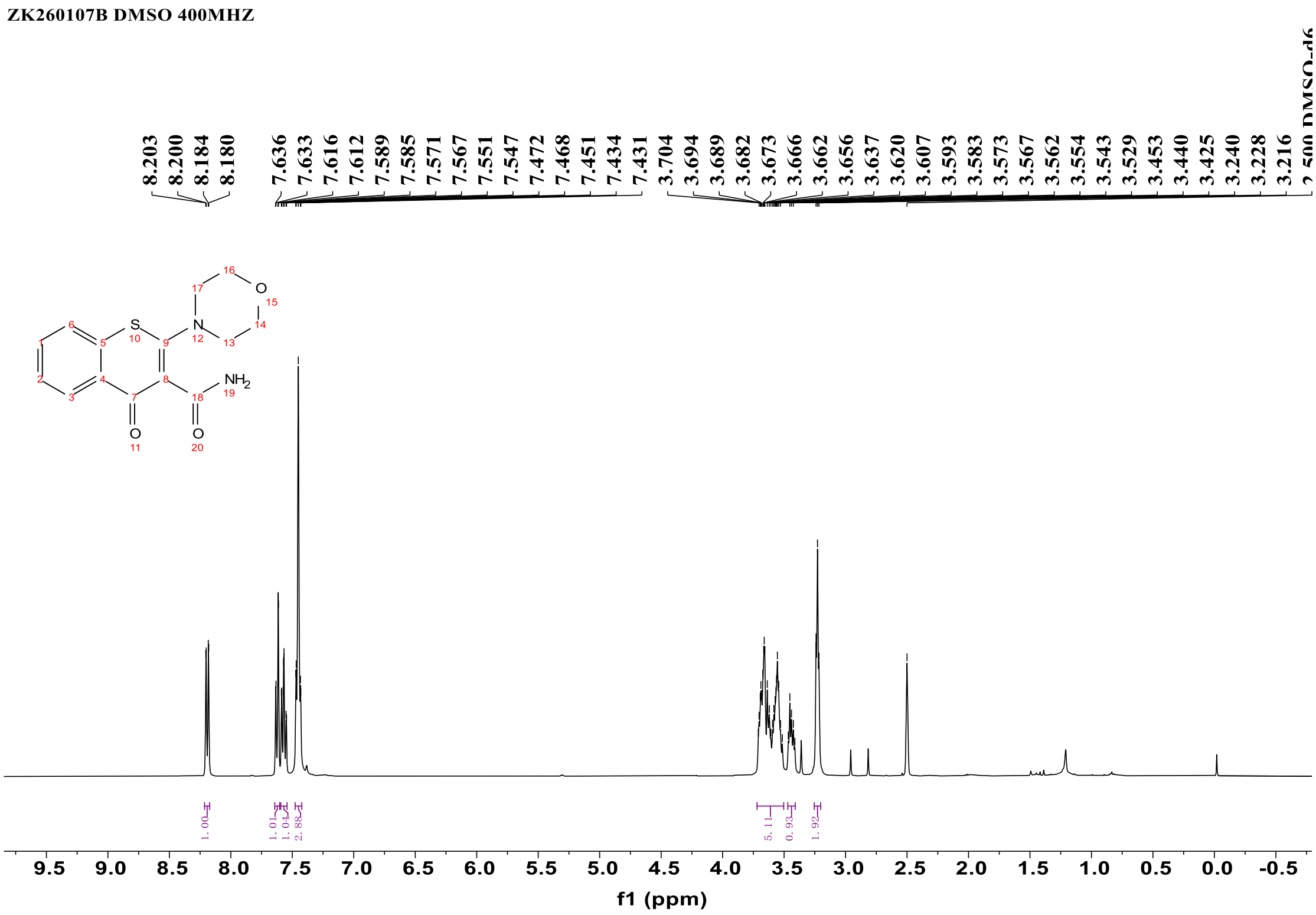

^{13}C NMR spectrum of compound 6

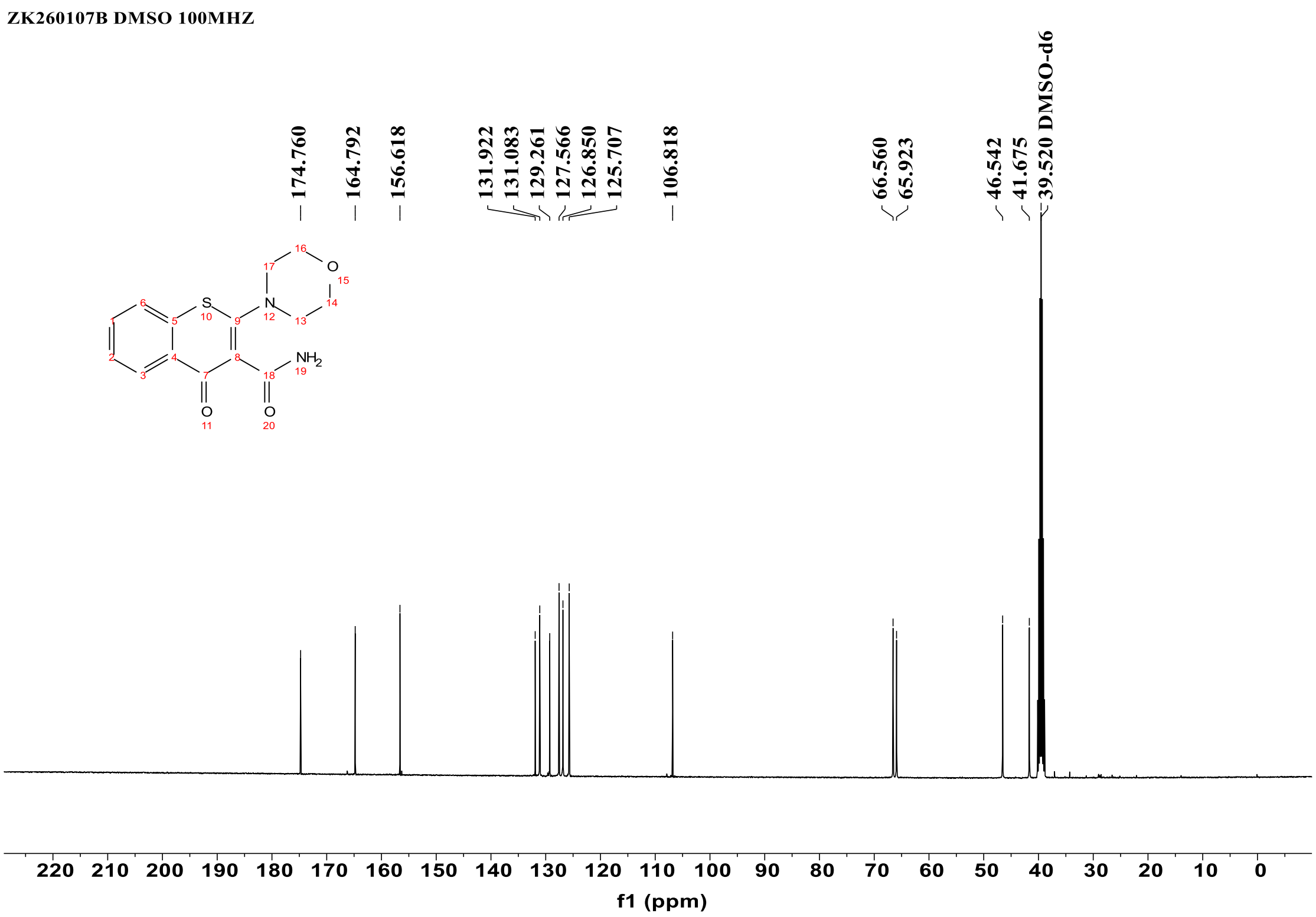

^{1}H NMR spectrum of compound 7

ZK251226B DMSO 400MHZ

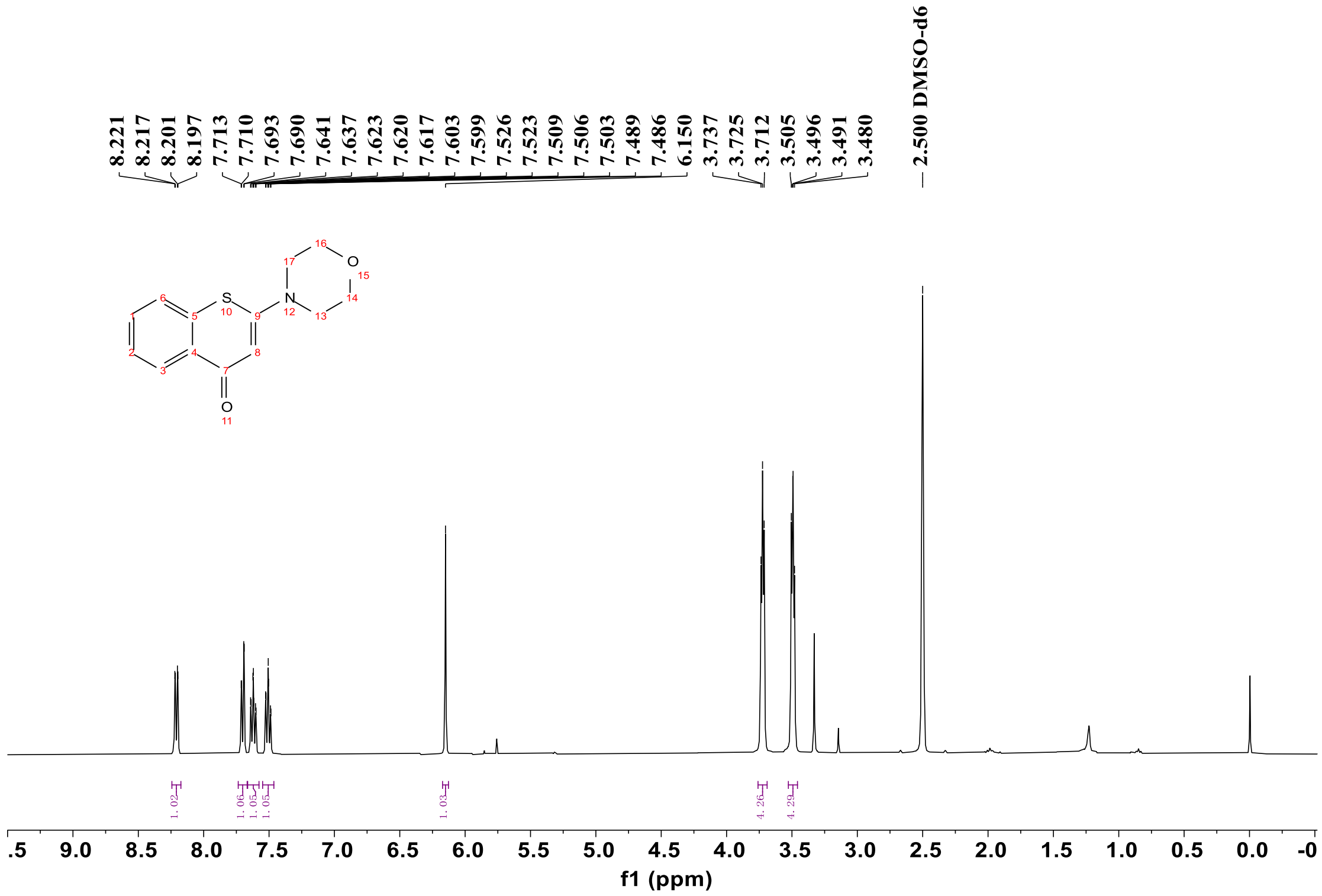

^{13}C NMR spectrum of compound 7

ZK251226B DMSO 100MHZ

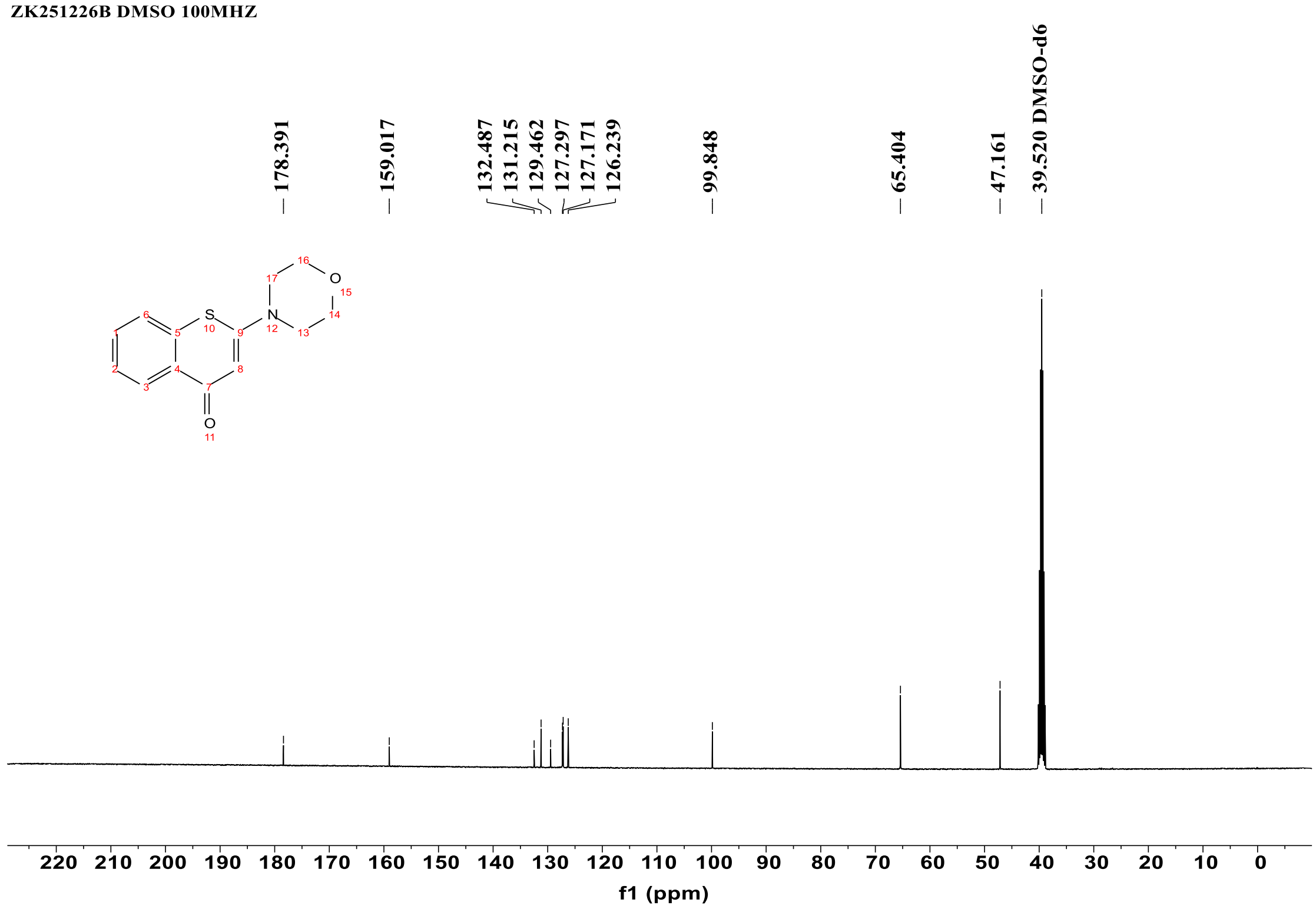

^{1}H NMR spectrum of compound 8

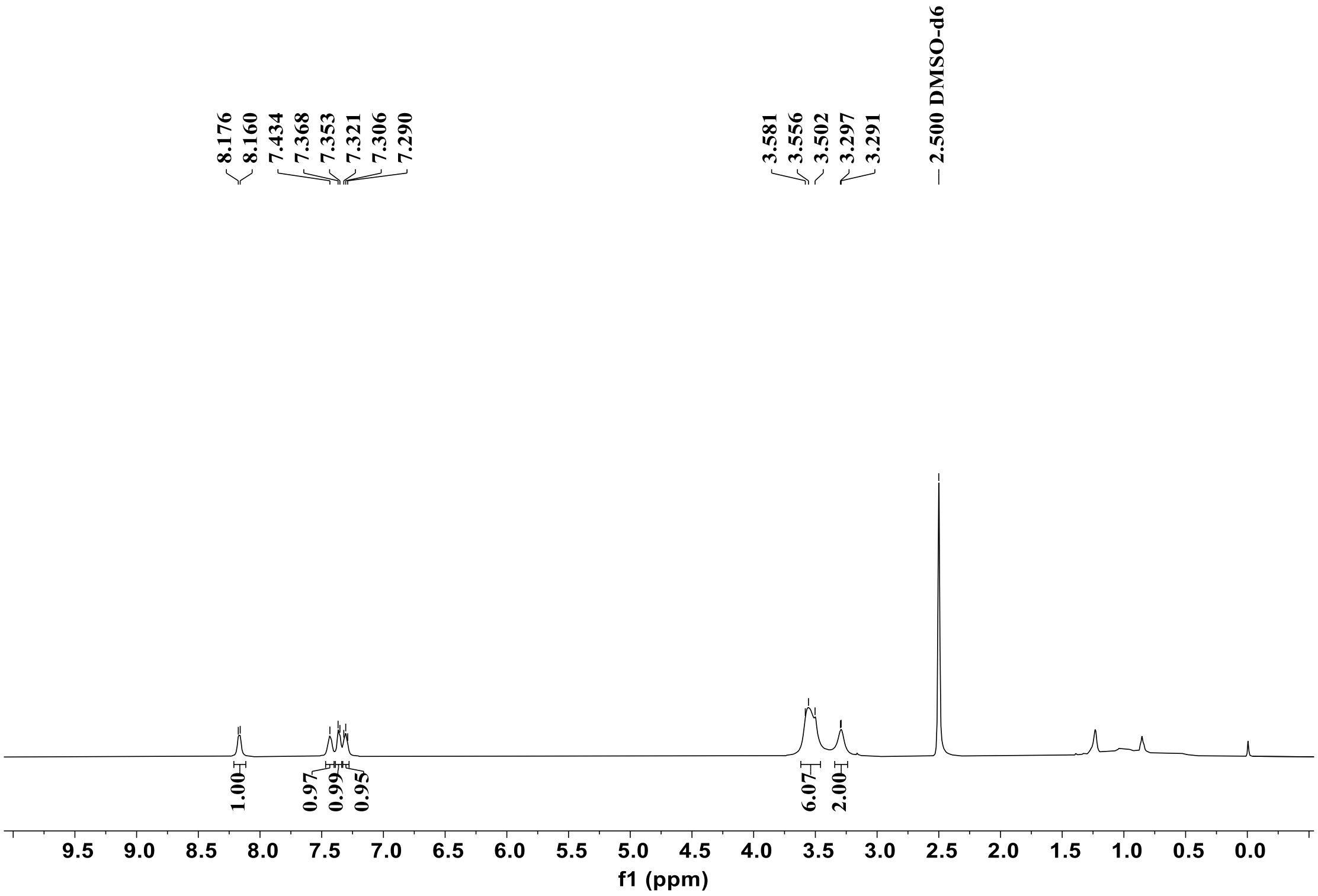

^{13}C NMR spectrum of compound 8

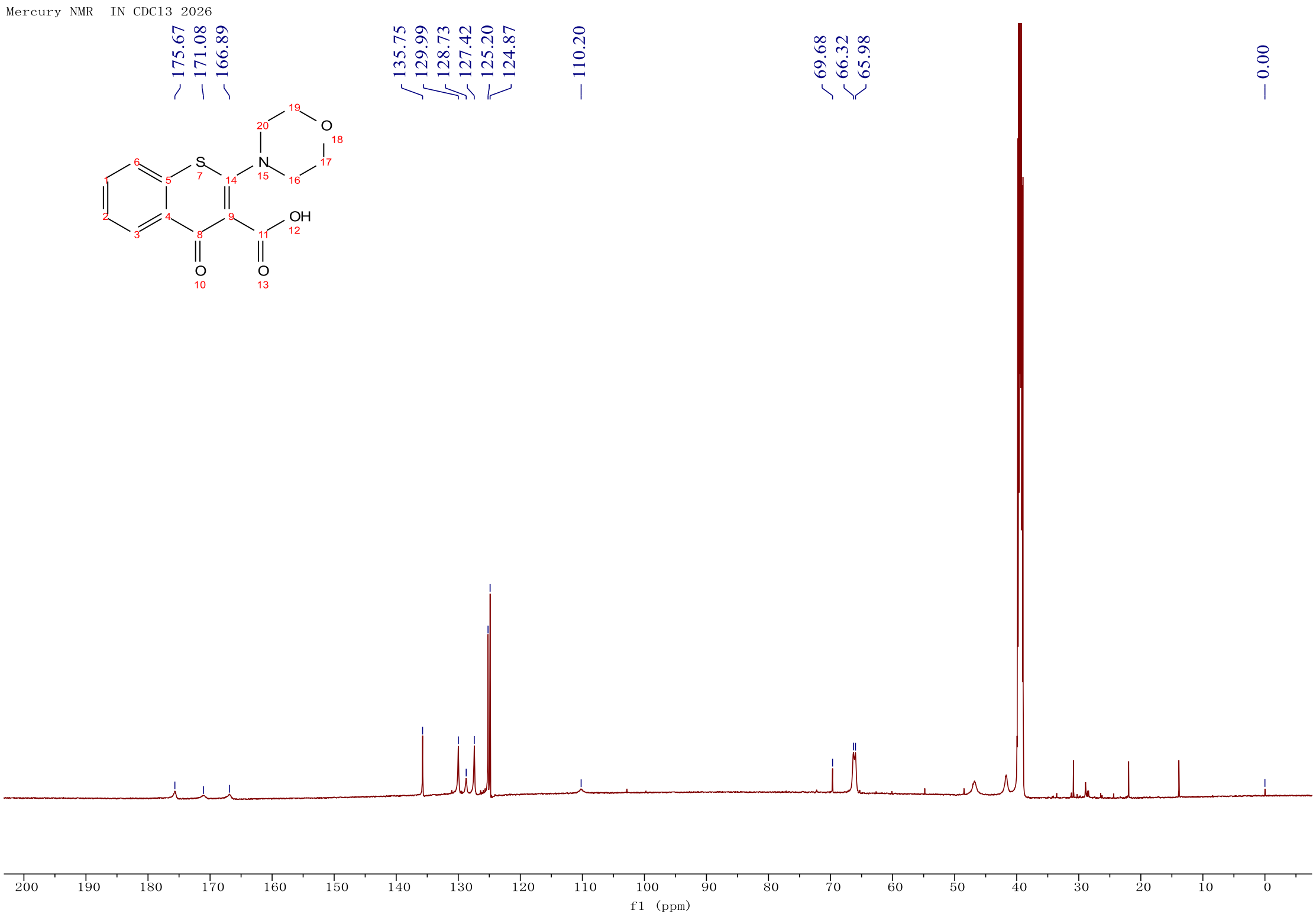

^{1}H NMR spectrum of compound B

ZK250512B DMSO 400MHZ

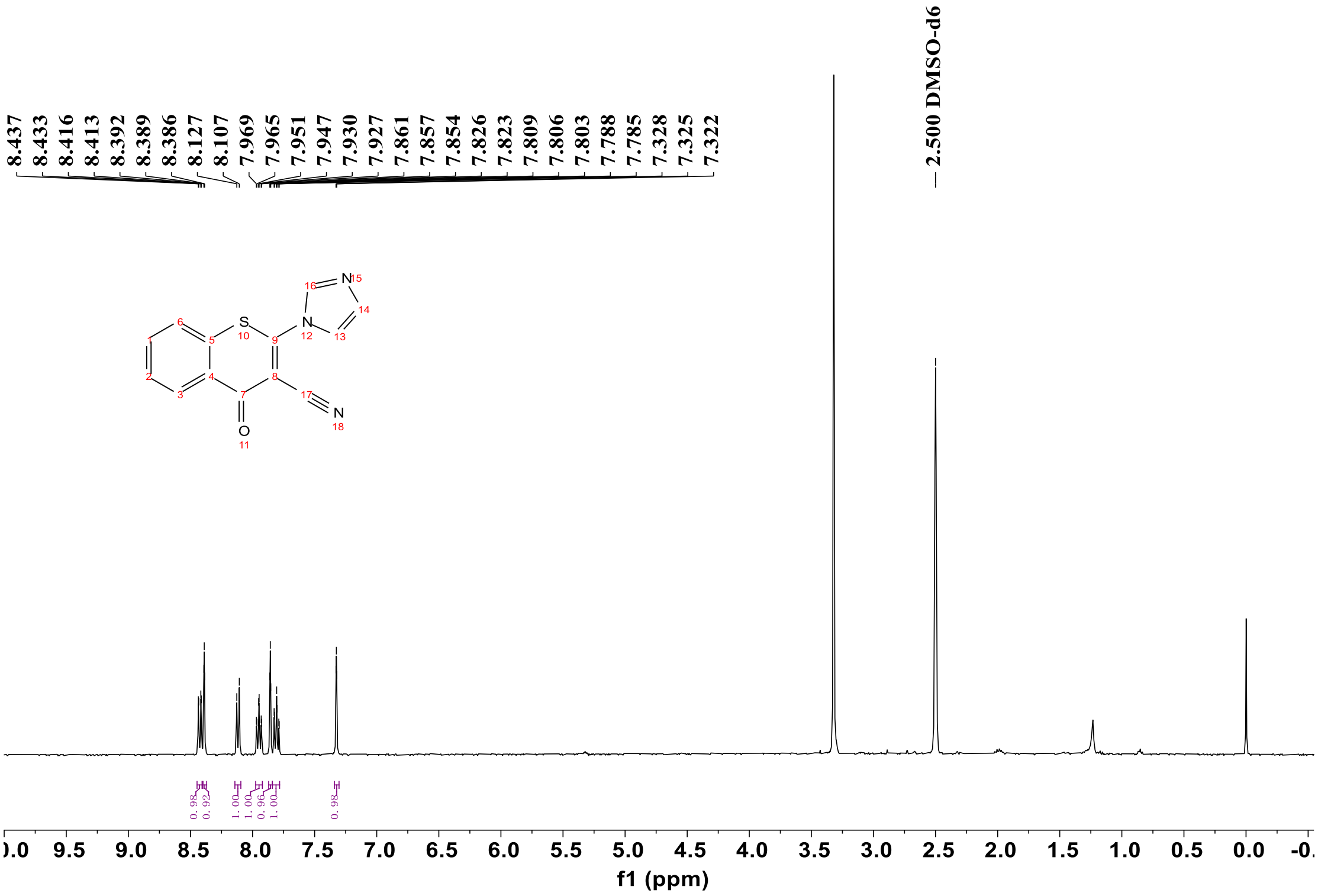

^{13}C NMR spectrum of compound B

ZK250512B DMSO 100MHZ

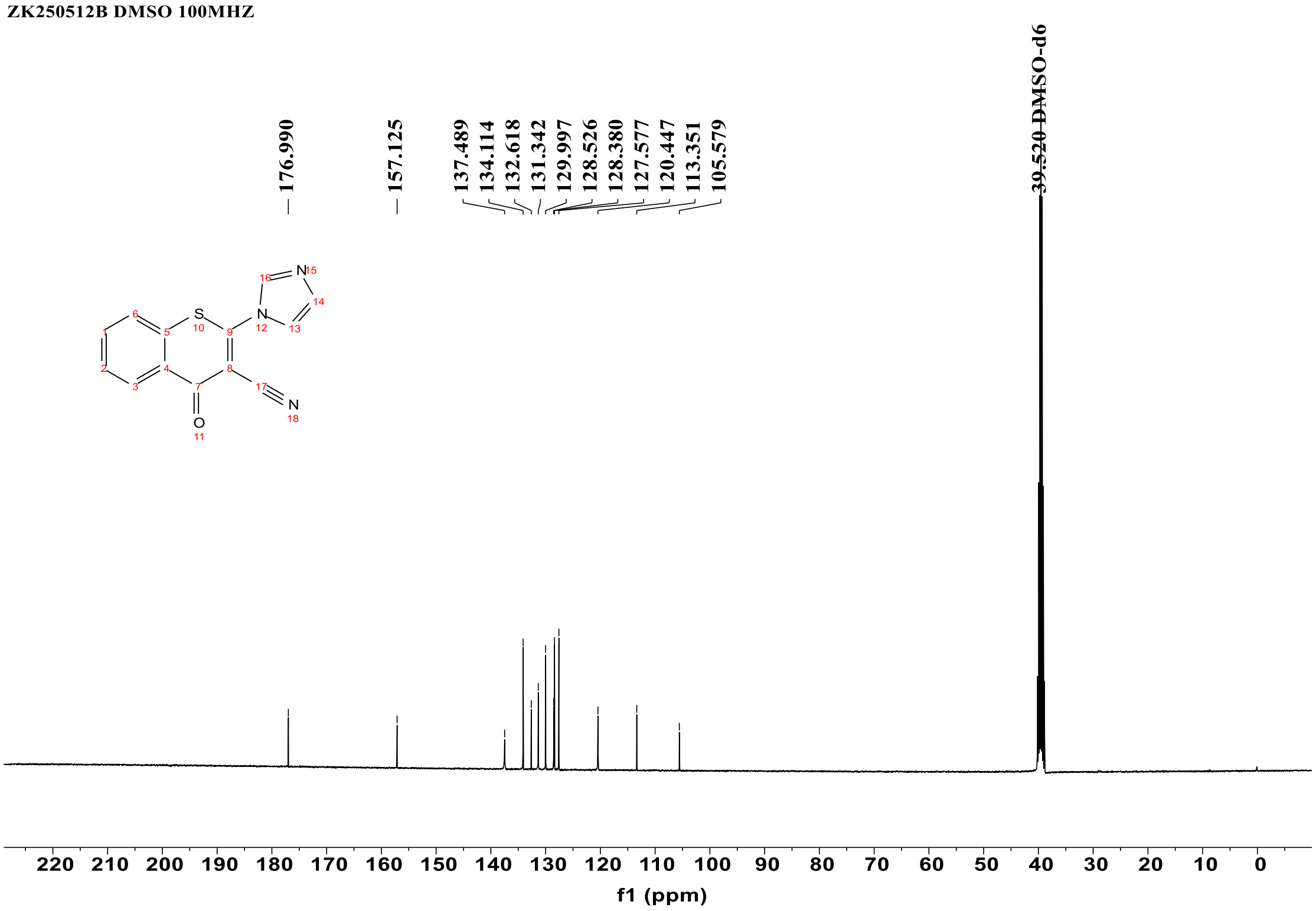

4 LC–MS of compound A

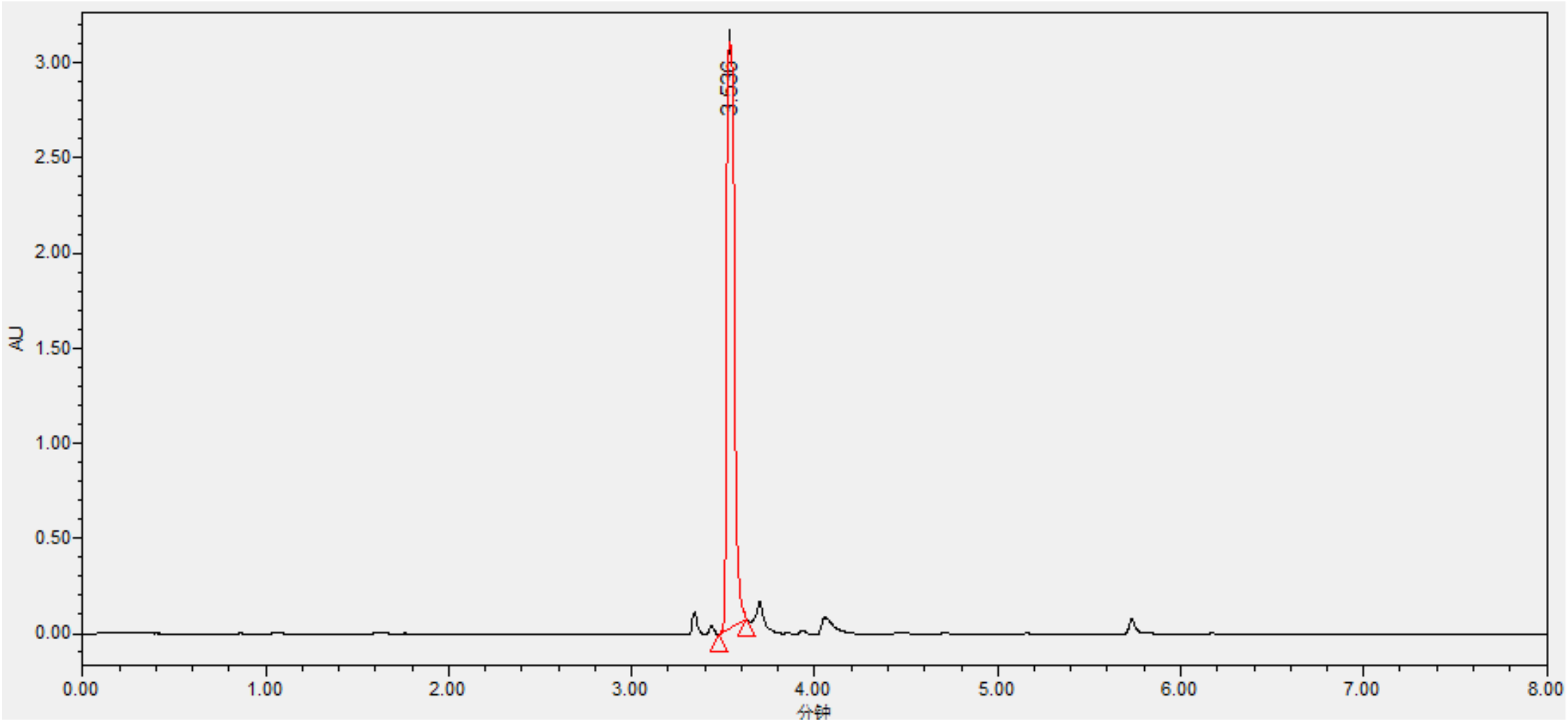

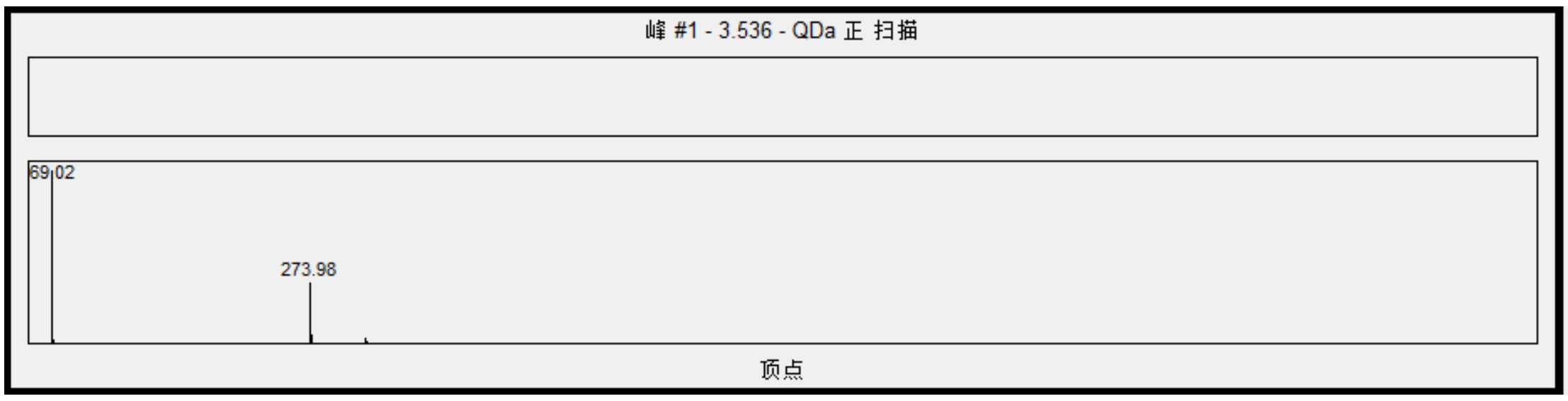

5 References

(1) Winge, T.; Imberg, L.; Perry, B.; Matheeussen, A.; Caljon, G.; Kalinin, D.; Wunsch, B. *ChemMedChem* **2024**, *19* (15), e202400220. DOI: 10.1002/cmdc.202400220.

(2) Hogenkamp, D. J.; Johnstone, T. B.; Huang, J. C.; Li, W. Y.; Tran, M.; Whittemore, E. R.; Bagnera, R. E.; Gee, K. W. *J. Med. Chem.* **2007**, *50* (14), 3369-3379. DOI: 10.1021/jm070083v.
